# Synthesis of Chromenoimidazoles, Annulated with an Azaindole Moiety, through a Base-Promoted Domino Reaction of Cyano­methyl Quaternary Salts

**DOI:** 10.1055/s-0036-1589496

**Published:** 2017-04-04

**Authors:** Leonid G. Voskressensky, Olga A. Storozhenko, Alexey A. Festa, Roman A. Novikov, Alexey V. Varlamov

**Affiliations:** a Peoples’ Friendship University of Russia (RUDN University)Miklukho-Maklaya St. 6, 117198 MoscowRussian Federationlvoskressensky@sci.pfu.edu.ru; b Engelhardt Institute of Molecular Biology, Russian Academy of SciencesVavilova 32, 119334 MoscowRussian Federation

**Keywords:** azaindoles, domino reaction, isogranulatimides, iminium salts, chromenoimidazoles, microwave

## Abstract

The reactivity of *N* -cyanomethyl quaternary salts of 4-, 5- and 7-azaindoles towards salicylic aldehydes has been studied. The interaction of azaindolium salts with salicylic aldehydes proceeds as a base-promoted domino reaction, giving the corresponding chromenoimidazopyrrolopyridines. In the case of the 7-(cyanomethyl)-7-azaindolium salt, the reaction was found to be more sensitive, but the use of the 1-methyl-substituted salt allowed the synthesis of the desired compounds, incorporating the heterocyclic core of isogranulatimide C, a marine natural product.

Imidazoles annulated with a pyrrolopyridine (azaindole) moiety are known for their antiviral 1 and antitumor 2 activities. An imidazopyrrolopyridine fragment appears in the isogranulatimides (Figure 1 ), marine natural products isolated from the Brazilian ascidian *Didemnum granulatum* , which show high inhibitory activity against the G2 DNA damage checkpoint, the kinases Chk1 (IC _50_ = 0.1 μM) and GSK-3 beta 3 and various other kinases. 4 Their analogues also exhibit high antiproliferative 5 and Chk1 inhibition 6 activities.

**Figure 1:**
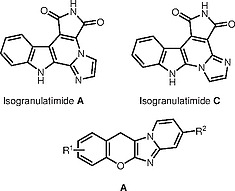
Structures of isogranulatimide A, C and chromenoimidazopyridines **A**

Imidazoles annulated with chromenes **A** (Figure 1 ) have recently been characterized as cytotoxic agents against HCT116 cancer cells due to their ability to induce cell cycle arrest and apoptosis without significant effects on normal cells. 7

Synthetic approaches toward the imidazopyrrolopyridine core of isogranulatimides are usually based on the construction of the pyridine ring. 8 The current project involves the preparation of imidazopyrrolopyridines fused with a chromene moiety through a base-promoted domino reaction of isomeric *N* -(cyanomethyl)azaindolium salts with *o* -hydroxybenzaldehydes, creating the imidazole and pyran cycles in an effective manner. 9


**Scheme 1** Interaction of cyanomethyl salts of 6-azaindole with *o* -hydroxybenzaldehydes


Recently, preliminary studies of 6-(cyanomethyl)pyrrolo[2,3- *c* ]pyridinium salt reactivity showed the possibility of transforming such salts into chromenoimidazopyrrolopyridines, incorporating the heterocyclic core of isogranulatimide A (Scheme 1 ). 10 The potential of this reaction in the construction of different isomeric chromenoimidazopyrrolopyridines, among other things comprising the isogranulatimide C heterocyclic core, is the subject of this paper.

First, *N* -(cyanomethyl)azaindolium salts **1** – **3** were prepared by alkylation of the corresponding heterocycles with chloroacetonitrile or bromoacetonitrile in acetonitrile (Scheme 2 ). The yields for compounds **2** and **3** were 80% and 83%, respectively; the 1 *H* -7-azaindole, which is the least nucleophilic in the azaindole series, was alkylated to give **1a** in 79% yield. In the case of 1-methyl-1 *H* -7-azaindole, the steric hindrance of the methyl group necessitated the use of the more reactive bromoacetonitrile, to provide quaternary salt **1b** in 61% yield.


**Scheme 2** Synthesis of *N* -cyanomethyl salts **1** – **3**



**Scheme 3** Interaction of salts **1a** , **b** with *o* -hydroxybenzaldehydes


The reaction of 7-(cyanomethyl)-7-azaindolium salt **1a** with salicylic aldehydes under the conditions earlier optimized for the 6-azaindolium salt did not result in the formation of the target polycyclic product of the domino process, but gave coumaryl-substituted 7 *H* -7-azaindoles **4a** – **c** (Scheme 3 ). The structure of compound **4a** was determined by ^1^ H, ^13^ C and ^15^ N NMR spectroscopy using 2D COSY, TOCSY, NOESY, HSQC, edited HSQC, HMBC, long-range HMBC, and ^15^ N HMBC experiments (for details, see the Supporting Information). A possible reason for this reaction pathway is initial deprotonation of N-1 and formation of the anhydrobase of the azaindole. In the absence of a positive charge on N-7 of the azaindole, the reaction loses its driving force, the intermediate **B** is hydrolyzed, and the final cyclization does not occur (Scheme 3 ). In an effort to overcome the problem, 7-(cyanomethyl)-1-methyl-7-azaindolium bromide ( **1b** ) was tested in the analogous reaction. Despite the absence of *N* -H in salt **1b** and the impossibility of forming anhydrobases, the process still followed the undesired pathway, giving compounds **5a** – **d** . It was hypothesized that performing the reaction under water-free conditions might avoid the hydrolysis of the imine intermediate. Therefore, the reaction was carried out in MeOH or DMF under argon atmosphere in the presence of different desiccants, including molecular sieves, and anhydrous magnesium and copper sulfate, and employing anhydrous sodium carbonate and alternative bases, but it still resulted in the formation of hydrolysis product **5a** (Table 1 , entries 1–6). Presumably, the water formed during the condensation is enough for the hydrolysis to proceed. Fortunately, performing the reaction under microwave (MW) irradiation in absolute ethanol, with molecular sieves and anhydrous potassium carbonate, eventually led to the formation of the desired products **6a** – **c** in moderate yields (Scheme 3 ).

**Table:** **Table 1** Screening of Water-Free Conditions for the Reaction of Salicylaldehyde with 7-Azaindolium Salt **1b**

Entry	Conditions	Yield (%), product
1	DBU (1 equiv), MeOH, MS (4 Å), reflux, 3 h	50, **5a**
2	Na _2_ CO _3_ (1 equiv), MeOH, MS (4 Å), reflux, 3 h	32, **5a**
3	Na _2_ CO _3_ (1 equiv), DMF, MS (4 Å), reflux, 3 h	43, **5a**
4	DBU (1 equiv), MgSO _4_ , MeOH, reflux, 3 h	47, **5a**
5	piperidine (1 equiv), CuSO _4_ , MeOH, reflux, 3 h	39, **5a**
6	NH _3_ –MeOH (1 equiv), MeOH, reflux, 3 h	30, **5a**
**7**	** K _2_ CO _3_ (2.2 equiv), anhyd EtOH, MS (4 Å), MW, 150 °C, 7 min **	54, **6a**
8	K _2_ CO _3_ (2.2 equiv), anhyd EtOH, MS (4 Å), MW, 150 °C, 10 min	50, **6a**
9	K _2_ CO _3_ (2.2 equiv), anhyd EtOH, MS (4 Å), MW, 150 °C, 5 min	48, **6a**
10	K _2_ CO _3_ (1.1 equiv), anhyd *i* -PrOH, MS (4 Å), MW, 150 °C, 7 min	11, **6a**
11	K _2_ CO _3_ (0.4 equiv), anhyd EtOH, MS (4 Å), MW, 150 °C, 7 min	38, **6a**
12	DBU (0.2 equiv), anhyd EtOH, MS (4 Å), MW, 150 °C, 7 min	23, **6a**
13	DBU (0.5 equiv), anhyd EtOH, MS (4 Å), MW, 150 °C, 7 min	45, **6a**
14	DBU (0.7 equiv), anhyd EtOH, MS (4 Å), MW, 150 °C, 7 min	40, **6a**
15	DBU (1.1 equiv), anhyd EtOH, MS (4 Å), MW, 150 °C, 7 min	50, **6a**
16	NH _4_ OAc (2.2 equiv), anhyd EtOH, MS (4 Å), MW, 150 °C, 7 min	18, **6a**

The use of DBU (Table 1 , entries 12–15) or ammonium acetate (Table 1 , entry 16) as base, or isopropyl alcohol as solvent (Table 1 , entry 10), was less effective than potassium carbonate in ethanol (Table 1 , entry 7). The modest yields may be associated with the instability of the products **6** under the reaction conditions and the reduction of the reaction times, achieved under microwave conditions, explains the success of the microwave approach.

The optimized conditions were utilized to examine the scope of the reaction of 5-azaindolium salt **2** (Scheme 4 ). Thus, annulated pyrrolopyridines **7a** – **e** were synthesized in 70–87% yield. The preparation of compounds **7** was not as sensitive to the presence of water and the reaction time; for instance, compound **7a** was produced in 64% yield after reflux­ for 8 hours with ammonium acetate in a water–methanol mixture.

The optimized conditions were also employed to examine the reactivity of 4-(cyanomethyl)-4-azaindolium salt **3** in the domino process. Thus, it was shown that the reaction proceeded analogously, giving isomeric chromenoimidazopyrrolopyridines **8a** – **d** (Scheme 5 ).


**Scheme 4** Scope of the reaction of salt **2** with *o* -hydroxybenzaldehydes


The proposed reaction mechanism is as follows: (a) Knoevenagel condensation of the salicylic aldehyde with the quaternary pyrrolopyridine salt followed by (b) nucleophilic cyclization of the phenolate anion, another (c) nucleo­philic cyclization, and (d) aromatization of the imidazole to yield the target product (Scheme 6 ).

In conclusion, we have studied the domino reaction of 4-, 5- and 7-azaindolium salts with substituted salicylic aldehydes. The target chromenoimidazopyrrolopyridines were formed in all cases, providing a reliable route towards analogues of the isogranulatimide marine natural products family.


**Scheme 5** Scope of the reaction of salt **3** with *o* -hydroxybenzaldehydes



**Scheme 6** Plausible mechanism for formation of the chromenoimidazopyrrolopyridine products


## 

Starting azaindoles and aldehydes were purchased from commercial sources (7-azaindole CAS 271-63-6, 5-azaindole CAS 271-34-1, 4-azaindole CAS 272-49-1, chloroacetonitrile CAS 107-14-2, bromoacetonitrile CAS 590-17-0, salicylaldehyde CAS 90-02-8, 5-bromosalicylaldehyde CAS 1761-61-1, 2-hydroxy-5-methoxybenzaldehyde CAS 672-13-9, 2-hydroxy-1-naphthaldehyde CAS 708-06-5, 3-ethoxysalicylaldehyde CAS 492-88-6, 3,5-dichlorosalicylaldehyde CAS 90-60-8) and were used without any additional purification. 1-Methyl-7-azaindole was prepared according to a literature procedure. 11 Solvents were distilled and dried according to standard procedures. ^1^ H and ^13^ C NMR spectra were acquired on 400 or 600 MHz spectrometers and referenced to the residual signals of the solvent. The solvent for NMR samples was DMSO- *d*_6_ or CDCl _3_ with a few drops of TFA. Chemical shifts are reported in parts per million (δ/ppm) and coupling constants in hertz ( *J* /Hz). The peak patterns are indicated as follows: s, singlet; d, doublet; t, triplet; q, quartet; m, multiplet; dd, doublet of doublets; br s, broad singlet. IR spectra were made on an Infralum FT-801; wavelengths are reported in reciprocal centimeters (λ _max_ /cm ^–1^ ). Mass spectra were recorded with a Shimadzu LCMS-8040 triple quadrupole liquid chromatograph–mass spectrometer and MALDI spectra with a Bruker Autoflex Speed instrument in a positive ion reflection mode using solid-state UV laser and EI techniques. Microwave-assisted reactions were carried out in a Monowave 300 reactor (Anton Paar GmbH); the reaction temperature was monitored by an IR sensor. Standard 10-mL G10 reaction vials, sealed with silicone septa, were used for the microwave irradiation experiments. Reaction progress was monitored by TLC and the spots were visualized under UV light (254 or 365 nm). Column chromatography was performed using silica gel (230–400 mesh) and MeOH–CH _2_ Cl _2_ mixtures in different proportions as the mobile phase. Melting points were determined on an SMP-10 apparatus and are uncorrected.

## 7-(Cyanomethyl)-1 *H* -pyrrolo[2,3- *b* ]pyridin-7-ium Chloride (1a)

A solution of 7-azaindole (2 g, 17 mmol) with excess chloroacetonitrile (1.61 mL, 25.5 mmol, 1.5 equiv) in MeCN (5 mL) in a closed vial was placed into a microwave reactor, where it was heated at 140 °C for 30 min. The precipitate was collected by filtration, washed with MeCN (3 × 5 mL) and dried under air to give a gray solid; yield: 2.587 g (79%); mp 203 °С.

IR (KBr): 3123–2710, 1617, 1469, 1346, 1107, 886, 810, 738 cm ^–1^ .

^1^ Н NMR (600 MHz, DМSО- *d*_6_ ): δ = 6.34 (s, 2 Н, СН _2_ ), 7.02 (d, *J* = 3.4 Hz, 1 Н), 7.71–7.73 (m, 1 H), 8.03 (d, *J* = 3.4 Hz, 1 H), 8.85 (d, *J* = 7.7 Hz, 1 H), 8.87 (d, *J* = 6.2 Hz, 1 H).

^13^ С NMR (100 MHz, DМSО- *d*_6_ ): δ = 43.3, 104.1, 113.9, 116.5, 127.1, 130.5, 136.6, 138.9, 139.0.

ESI-MS: *m* / *z* = 158 [M – Cl] ^+^ .

Anal. Calcd for C _9_ H _8_ ClN _3_ (193.63): C, 55.83; H, 4.16; N, 21.70. Found: C, 55.90; H, 4.11; N, 21.78.

## 7-(Cyanomethyl)-1-methyl-1 *H* -pyrrolo[2,3- *b* ]pyridin-7-ium Bromide (1b)

To a solution of 1-methyl-7-azaindole (1.194 g, 9 mmol) in MeCN (4 mL) was added excess bromoacetonitrile (0.940 mL, 13.5 mmol, 1.5 equiv). The reaction mixture was stirred under reflux for 24 h. The precipitate was collected by filtration, washed with MeCN (3 ×) and dried under air to give a gray solid; yield: 1.190 g (52%); mp 194 °С.

IR (KBr): 3118, 3062, 2916, 2260, 1617, 1589, 1505, 1400, 1353, 1244, 1115, 806, 722, 592 cm ^–1^ .

^1^ Н NMR (400 MHz, DМSО- *d*_6_ ): δ = 4.35 (s, 3 H), 6.50 (s, 2 H), 7.04 (d, *J* = 3.3 Hz, 1 H), 7.73 (t, *J* = 7.3 Hz, 1 H), 7.92 (d, *J* = 3.3 Hz, 1 H), 8.80 (d, *J* = 6.5 Hz, 1 H), 8.85 (d, *J* = 7.8 Hz, 1 H).

^13^ С NMR (100 MHz, DМSО- *d*_6_ ): δ = 37.1, 43.7, 103.2, 114.9, 116.5, 128.9, 136.9, 137.9, 138.7, 139.6.

ESI-MS: *m* / *z* = 172 [M – Br] ^+^ .

Anal. Calcd for C _10_ H _10_ BrN _3_ (252.12): C, 47.64; H, 4.00; N, 16.67. Found: C, 47.87; H, 3.93; N, 16.60.

## Compounds 4 and 5; General Procedure

To a solution of salt **1a** or **1b** (0.991 mmol) and the corresponding aldehyde (0.991 mmol) in a MeOH–H _2_ O mixture (1:1, 4 mL) was added NH _4_ OAc (0.991 mmol) at reflux. The reaction mixture was stirred under reflux for 3 h. Upon reaction completion, the solvent was evaporated under reduced pressure and the product was isolated by silica gel column chromatography (MeOH–CH _2_ Cl _2_ , 1:100 to 1:10).

## 3-(7 *H* -Pyrrolo[2,3- *b* ]pyridin-7-yl)-2 *H* -chromen-2-one (4a)

Yellow solid; yield: 0.07 g (27%); mp 142 °С (dec).

IR (KBr): 3098, 1726, 1608, 1272, 1048, 761, 737 cm ^–1^ .

^1^ Н NMR (600 MHz, DМSО- *d*_6_ ): δ = 6.68 (d, *J* = 2.7 Hz, 1 Н, H-3′), 7.12 (t, *J* = 6.9 Hz, 1 Н, H-5′), 7.51 (t, *J* = 7.2 Hz, 1 H, H-6), 7.62 (d, *J* = 8.3 Hz, 1 H, H-8), 7.65 (d, *J* = 2.7 Hz, 1 H, H-2′), 7.80 (t, *J* = 7.6 Hz, 1 H, H-7), 7.88 (dd, *J* = 7.6, 1.4 Hz, 1 H, H-5), 8.21 (d, *J* = 6.2 Hz, 1 H, H-6′), 8.36 (d, *J* = 7.6 Hz, 1 H, H-4′), 8.71 (s, 1 Н, H-4).

^13^ С NMR (100 MHz, DМSО- *d*_6_ ): δ = 101.5 (C-3′), 108.8 (C-5′), 116.6 (C-8), 118.2 (C-8a), 125.4 (C-6), 126.8 (C-3), 129.6 (C-5), 130.4 (C-3a′), 131.4 (C-6′), 132.5 (C-4′), 133.5 (C-7), 141.8 (C-4), 145.0 (C-2′), 148.0 (C-7a′), 153.2 (C-4a), 157.0 (C-2).

EI-MS: *m* / *z* (%) = 263 (20), 262 (100) [M] ^+^ , 261 (24), 235 (12), 234 (67), 206 (24), 205 (42), 145 (96), 131 (11), 118 (18), 103 (24), 102 (11), 90 (14), 89 (43), 76 (12), 63 (17).

Anal. Calcd for C _16_ H _10_ N _2_ O _2_ (262.27): C, 73.27; H, 3.84; N, 10.68. Found: C, 73.44; H, 3.76; N, 10.58.

## 6-Bromo-3-(7 *H* -pyrrolo[2,3- *b* ]pyridin-7-yl)-2 *H* -chromen-2-one (4b)

Yellow solid; yield: 0.119 g (35%); mp 240 °С (dec).

IR (KBr): 3071, 2921–2853, 1726, 1539, 1341, 1268, 1147, 1048, 928, 721 cm ^–1^ .

^1^ Н NMR (600 MHz, DМSО- *d*_6_ ): δ = 6.68 (d, *J* = 2.8 Hz, 1 Н), 7.09–7.13 (m, 1 Н), 7.61 (d, *J* = 8.8 Hz, 1 Н), 7.64 (d, *J* = 2.8 Hz, 1 H), 7.95 (dd, *J* = 8.8, 2.2 Hz, 1 H), 8.13 (d, *J* = 2.2 Hz, 1 H), 8.16 (d, *J* = 6.1 Hz, 1 H), 8.36 (d, *J* = 7.4 Hz, 1 H), 8.63 (s, 1 Н).

^13^ С NMR (100 MHz, CDCl _3_ + TFA): δ = 104.7, 116.4, 118.3, 118.9, 119.2, 124.9, 128.2, 130.8, 132.1, 135.8, 138.4, 139.4, 139.6, 143.5, 152.7, 156.4.

EI-MS: *m* / *z* (%) = 340 (100) [M] ^+^ , 339 (19), 312 (79), 223 (59), 205 (29), 204 (13), 177 (10), 167 (23), 130 (26), 118 (27), 117 (15), 91 (10), 89 (19), 88 (21), 76 (10), 75 (11), 63 (12), 62 (11).

Anal. Calcd for C _16_ H _9_ BrN _2_ O _2_ (341.16): C, 56.33; H, 2.66; N, 8.21. Found: C, 56.44; H, 2.60; N, 8.15.

## 6-Methoxy-3-(7 *H* -pyrrolo[2,3- *b* ]pyridin-7-yl)-2 *H* -chromen-2-one (4c)

Yellow solid; yield: 0.120 g (42%); mp 150 °С (dec).

IR (KBr): 3165, 3104–3046, 2994–2838, 1720, 1580, 1488, 1344, 1269, 1149, 1050, 729 cm ^–1^ .

^1^ Н NMR (600 MHz, DМSО- *d*_6_ ): δ = 3.85 (s, 3 Н, OCH _3_ ), 6.68 (d, *J* = 2.8 Hz, 1 Н), 7.10–7.12 (m, 1 Н), 7.39 (dd, *J* = 9.0, 2.8 Hz, 1 H), 7.42 (d, *J* = 2.8 Hz, 1 H), 7.57 (d, *J* = 9.0 Hz, 1 H), 7.65 (d, *J* = 2.8 Hz, 1 H), 8.20 (d, *J* = 6.8 Hz, 1 H), 8.35 (d, *J* = 6.2 Hz, 1 Н), 8.62 (s, 1 Н).

^13^ С NMR (150 MHz, DМSО- *d*_6_ ): δ = 55.9, 101.4, 108.7, 111.4, 117.7, 118.7, 120.9, 126.9, 130.3, 131.3, 132.3, 141.5, 145.0, 147.5, 148.0, 156.1, 157.0.

EI-MS: *m* / *z* (%) = 293 (14), 292 (70) [M] ^+^ , 265 (19), 264 (100), 249 (19), 221 (22), 193 (19), 192 (15), 176 (12), 175 (97), 146 (10), 131 (10), 119 (36), 118 (14), 103 (11), 76 (11).

Anal. Calcd for C _17_ H _12_ N _2_ O _3_ (292.29): C, 69.86; H, 4.14; N, 9.58. Found: C, 69.99; H, 4.01; N, 9.45.

## 1-Methyl-7-(2-oxo-2 *H* -chromen-3-yl)-1 *H* -pyrrolo[2,3- *b* ]pyridin-7-ium Bromide (5a)

Beige solid; yield: 0.160 g (45%); mp 179 °С (dec).

IR (KBr): 3078, 3005, 1716, 1604, 1258, 1052, 808, 761, 728 cm ^–1^ .

^1^ Н NMR (600 MHz, DМSО- *d*_6_ ): δ = 3.80 (s, 3 H, CH _3_ ), 7.12 (d, *J* = 3.8 Hz, 1 H, H-3′), 7.59 (t, *J* = 7.6 Hz, 1 H, H-6), 7.68 (d, *J* = 8.3 Hz, 1 H, H-8), 7.85 (t, *J* = 7.0 Hz, 1 H, H-5′), 7.90 (m, 1 H, H-7), 7.93 (d, *J* = 3.2 Hz, 1 H, H-2′), 7.96 (d, *J* = 7.6 Hz, 1 H, H-5), 8.73 (d, *J* = 6.4 Hz, 1 H, H-6′), 8.98 (d, *J* = 8.3 Hz, 1 H, H-4′), 9.01 (s, 1 H, H-4).

^13^ С NMR (100 MHz, DМSО- *d*_6_ ): δ = 36.4 (CH _3_ ), 103.2 (C-3′), 116.1 (C-5′), 116.9 (C-8), 117.3 (C-4a), 125.0 (C-3), 125.8 (C-6), 128.2 (C-3a′), 130.6 (C-5), 134.7 (C-7), 136.5 (C-2′), 137.6 (C-7a′), 139.6 (C-6′), 140.1 (C-4′), 144.4 (C-4), 153.6 (C-8a), 157.3 (C-2).

MS (MALDI): *m* / *z* = 277 [M – Br] ^+^ .

Anal. Calcd for C _17_ H _13_ BrN _2_ O _2_ (357.21): C, 57.16; H, 3.67; N, 7.84. Found: C, 57.01; H, 3.52; N, 7.77.

## 7-(6-Bromo-2-oxo-2 *H* -chromen-3-yl)-1-methyl-1 *H* -pyrrolo[2,3- *b* ]pyridin-7-ium Bromide (5b)

Orange solid; yield: 0.095 g (22%); mp 125 °С (dec).

IR (KBr): 3091, 2922–2853, 1742, 1598, 1449–1409, 1354, 1253, 1160–1046, 818, 723 cm ^–1^ .

^1^ Н NMR (600 MHz, DМSО- *d*_6_ ): δ = 3.80 (s, 3 H, CH _3_ ), 7.12 (d, *J* = 3.4 Hz, 1 H), 7.68 (d, *J* = 8.8 Hz, 1 H), 7.83 (dd, *J* = 7.8, 6.7 Hz, 1 H), 7.92 (d, *J* = 3.4 Hz, 1 H), 8.04 (dd, *J* = 8.8, 2.5 Hz, 1 H), 8.24 (d, *J* = 2.5 Hz, 1 H), 8.68 (dd, *J* = 6.7, 1.0 Hz, 1 H), 8.92 (s, 1 H), 8.97 (dd, *J* = 7.8, 1.0 Hz, 1 H).

^13^ С NMR (100 MHz, DМSО- *d*_6_ ): δ = 36.6, 103.5, 116.3, 117.4, 119.3, 119.3, 126.0, 128.5, 132.5, 136.7, 137.1, 137.7, 139.5, 140.4, 143.3, 152.9, 157.1.

MS (MALDI): *m* / *z* = 355 [M – Br] ^+^ .

Anal. Calcd for C _17_ H _12_ Br _2_ N _2_ O _2_ (436.10): C, 46.82; H, 2.77; N, 6.42. Found: C, 46.98; H, 2.72; N, 6.33.

## 1-Methyl-7-(3-oxo-3 *H* -benzo[ *f* ]chromen-2-yl)-1 *H* -pyrrolo[2,3- *b* ]pyridin-7-ium Bromide (5c)

Dark gray solid; yield: 0.130 g (32%); mp 176 °С (dec).

IR (KBr): 3210, 3095–2843, 1660, 1619–1580, 1493, 1451–1413, 1357, 1261–1222, 1048, 890, 832, 729, 588 cm ^–1^ .

^1^ Н NMR (600 MHz, DМSО- *d*_6_ ): δ = 3.82 (s, 3 H, CH _3_ ), 7.16 (d, *J* = 3.3 Hz, 1 H), 7.75 (d, *J* = 7.5 Hz, 1 H), 7.84 (d, *J* = 8.8 Hz, 2 H), 7.90 (t, *J* = 7.0 Hz, 1 H), 7.95 (d, *J* = 3.3 Hz, 1 H), 8.20 (d, *J* = 8.1 Hz, 1 H), 8.49 (d, *J* = 8.8 Hz, 2 H), 8.81 (d, *J* = 5.1 Hz, 1 H), 9.02 (d, *J* = 7.7 Hz, 1 H), 9.91 (s, 1 H).

^13^ С NMR (100 MHz, DМSО- *d*_6_ ): δ = 36.6, 103.3, 104.8, 112.1, 116.2, 116.7, 122.5, 124.2, 126.9, 128.2, 129.1 (2 C), 130.2, 136.3, 136.6, 137.6, 140.0, 140.2, 141.2, 154.3, 157.3.

ESI-MS: *m* / *z* = 327 [M – Br] ^+^ .

Anal. Calcd for C _21_ H _15_ BrN _2_ O _2_ (407.27): C, 61.93; H, 3.71; N, 6.88. Found: C, 62.09; H, 3.60; N, 6.69.

## 7-(6,8-Dichloro-2-oxo-2 *H* -chromen-3-yl)-1-methyl-1 *H* -pyrrolo[2,3- *b* ]pyridin-7-ium Bromide (5d)

Yellow solid; yield: 0.197 g (47%); mp 118 °С (dec).

IR (KBr): 3092–3020, 2954–2767, 1737, 1619, 1527, 1447–1410, 1357, 1232, 1048, 808, 727 cm ^–1^ .

^1^ Н NMR (600 MHz, DМSО- *d*_6_ ): δ = 4.04 (s, 3 H, CH _3_ ), 7.00 (d, *J* = 3.4 Hz, 1 H), 7.45 (d, *J* = 2.8 Hz, 1 H), 7.50 (s, 1 H), 7.60 (d, *J* = 2.8 Hz, 1 H), 7.66 (dd, *J* = 7.6, 6.2 Hz, 1 H), 7.84 (d, *J* = 3.4 Hz, 1 H), 8.66 (d, *J* = 5.5 Hz, 1 H), 8.78 (d, *J* = 7.6 Hz, 1 H).

^13^ C NMR (100 MHz, DМSО- *d*_6_ ): δ = 36.6, 102.6, 115.4, 116.3, 121.3, 124.2, 125.7, 127.6, 130.4, 131.2, 134.9, 136.0, 136.8, 137.7, 139.9, 153.9, 163.6.

ESI-MS: *m* / *z* = 345 [M – Br] ^+^ .

Anal. Calcd for C _17_ H _11_ BrCl _2_ N _2_ O _2_ (426.09): C, 47.92; H, 2.60; N, 6.57. Found: C, 48.12; H, 2.70; N, 6.50.

## Compounds 6; General Procedure

A solution of salt **1b** (0.13 g, 0.516 mmol) and the corresponding aldehyde (0.512 mmol) in anhyd EtOH (4 mL) with K _2_ CO _3_ (0.173 g, 2.2 equiv) in a closed vial was placed into a microwave reactor, where it was heated at 150 °C for 7 min. Upon reaction completion, the mixture was diluted with H _2_ O (8 mL) and EtOH (3 mL), and the formed precipitate was collected by filtration, washed with an EtOH–H _2_ O mixture (7:8) (3 × 3 mL) and dried under air.

## 1-Methyl-1,12-dihydrochromeno[2′,3′:4,5]imidazo[1,2- *a* ]pyrrolo[3,2- *e* ]pyridine (6a)

Brown solid; yield: 0.076 g (54%); mp 148 °С.

IR (KBr): 3500–2841, 1625, 1561, 1490, 1454–1422, 1368, 1285, 1249, 1224, 799, 755, 707, 656 cm ^–1^ .

^1^ Н NMR (600 MHz, DМSО- *d*_6_ ): δ = 4.14 (s, 3 H, CH _3_ ), 4.68 (s, 2 H, CH _2_ ), 6.54 (d, *J* = 2.6 Hz, 1 H), 7.14–7.19 (m, 4 H), 7.30 (t, *J* = 7.3 Hz, 1 H), 7.45 (d, *J* = 7.0 Hz, 1 H), 7.55 (d, *J* = 9.2 Hz, 1 H).

^13^ С NMR (100 MHz, DМSО- *d*_6_ ): δ = 28.2, 38.2, 96.3, 102.3, 107.7, 113.6, 116.8, 119.1, 120.5, 123.2, 125.4, 127.9, 129.2, 130.5, 140.7, 149.9, 150.2.

ESI-MS: *m* / *z* = 276 [M + Н] ^+^ .

Anal. Calcd for C _17_ H _13_ N _3_ O (275.31): C, 74.17; H, 4.76; N, 15.26. Found: C, 74.06; H, 4.83; N, 15.20.

## 10-Methoxy-1-methyl-1,12-dihydrochromeno[2′,3′:4,5]imidazo[1,2- *a* ]pyrrolo[3,2- *e* ]pyridine (6b)

Brown solid; yield: 0.074 g (47%); mp 188 °С.

IR (KBr): 3655–2837, 1709, 1624–1568, 1495–1369, 1284–1210, 1035, 794, 720, 655 cm ^–1^ .

^1^ Н NMR (600 MHz, DМSО- *d*_6_ ): δ = 3.77 (s, 3 H, CH _3_ ), 4.14 (s, 3 H, CH _3_ ), 4.64 (s, 2 H, CH _2_ ), 6.54 (d, *J* = 3.3 Hz, 1 H), 6.87 (dd, *J* = 8.8, 2.8 Hz, 1 H), 7.03 (d, *J* = 2.8 Hz, 1 H), 7.11–7.16 (m, 3 H), 7.54 (d, *J* = 8.8 Hz, 1 H).

^13^ С NMR (100 MHz, DМSО- *d*_6_ ): δ = 28.6, 38.2, 55.4, 95.9, 102.2, 107.7, 113.6, 114.1, 114.2, 117.5, 119.7, 120.4, 125.4, 129.2, 140.7, 144.1, 150.2, 154.8.

ESI-MS: *m* / *z* = 306 [M + Н] ^+^ .

Anal. Calcd for C _18_ H _15_ N _3_ O _2_ (305.34): C, 70.81; H, 4.95; N, 13.76. Found: C, 70.59; H, 4.99; N, 13.69.

## 8-Ethoxy-1-methyl-1,12-dihydrochromeno[2′,3′:4,5]imidazo[1,2- *a* ]pyrrolo[3,2- *e* ]pyridine (6c)

Brown solid; yield: 0.058 g (36%); mp 149 °С.

IR (KBr): 3624–2845, 1708–1567, 1500–1423, 1270, 1209, 1115–1000, 793, 760, 721, 655 cm ^–1^ .

^1^ Н NMR (600 MHz, DМSО- *d*_6_ ): δ = 1.42 (t, *J* = 7.0 Hz, 3 H, CH _3_ ), 4.10–4.13 (m, 5 H, OC *H _2_* CH _3_ , NCH _3_ ), 4.68 (s, 2 H, CH _2_ ), 6.54 (d, *J* = 2.9 Hz, 1 H, H-3), 6.97–6.99 (m, 2 H, H-11, H-9), 7.05 (t, *J* = 7.7 Hz, 1 H, H-10), 7.13 (d, *J* = 2.9 Hz, 1 H, H-2), 7.16 (d, *J* = 9.1 Hz, 1 H, H-4), 7.56 (d, *J* = 9.1 Hz, 1 H, H-4).

^13^ С NMR (100 MHz, DМSО- *d*_6_ ): δ = 14.7 (CH _2_ *C* H _3_ ), 28.4 (CH _2_ ), 38.2 (NCH _3_ ), 64.0 ( *C* H _2_ CH _3_ ), 96.2 (C-12a), 102.2 (C-3), 107.7 (C-5), 111.5 (C-11), 113.6 (C-3a), 119.8 (C-8a), 120.5 (C-4), 121.6 (C-9), 122.8 (C-10), 125.4 (C-2), 129.2 (C-13a), 140.1 (C-11a), 140.7 (C-6a), 147.2 (C-8), 149.9 (C-7a).

ESI-MS: *m* / *z* = 320 [M + Н] ^+^ .

Anal. Calcd for C _19_ H _17_ N _3_ O _2_ (319.36): C, 71.46; H, 5.37; N, 13.16. Found: C, 71.34; H, 5.46; N, 13.05.

## 5-(Сyanomethyl)-1 *H* -pyrrolo[3,2- *c* ]pyridin-5-ium Chloride (2)

To a solution of 5-azaindole (1 g, 8.5 mmol) in MeCN (5 mL) was added chloroacetonitrile (0.8 mL, 12.75 mmol). The reaction mixture was stirred under reflux for 6 h. The precipitate was collected by filtration, washed with MeCN (3 × 5 mL) and dried under air to give a gray solid; yield: 1.31 g (80%); mp 212–214 °С.

IR (KBr): 3203–2541, 1893, 1778, 1636, 1602, 1523, 1484, 1417, 1361, 1334, 1276, 1240, 1139, 924, 816, 729 cm ^–1^ .

^1^ Н NMR (600 MHz, DМSО- *d*_6_ ): δ = 6.10 (s, 2 H), 7.11 (d, *J* = 3.3 Hz, 1 H), 8.03 (d, *J* = 3.3 Hz, 1 H), 8.13 (d, *J* = 7.0 Hz, 1 H), 8.67 (d, *J* = 7.0 Hz, 1 H), 9.60 (s, 1 H), 13.62 (br s, 1 H).

^13^ С NMR (150 MHz, DМSО- *d*_6_ ): δ = 46.2, 104.6, 110.3, 115.3, 124.8, 133.8, 134.3, 139.7, 141.3.

ESI-MS: *m* / *z* = 158 [M – Cl] ^+^ .

Anal. Calcd for C _9_ H _8_ ClN _3_ (193.63): C, 55.83; H, 4.16; N, 21.70. Found: C, 55.97; H, 4.08; N, 21.80.

## 4-(Cyanomethyl)-1 *H* -pyrrolo[3,2- *b* ]pyridin-4-ium Chloride (3)

To a solution of 4-azaindole (1 g, 8.5 mmol) in MeCN (5 mL) was added chloroacetonitrile (0.8 mL, 12.75 mmol). The reaction mixture was stirred under reflux for 6 h. The precipitate was collected by filtration, washed with MeCN (3 ×) and dried under air to give a beige solid; yield: 1.358 g (83%); mp 226–228 °С.

IR (KBr): 3013–2573, 1637, 1583, 1462, 1384, 1342, 1286, 1237, 1168, 1131, 900, 822, 796, 764, 598 cm ^–1^ .

^1^ Н NMR (600 MHz, DМSО- *d*_6_ ): δ = 6.34 (s, 2 H), 7.21 (d, *J* = 3.1 Hz, 1 H), 7.78 (dd, *J* = 6.2, 1.5 Hz, 1 H), 8.46 (d, *J* = 3.1 Hz, 1 H), 8.74 (d, *J* = 7.6 Hz, 1 H), 9.00 (d, *J* = 6.2 Hz, 1 H).

^13^ С NMR (150 MHz, DМSО- *d*_6_ ): δ = 43.9, 96.2, 114.3, 117.2, 129.4, 132.7, 137.0, 138.3, 138.4.

ESI-MS: *m* / *z* = 158 [M – Cl] ^+^ .

Anal. Calcd for C _9_ H _8_ ClN _3_ (193.63): C, 55.83; H, 4.16; N, 21.70. Found: C, 55.95; H, 4.06; N, 21.75.

## Compounds 7 and 8; General Procedure

A solution of salt **2** or **3** (0.110 g, 0.57 mmol) and the corresponding aldehyde (0.512 mmol) in anhyd EtOH (4 mL) with K _2_ CO _3_ (0.173 g, 2.2 equiv) in a closed vial was placed into a microwave reactor, where it was heated at 150 °C for 7 min. Upon reaction completion, the mixture was diluted with H _2_ O (10 mL), and the formed precipitate was collected by filtration, washed with EtOH (2 × 3 mL) and with H _2_ O (1 × 3 mL), and dried under air.

## 3,7-Dihydrochromeno[2′,3′:4,5]imidazo[1,2- *a* ]pyrrolo[3,2- *c* ]pyridine (7a)

Beige solid; yield: 0.104 g (79%); mp 276–278 °С (dec).

IR (KBr): 3157–2695, 1778, 1722, 1649, 1427, 1392, 1369, 1327, 1212, 881, 750, 733 cm ^–1^ .

^1^ Н NMR (600 MHz, DМSО- *d*_6_ ): δ = 4.30 (s, 2 H, CH _2_ ), 6.71 (br s, 1 H, H-1), 7.13–7.19 (m, 3 H, H-10, H-4, H-11), 7.30 (t, *J* = 7.6 Hz, 1 H, H-9), 7.35 (t, *J* = 2.8 Hz, 1 H, H-2), 7.39 (d, *J* = 7.6 Hz, 1 H, H-8), 7.88 (d, *J* = 7.6 Hz, 1 H, H-5), 11.63 (s, 1 H, NH).

^13^ С NMR (100 MHz, DМSО- *d*_6_ ): δ = 27.7 (CH _2_ ), 96.1 (С-3a), 100.1 (C-1), 101.1 (C-4), 112.9 (C-13a), 117.3 (C-11a), 117.9 (C-11), 118.6 (C-5), 123.1 (C-10), 123.7 (C-2), 127.8 (C-9), 130.5 (C-8, C-13b), 136.7 (C-6a), 149.1 (C-12a), 151.4 (C-7a).

ESI-MS: *m* / *z* = 262 [M + Н] ^+^ .

Anal. Calcd for C _16_ H _11_ N _3_ O (261.28): C, 73.55; H, 4.24; N, 16.08. Found: C, 73.45; H, 4.34; N, 15.96.

## 9-Bromo-3,7-dihydrochromeno[2′,3′:4,5]imidazo[1,2- *a* ]pyrrolo[3,2- *c* ]pyridine (7b)

Light brown solid; yield: 0.13 g (75%); mp >300 °C.

IR (KBr): 3157–2721, 1649, 1472, 1427, 1393, 1320, 1113, 874, 820, 732 cm ^–1^ .

^1^ Н NMR (600 MHz, DМSО- *d*_6_ ): δ = 4.31 (s, 2 H), 6.70 (s, 1 H), 7.17 (m, 2 Н), 7.35 (s, 1 H), 7.47 (dd, *J* = 8.3, 1.7 Hz, 1 H), 7.60 (s, 1 H), 7.85 (d, *J* = 7.4 Hz, 1 H), 11.65 (s, 1 H).

^13^ С NMR (100 MHz, DМSО- *d*_6_ ): δ = 22.7, 95.8, 100.2, 101.4, 112.9, 114.6, 118.1, 119.6, 121.5, 123.9, 130.6, 133.0, 136.8, 148.9, 150.6, 155.9.

ESI-MS: *m* / *z* = 340 [M + H] ^+^ .

Anal. Calcd for C _16_ H _10_ BrN _3_ O (340.18): C, 56.49; H, 2.96; N, 12.35. Found: C, 56.36; H, 2.99; N, 12.30.

## 9-Methoxy-3,7-dihydrochromeno[2′,3′:4,5]imidazo[1,2- *a* ]pyrrolo[3,2- *c* ]pyridine (7c)

Light brown solid; yield: 0.130 g (87%); mp >300 °С.

IR (KBr): 3155–2834, 1651, 1491, 1434, 1368, 1197, 1040, 802, 730 cm ^–1^ .

^1^ Н NMR (600 MHz, DМSО- *d*_6_ ): δ = 3.77 (s, 3 Н), 4.26 (s, 2 H), 6.70 (s, 1 H), 6.89 (d, *J* = 8.3 Hz, 1 H), 6.93 (s, 1 H), 7.14 (m, 2 Н), 7.34 (s, 1 H), 7.85 (d, *J* = 7.4 Hz, 1 H), 11.62 (s, 1 H).

^13^ С NMR (100 MHz, DМSО- *d*_6_ , 45 °C): δ = 23.8, 55.1, 95.4, 99.8, 100.7, 112.6, 113.6, 114.3, 117.7, 117.8, 119.0, 123.4, 130.3, 136.4, 145.1, 149.2, 154.6.

ESI-MS: *m* / *z* = 292 [M + H] ^+^ .

Anal. Calcd for C _17_ H _13_ N _3_ O _2_ (291.31): С, 70.09; H, 4.50; N, 14.42. Found: С, 69.95; H, 4.67; N, 14.32.

## 3,7-Dihydrobenzo[5′,6′]chromeno[2′,3′:4,5]imidazo[1,2- *a* ]pyrrolo[3,2- *c* ]pyridine (7d)

Gray solid; yield: 0.134 g (84%); mp 293–296 °С (dec).

IR (KBr): 3209, 3116, 3049, 2981, 2821, 1661, 1596, 1583, 1518, 1427, 1390, 1310, 1224, 741 cm ^–1^ .

^1^ Н NMR (600 MHz, DМSО- *d*_6_ ): δ = 4.58 (s, 2 H), 6.75 (s, 1 H), 7.24 (d, *J* = 7.4 Hz, 1 H), 7.38 (s, 1 Н), 7.44 (d, *J* = 8.3 Hz, 1 H), 7.54 (d, *J* = 7.4 Hz, 1 H), 7.70 (d, *J* = 7.4 Hz, 1 H), 7.94 (d, *J* = 9.1 Hz, 1 H), 7.98 (d, *J* = 7.4 Hz, 1 H), 8.04 (d, *J* = 8.3 Hz, 1 H), 8.08 (d, *J* = 7.4 Hz, 1 H), 11.69 (s, 1 H).

^13^ С NMR (100 MHz, DМSО- *d*_6_ ): δ = 21.0, 97.0, 100.2, 101.3, 111.2, 113.0, 118.2, 118.5, 123.0, 123.8, 124.6, 127.0, 128.2, 128.6, 129.9, 130.6, 132.3, 136.8, 148.7, 148.9.

ESI-MS: *m* / *z* = 312 [M + H] ^+^ .

Anal. Calcd for C _20_ H _13_ N _3_ O (311.34): C, 77.16; H, 4.21; N, 13.50. Found: C, 77.03; H, 4.30; N, 13.39.

## 11-Ethoxy-3,7-dihydrochromeno[2′,3′:4,5]imidazo[1,2- *a* ]pyrrolo[3,2- *c* ]pyridine (7e)

Beige solid; yield: 0.109 g (70%); mp 299–304 °С (dec).

IR (KBr): 3160–2837, 1655, 1574, 1470, 1422, 1393, 1326, 1262, 1199, 1082, 877, 753, 711 cm ^–1^ .

^1^ Н NMR (600 MHz, DМSО- *d*_6_ ): δ = 1.41 (br s, 3 Н), 4.09 (m, 2 H), 4.28 (s, 2 H), 6.68 (s, 1 H), 6.91 (d, *J* = 6.6 Hz, 1 H), 6.96 (d, *J* = 6.6 Hz, 1 Н), 7.02 (d, *J* = 6.6 Hz, 1 H), 7.16 (d, *J* = 5.8 Hz, 1 H), 7.35 (s, 1 H), 7.86 (d, *J* = 6.6 Hz, 1 H), 11.71 (s, 1 H).

Due to the poor solubility of **7e** , the ^13^ C NMR spectrum could not be recorded. The use of a CDCl _3_ –TFA mixture as solvent led to compound degradation.

ESI-MS: *m* / *z* = 306 [M + H] ^+^ .

Anal. Calcd for C _18_ H _15_ N _3_ O _2_ (305.34): C, 70.81; H, 4.95; N, 13.76. Found: C, 70.75; H, 4.99; N, 13.70.

## 3,12-Dihydrochromeno[2′,3′:4,5]imidazo[1,2- *a* ]pyrrolo[2,3- *e* ]pyridine (8a)

Beige solid; yield: 0.077 g (57%); mp 280–283 °С (dec).

IR (KBr): 3186–2723, 1643, 1569, 1429, 1207, 888, 751 cm ^–1^ .

^1^ Н NMR (600 MHz, DМSО- *d*_6_ ): δ = 4.69 (s, 2 H, CH _2_ ), 6.83 (br s, 1 H, H-1), 7.13–7.15 (m, 2 H, H-9, H-5), 7.17 (d, *J* = 8.1 Hz, 1 H, H-4), 7.30 (t, *J* = 7.4 Hz, 1 H, H-10), 7.41–7.43 (m, 2 Н, H-2, H-11), 7.46 (d, *J* = 9.2 Hz, 1 H, H-8), 11.67 (br s, 1 H, NH).

^13^ С NMR (100 MHz, DМSО- *d*_6_ ): δ = 24.2 (CH _2_ ), 94.2 (C-1), 97.9 (C-12a), 108.6 (C-5), 113.2 (C-8), 117.4 (C-4), 118.7 (C-7a), 122.4 (C-13a), 123.3 (C-9), 123.5 (C-2), 124.3 (C-3a), 128.0 (C-10), 130.7 (C-11), 137.4 (C-5a), 150.0 (C-6a), 151.5 (C-11a).

ESI-MS: *m* / *z* = 262 [M + H] ^+^ .

Anal. Calcd for C _16_ H _11_ N _3_ O (261.28): C, 73.55; H, 4.24; N, 16.08. Found: C, 73.42; H, 4.31; N, 15.99.

## 10-Bromo-3,12-dihydrochromeno[2′,3′:4,5]imidazo[1,2- *a* ]pyrrolo[2,3- *e* ]pyridine (8b)

Beige solid; yield: 0.103 g (56%); mp >300 °С.

IR (KBr): 3188–2733, 1635, 1470, 1429, 1247, 1210, 789, 696 cm ^–1^ .

^1^ Н NMR (600 MHz, DМSО- *d*_6_ ): δ = 4.70 (s, 2 H), 6.78 (s, 1 H), 7.13–7.17 (m, 2 H), 7.43–7.47 (m, 3 H), 7.62 (s, 1 H), 11.69 (br s, 1 H).

^13^ С NMR (100 MHz, DМSО- *d*_6_ ): δ = 23.9, 94.1, 97.4, 108.5, 113.3, 114.6, 119.6, 121.5, 122.3, 123.5, 124.1, 130.7, 132.9, 137.3, 149.6, 150.7.

ESI-MS: *m* / *z* = 340 [M + H] ^+^ .

Anal. Calcd for C _16_ H _10_ BrN _3_ O (340.18): C, 56.49; H, 2.96; N, 12.35. Found: C, 56.40; H, 3.01; N, 12.29.

## 10-Methoxy-3,12-dihydrochromeno[2′,3′:4,5]imidazo[1,2- *a* ]pyrrolo[2,3- *e* ]pyridine (8c)

Beige solid; yield: 0.070 g (47%); mp 273–276 °С (dec).

IR (KBr): 3188–2639, 1638, 1494–1430, 1429, 1199, 883, 712 cm ^–1^ .

^1^ Н NMR (600 MHz, DМSО- *d*_6_ ): δ = 3.77 (s, 3 Н), 4.67 (s, 2 H), 6.81 (d, *J* = 2.8 Hz, 1 H), 6.89 (dd, *J* = 8.9, 3.0 Hz, 1 H), 6.98 (d, *J* = 2.8 Hz, 1 H), 7.13 (m, 2 H), 7.41 (d, *J* = 2.5 Hz, 1 H), 7.44 (d, *J* = 9.1 Hz, 1 H), 11.67 (br s, 1 H).

^13^ С NMR (100 MHz, DМSО- *d*_6_ ): δ = 24.5, 55.4, 94.1, 97.5, 108.4, 113.1, 114.1, 114.5, 118.1, 119.3, 122.2, 123.4, 124.2, 137.2, 145.2, 150.1, 154.9.

ESI-MS: *m* / *z* = 292 [M + H] ^+^ .

Anal. Calcd for C _17_ H _13_ N _3_ O _2_ (291.31): С, 70.09; H, 4.50; N, 14.42. Found: С, 69.98; H, 4.58; N, 14.38.

## 3,14-Dihydrobenzo[5′,6′]chromeno[2′,3′:4,5]imidazo[1,2- *a* ]pyrrolo[2,3- *e* ]pyridine (8d)

Beige solid; yield: 0.070 g (44%); mp 273–276 °С (dec).

IR (KBr): 3432–2713, 1643, 1576, 1428–1396, 1311, 1230, 802, 707 cm ^–1^ .

^1^ Н NMR (600 MHz, DМSО- *d*_6_ ): δ = 4.56 (s, 2 H), 6.75 (d, *J* = 2.5 Hz, 1 H), 7.24 (d, *J* = 7.0 Hz, 1 H), 7.37 (d, *J* = 2.5 Hz, 1 H), 7.44 (d, *J* = 9.1 Hz, 1 H), 7.55 (d, *J* = 7.4 Hz, 1 H), 7.70 (t, *J* = 7.4 Hz, 1 H), 7.95 (d, *J* = 9.1 Hz, 1 H), 7.99 (d, *J* = 8.2 Hz, 1 H), 8.05 (d, *J* = 8.2 Hz, 1 H), 8.08 (d, *J* = 7.0 Hz, 1 H), 11.68 (br s, 1 H).

^13^ С NMR (100 MHz, DМSО- *d*_6_ ): δ = 20.9, 96.9, 100.2, 101.3, 111.2, 112.9, 118.2, 118.5, 123.0, 123.8, 124.6, 126.9, 128.2, 128.6, 129.9, 130.7, 132.3, 136.9, 148.7, 148.9.

ESI-MS: *m* / *z* = 312 [M + H] ^+^ .

Anal. Calcd for C _20_ H _13_ N _3_ O (311.34): C, 77.16; H, 4.21; N, 13.50. Found: C, 77.09; H, 4.25; N, 13.42.
