# Supplementary material for: Synthesis of Chromenoimidazoles, Annulated with an Azaindole Moiety, through a Base-Promoted Domino Reaction of Cyano­methyl Quaternary Salts
Source: Synthesis (Stuttg). 2017 Apr 4;49(12):2753–60. doi: 10.1055/s-0036-1589496 (PMC6193216; doi:10.1055/s-0036-1589496)
Supplement: Supplementary file 1 — Supporting Information [file sup_ss-2017-t1862-op_10-1055_s-0036-1589496.pdf]

Synthesis

Reviews and Full Papers in Chemical Synthesis

Supporting Information

for DOI: 10.1055/s-0036-1589496

© Georg Thieme Verlag KG Stuttgart · New York 2017

Thieme

Synthesis of chromenoimidazoles, annulated with an azaindole moiety, through a base-promoted domino reaction of cyanomethyl quaternary salts.

Leonid G. Voskressensky[†]*, Olga A. Storozhenko[†], Alexey A. Festa[†], Roman A. Novikov[‡], Alexey V. Varlamov[†]

[†] *Peoples' Friendship University of Russia (RUDN University), Moscow, Russian Federation, Miklukho-Maklaya st., 6*

[‡]*Engelhardt Institute of Molecular Biology, Russian Academy of Sciences, Moscow, Russian Federation, Vavilova 32*

Contents

1. ^{1}H and ^{13}C NMR Spectra p. 2 – 45.
2. 2D NMR Spectra of compounds **4 a, 5a, 6c, 7a, 9a** p. 46 – 69.

1a

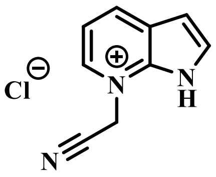

Cl
N
N
H
N

^{1}H

DMSO-d_6

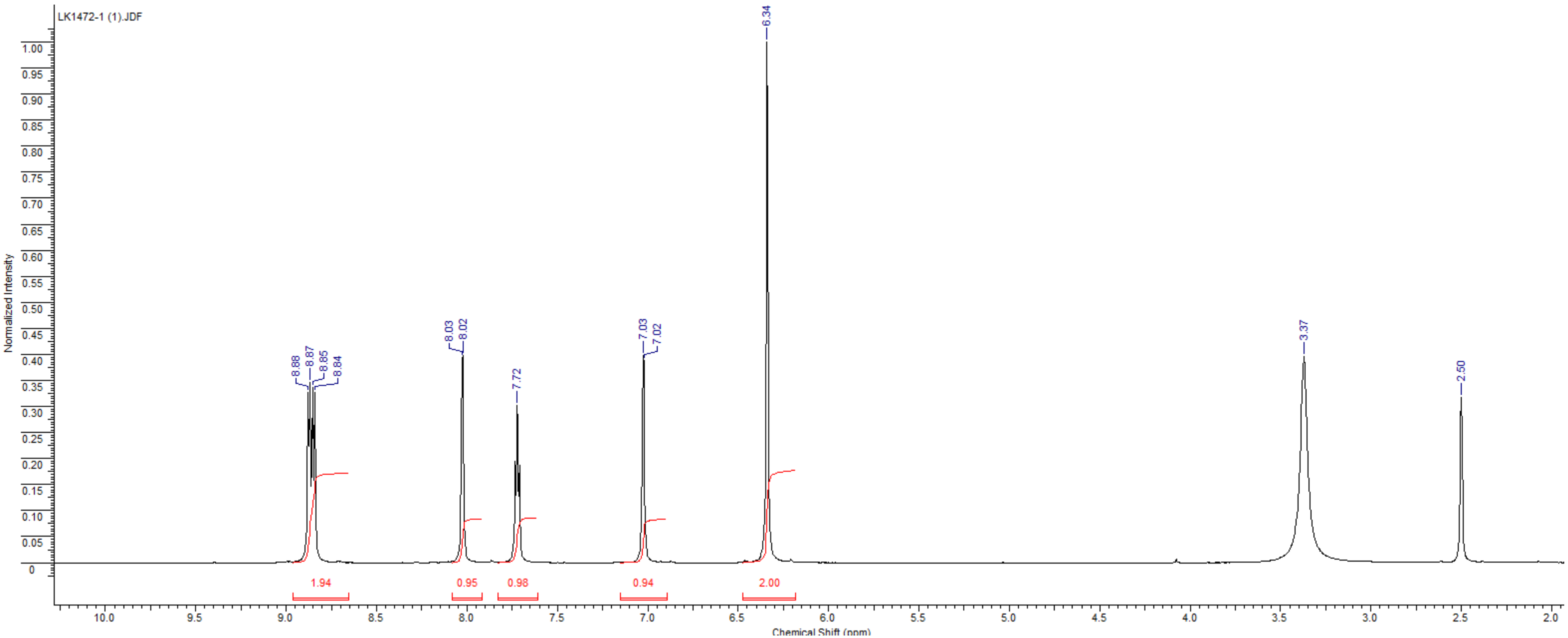

LK1472-1 (1).JDF
Normalized Intensity
8.88
8.87
8.85
8.84
8.03
8.02
7.72
7.03
7.02
6.34
3.37
2.50
1.94
0.95
0.98
0.94
2.00
Chemical Shift (ppm)

1a

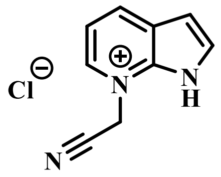

^{13}C

DMSO-d_6

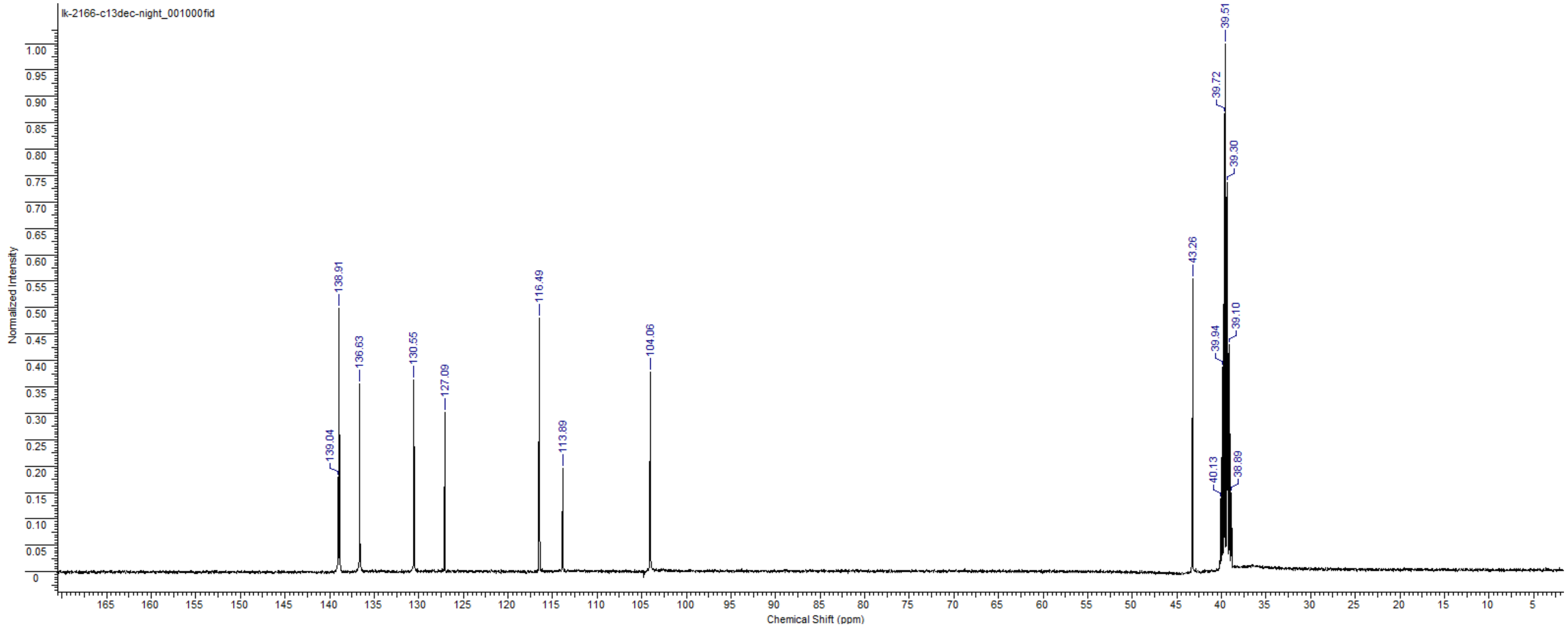

4a

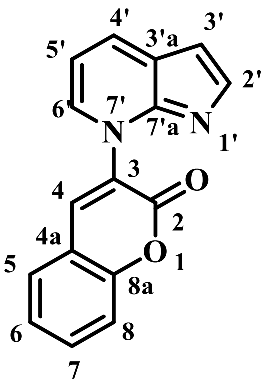
4'
3'a
3'
5'
2'
6'
7'
7'a
N
N
1'
3
O
4
2
4a
O
1
5
8a
6
8
7

^{1}H

DMSO-d_6

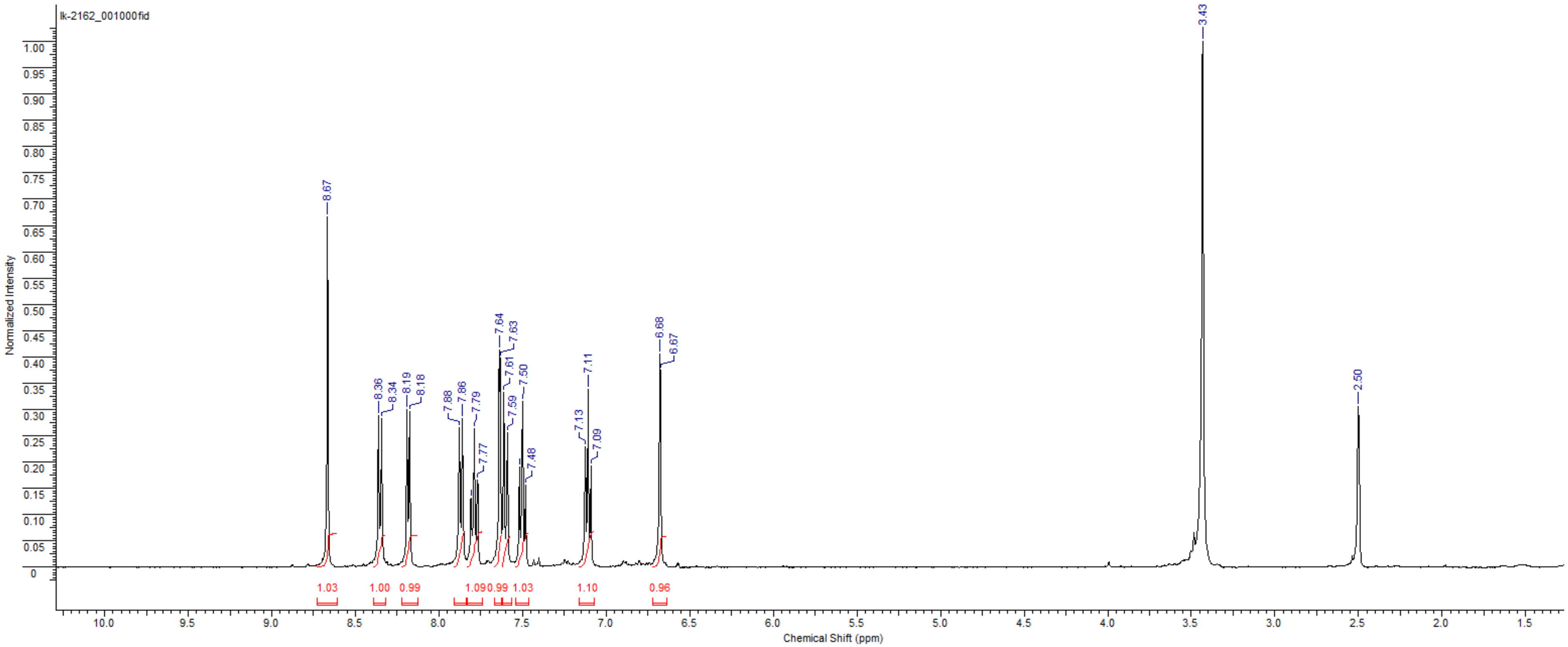
lk-2162_001000fid
Normalized Intensity
8.67
8.36
8.34
8.19
8.18
7.88
7.86
7.79
7.77
7.64
7.63
7.61
7.59
7.50
7.48
7.13
7.11
7.09
6.68
6.67
3.43
2.50
1.03
1.00
0.99
1.09
0.99
1.03
1.10
0.96
Chemical Shift (ppm)

4a

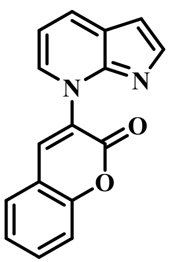

^{13}C

DMSO-d_6

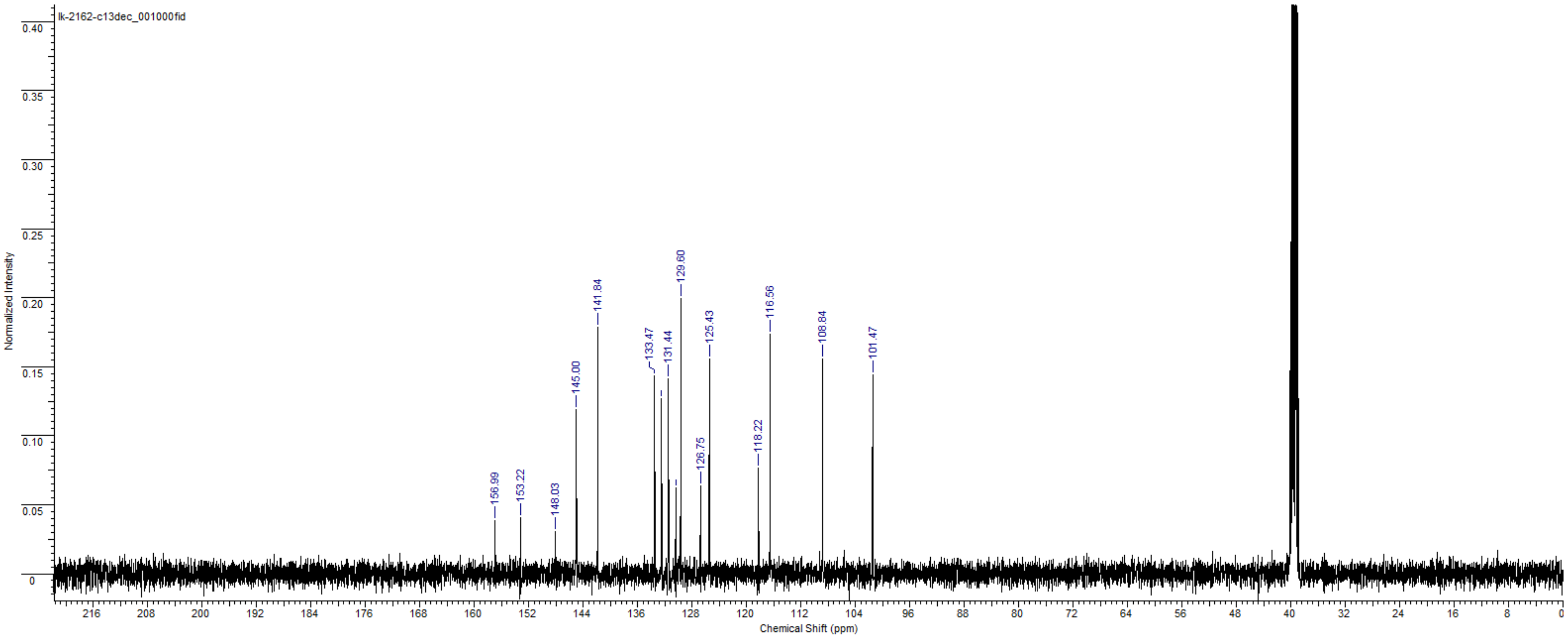

4b

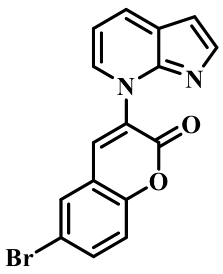

N
N
O
O
Br

^{1}H

DMSO-d_6

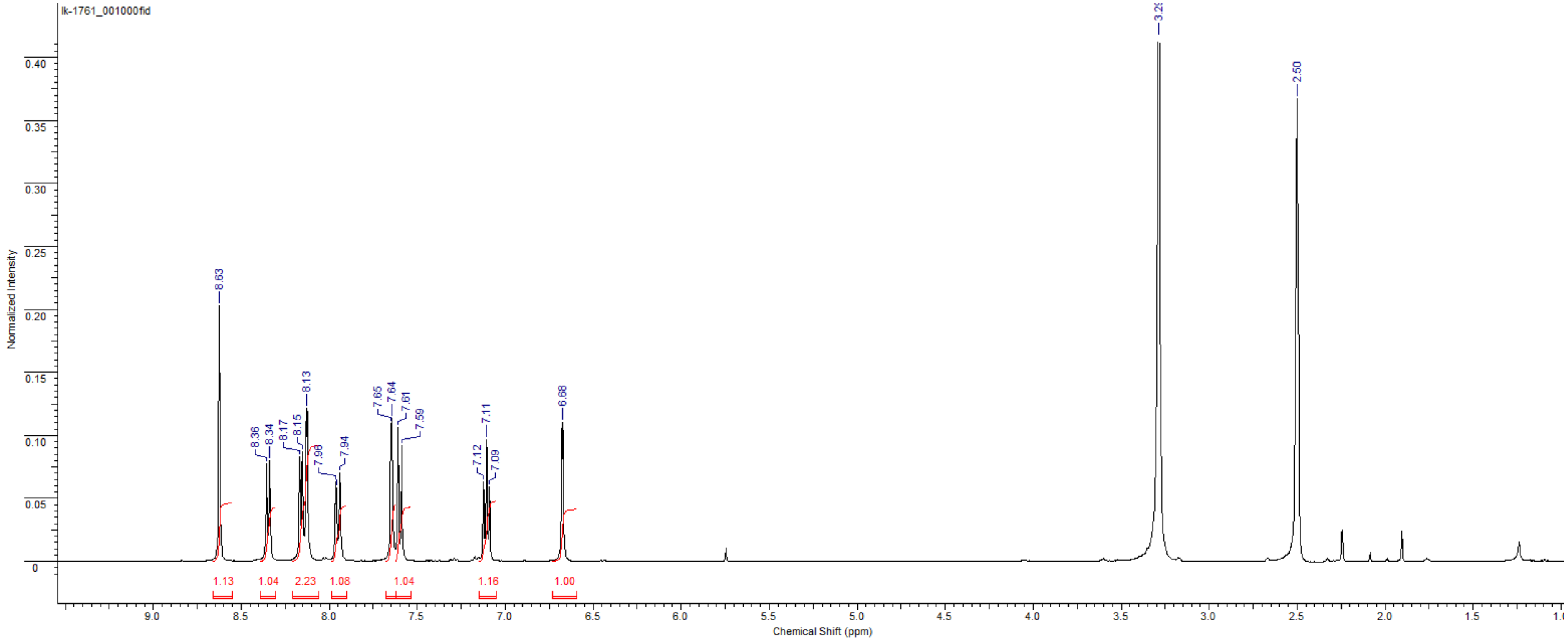

lk-1761_001000fid
Normalized Intensity
Chemical Shift (ppm)
8.63
8.36
8.34
8.17
8.15
8.13
7.98
7.94
7.65
7.64
7.61
7.59
7.12
7.11
7.09
6.68
2.50
1.13
1.04
2.23
1.08
1.04
1.16
1.00

4b

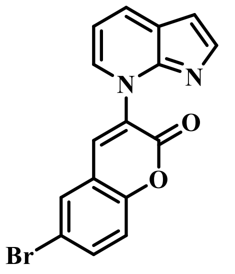

^{13}C

$CDCl_3 + CF_3COOH$

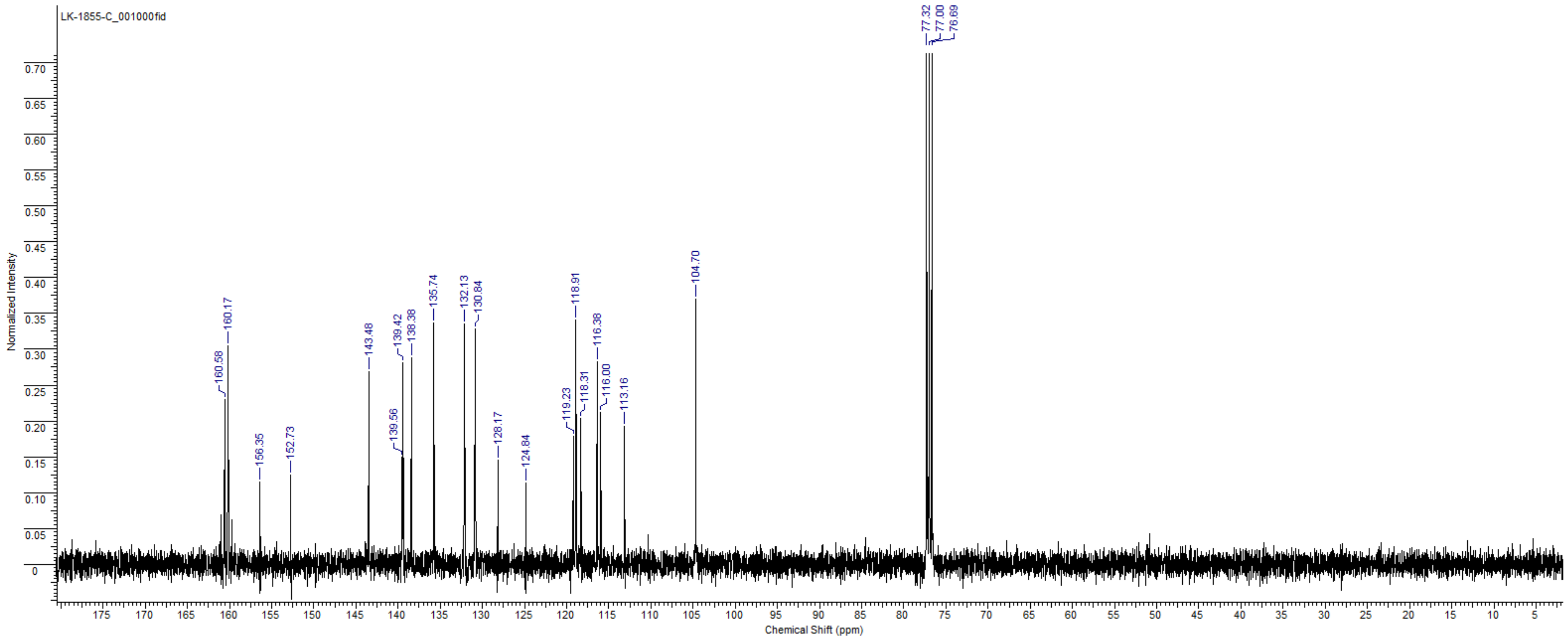

4c

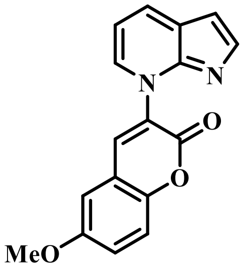
N
N
O
O
MeO

^{1}H

DMSO-d_6

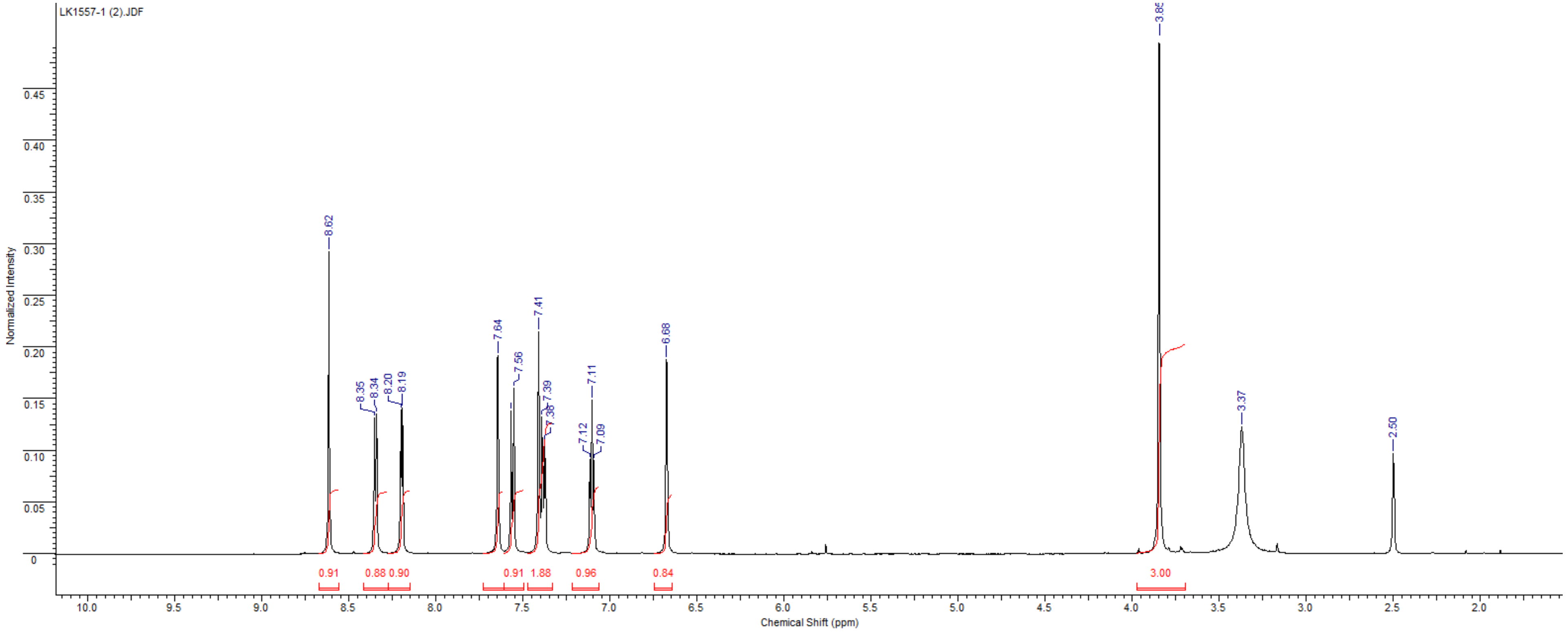
LK1557-1 (2).JDF
Normalized Intensity
Chemical Shift (ppm)
8.62
8.35
8.34
8.20
8.19
7.64
7.56
7.41
7.39
7.12
7.11
7.09
6.68
3.85
3.37
2.50
0.91
0.88
0.90
0.91
1.88
0.96
0.84
3.00

4c

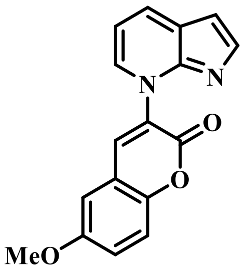

^{13}C

DMSO-d_6

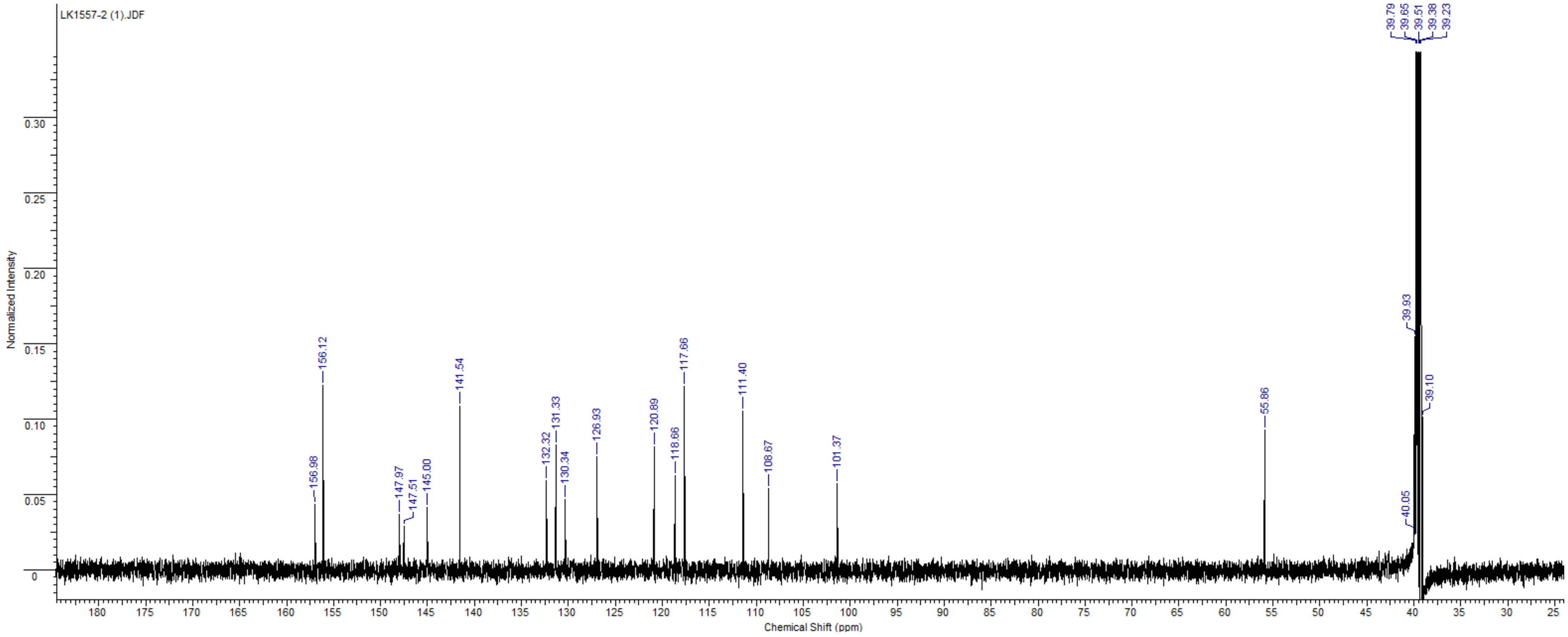

1b

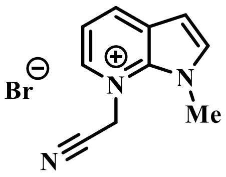
Br
N
N
Me
N

^{1}H

DMSO-d_6

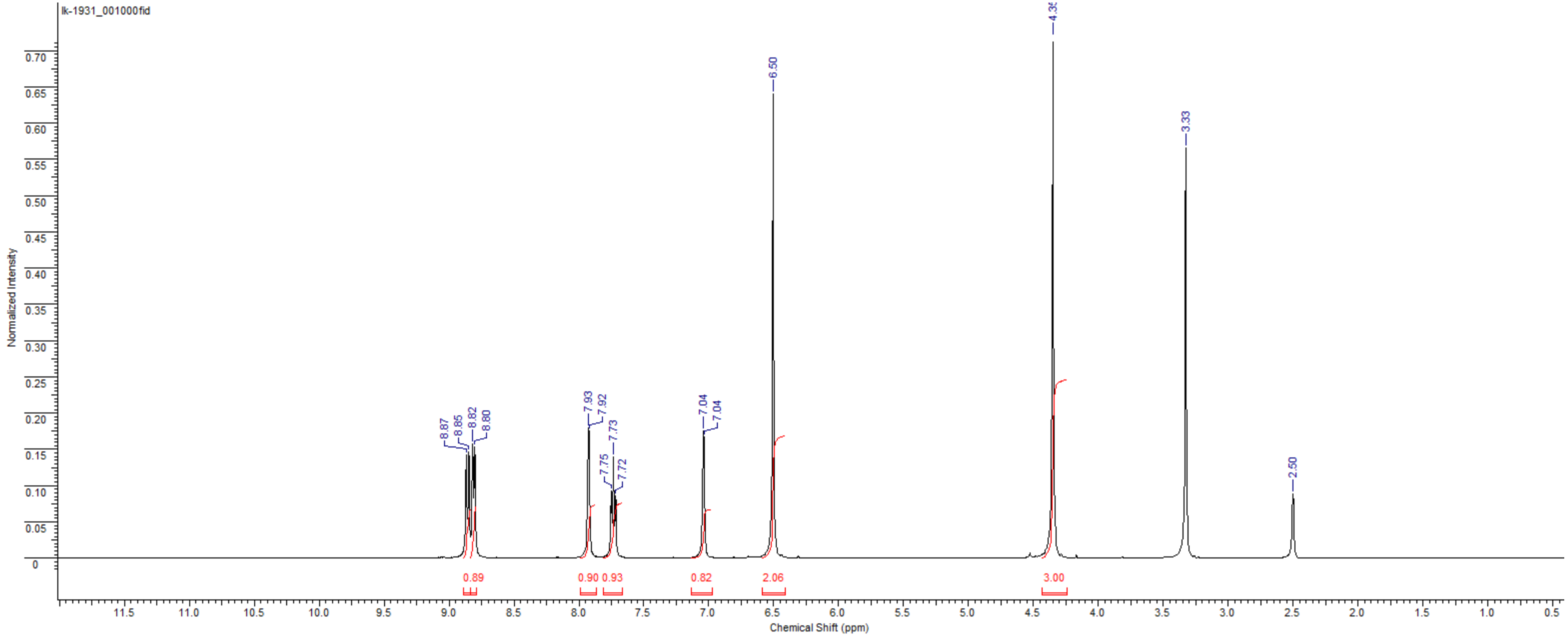
lk-1931_001000fid
Normalized Intensity
Chemical Shift (ppm)
8.87
8.85
8.82
8.80
7.93
7.92
7.75
7.73
7.72
7.04
7.04
6.50
4.35
3.33
2.50
0.89
0.90
0.93
0.82
2.06
3.00

1b

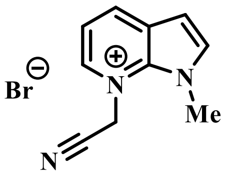

^{13}C

DMSO-d_6

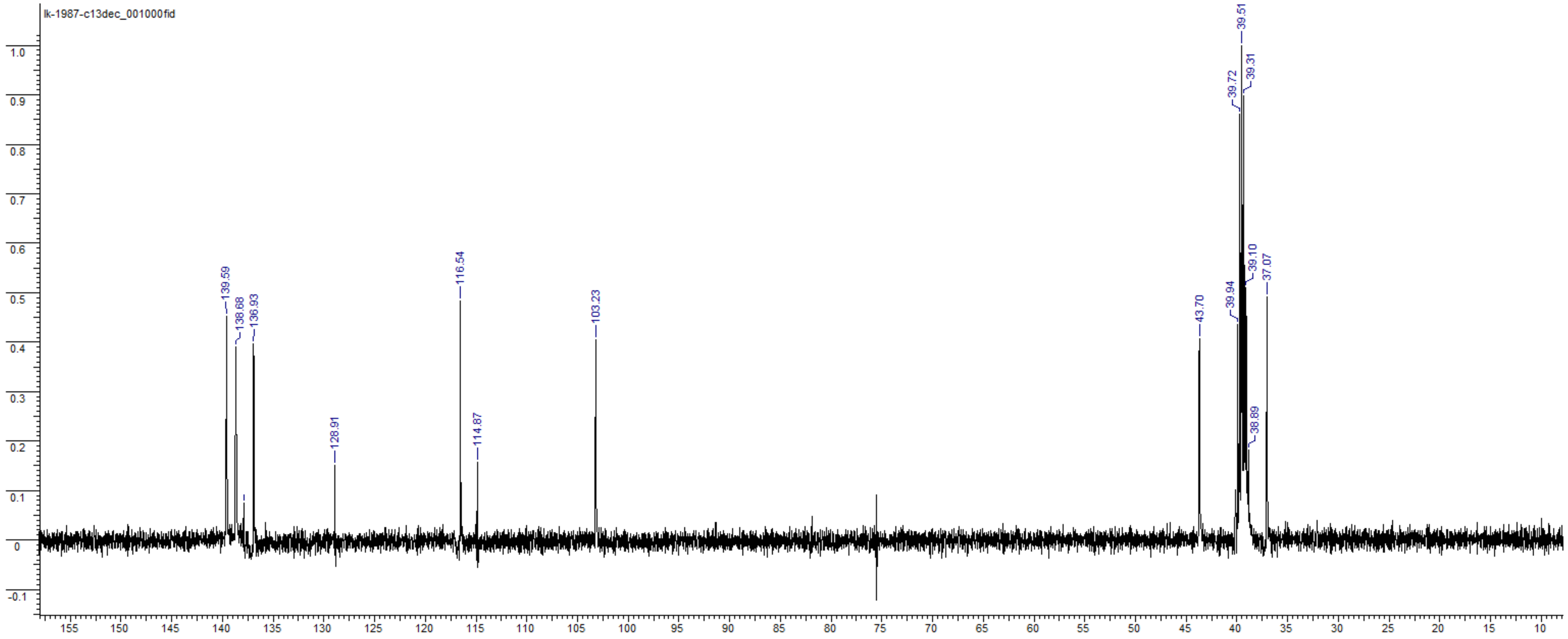

5a

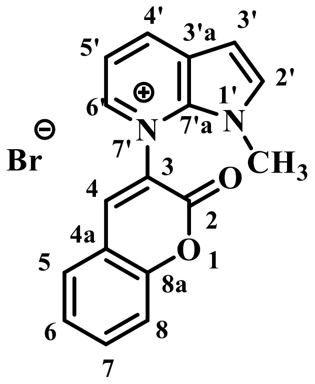

^{1}H

DMSO-d_6

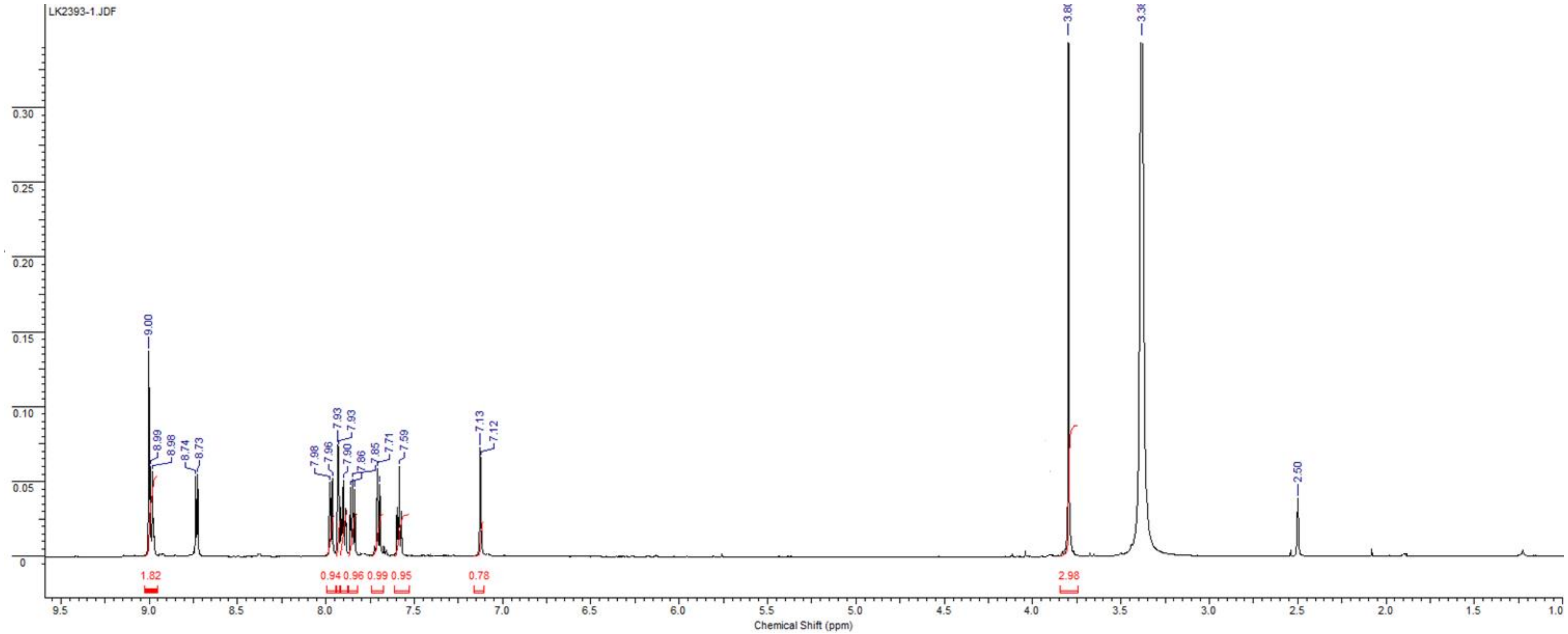
LK2393-1.JDF
9.00
8.99
8.98
8.74
8.73
7.98
7.96
7.93
7.93
7.90
7.86
7.85
7.71
7.59
7.13
7.12
3.80
3.38
2.50
1.82
0.94
0.96
0.99
0.95
0.78
2.98
Chemical Shift (ppm)

5a

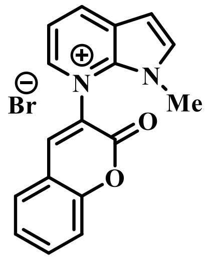

^{13}C

DMSO-d_6

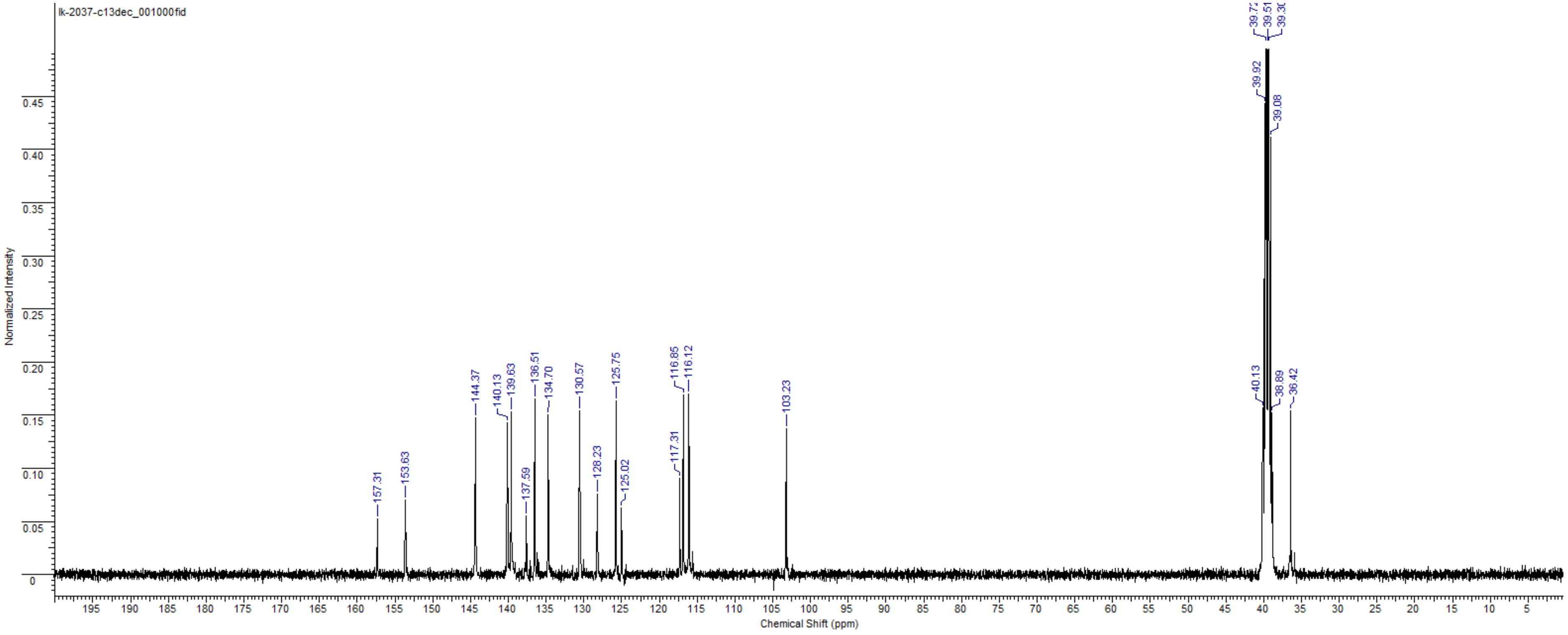

5b

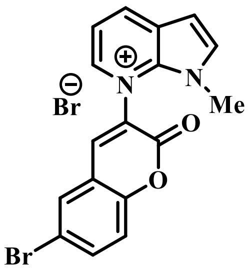
Br
N
N
Me
O
O
Br

^1H

DMSO-d_6

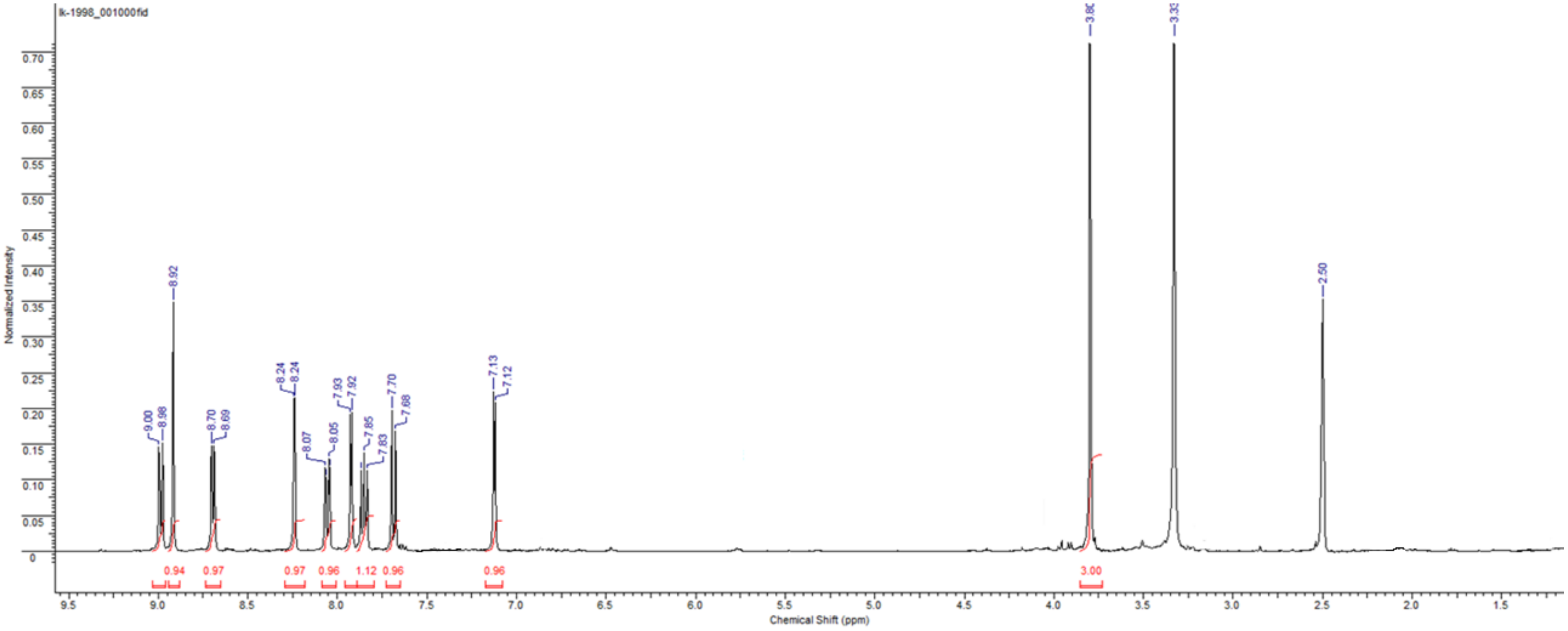
lk-1998_001000fid
Normalized Intensity
Chemical Shift (ppm)
9.00
8.98
8.92
8.70
8.69
8.24
8.24
8.07
8.05
7.93
7.92
7.85
7.83
7.70
7.68
7.13
7.12
3.80
3.33
2.50
0.94
0.97
0.97
0.96
1.12
0.96
0.96
3.00

5b

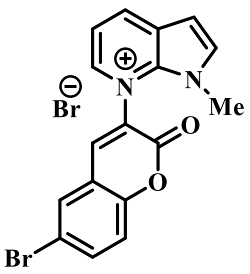

^{13}C

DMSO-d_6

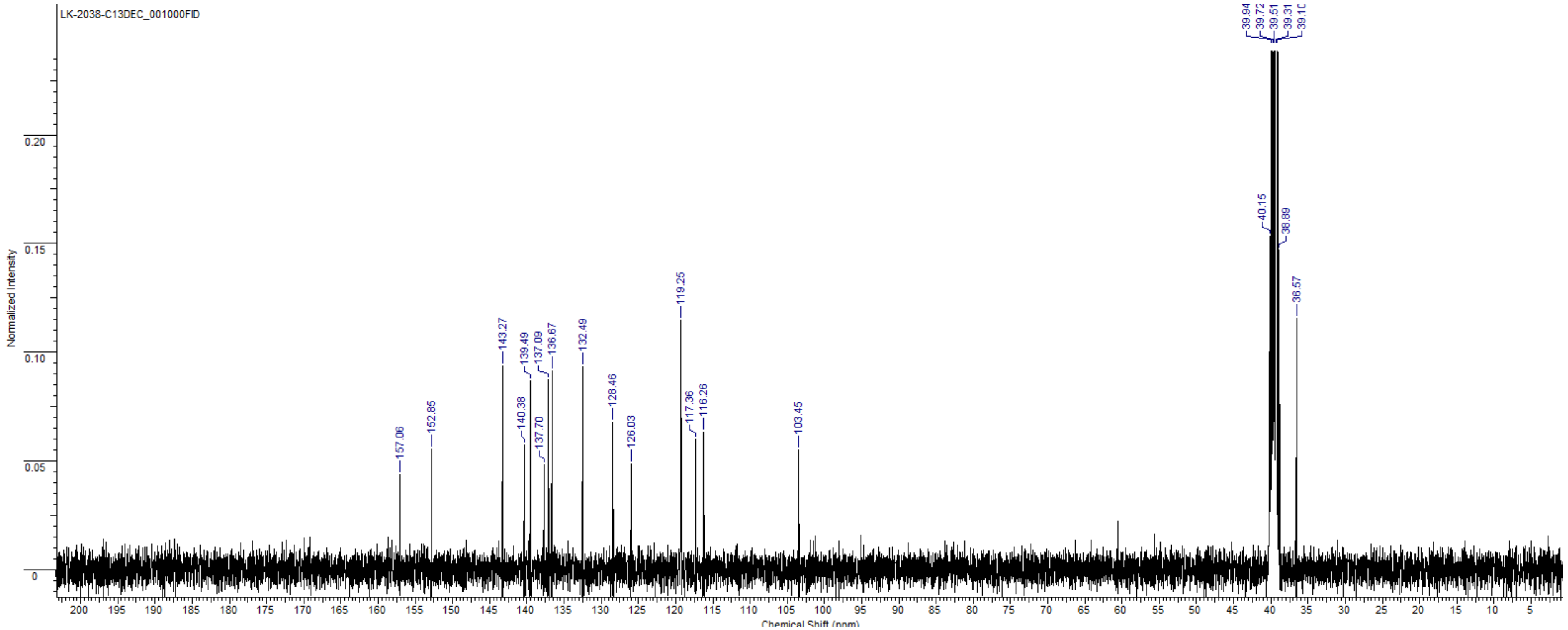

5c

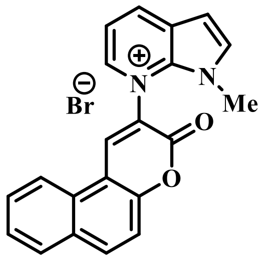

Br
N
N
Me
O
O

^{1}H

DMSO-d_6

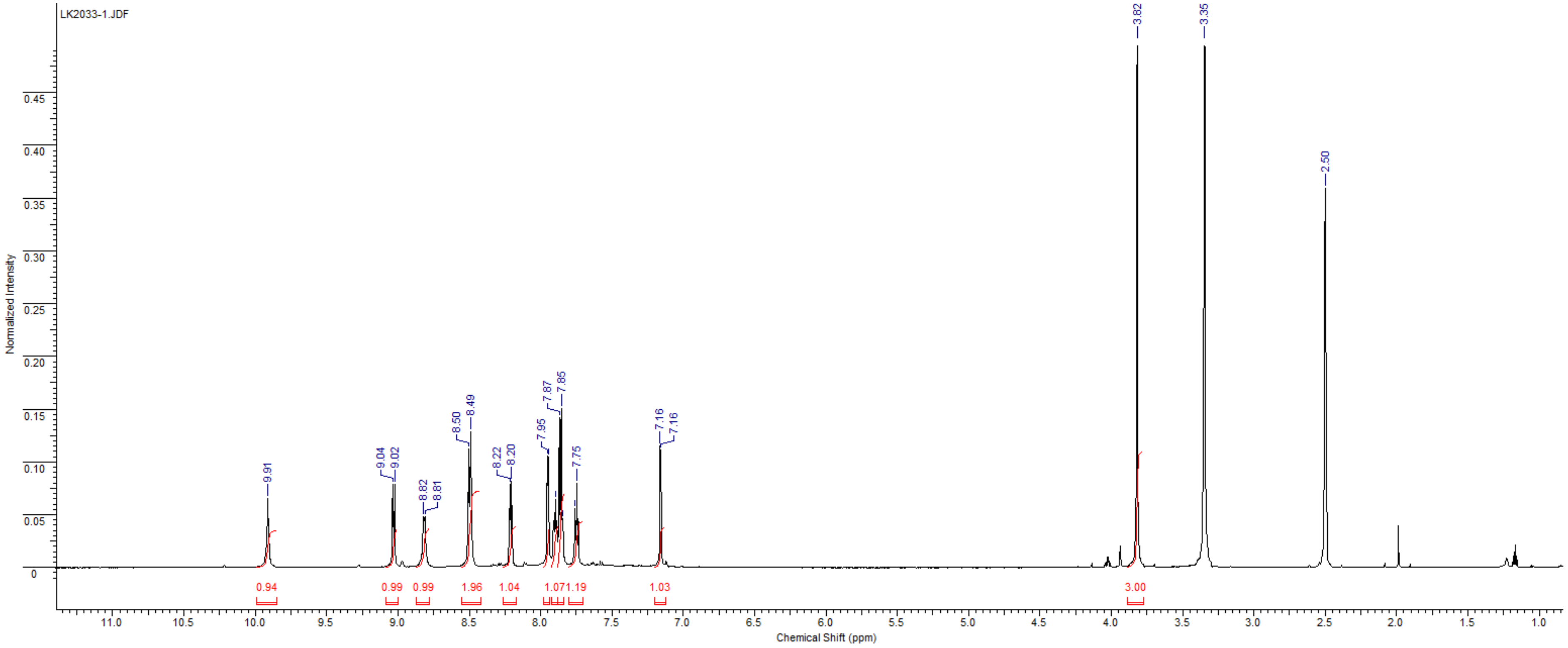

LK2033-1.JDF
Normalized Intensity
Chemical Shift (ppm)
9.91
9.04
9.02
8.82
8.81
8.50
8.49
8.22
8.20
7.95
7.87
7.85
7.75
7.16
7.16
3.82
3.35
2.50
0.94
0.99
0.99
1.96
1.04
1.07
1.19
1.03
3.00

5c

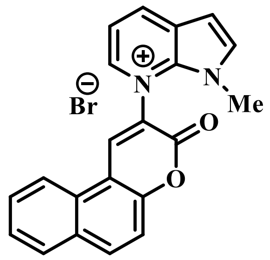

^{13}C

DMSO-d_6

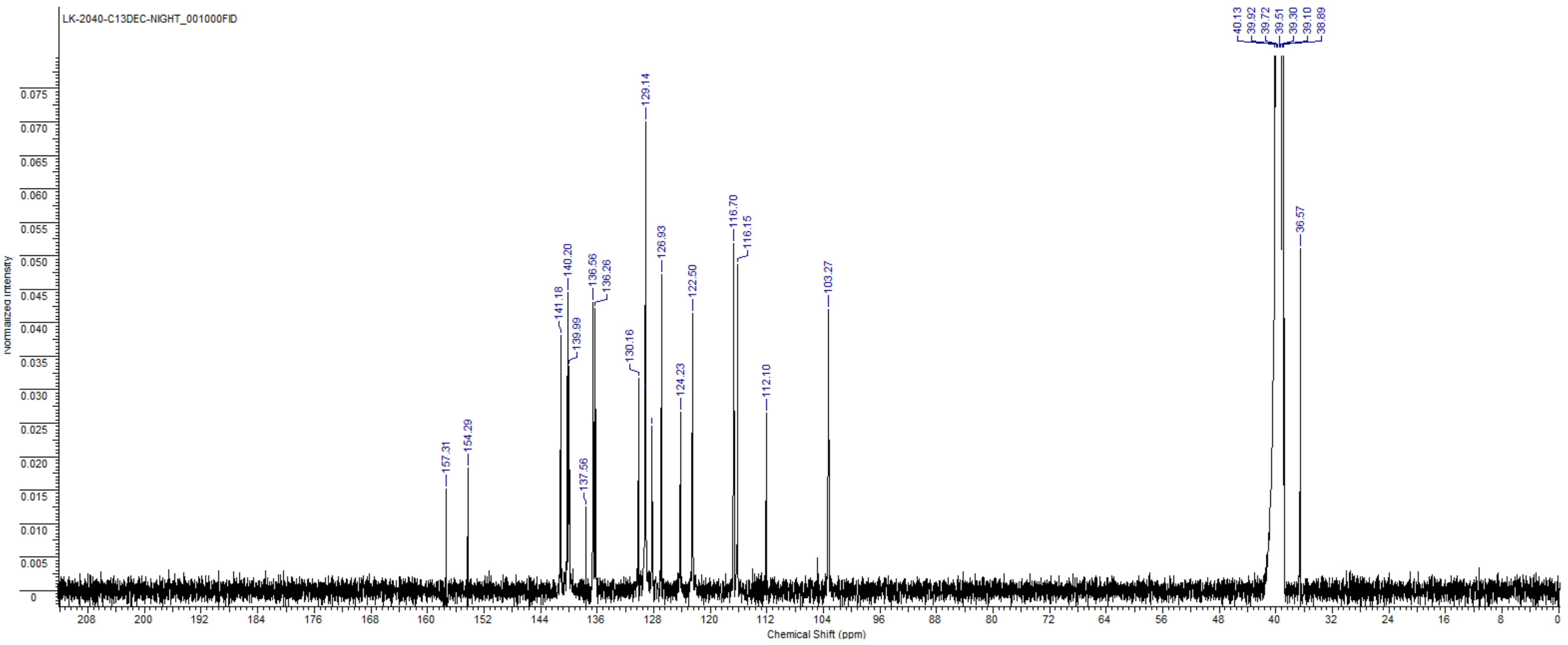

5d

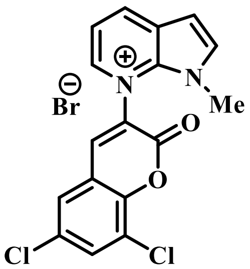

Br
N
N
Me
O
O
Cl
Cl

^{1}H

DMSO-d_6

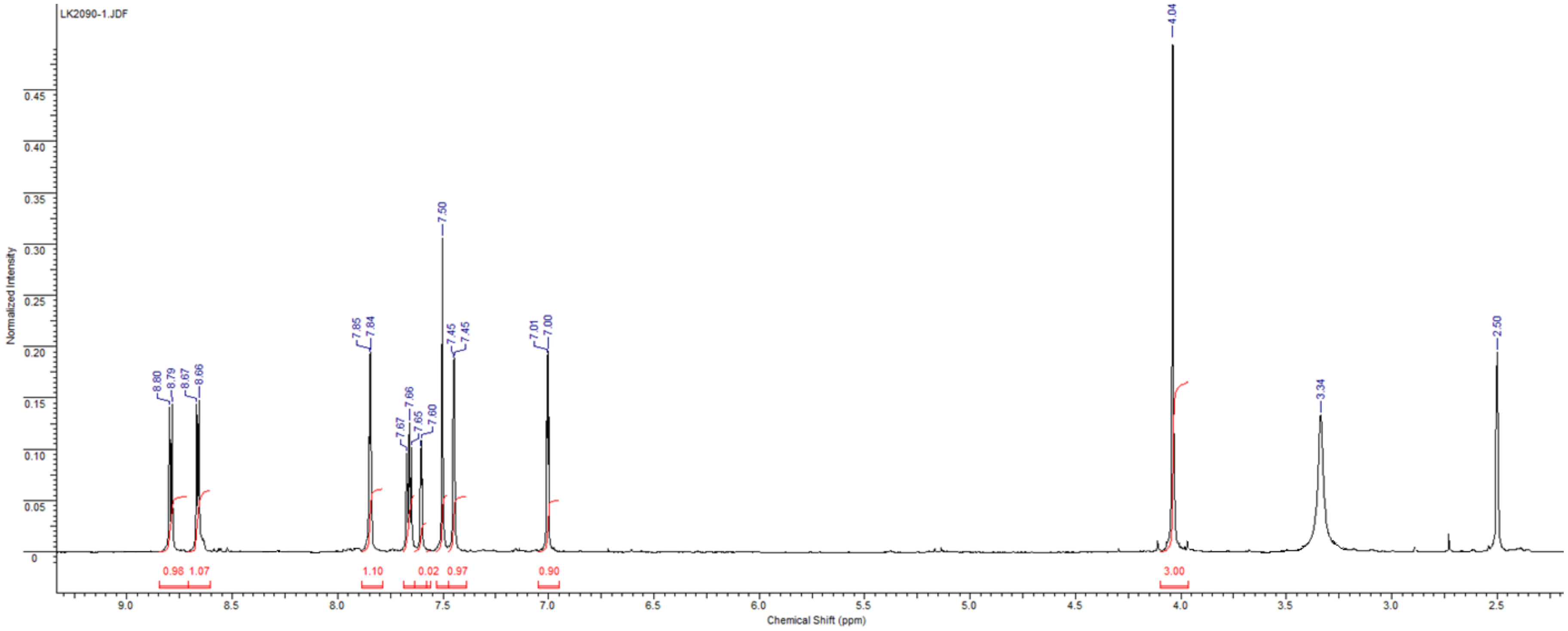

LK2090-1.JDF
Normalized Intensity
Chemical Shift (ppm)
8.80
8.79
8.67
8.66
7.85
7.84
7.67
7.66
7.65
7.60
7.50
7.45
7.45
7.01
7.00
4.04
3.34
2.50
0.98
1.07
1.10
0.02
0.97
0.90
3.00

5d

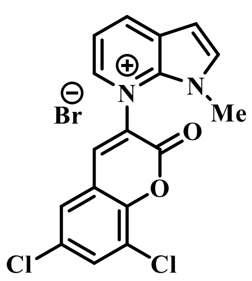

^{13}C

DMSO-d_6

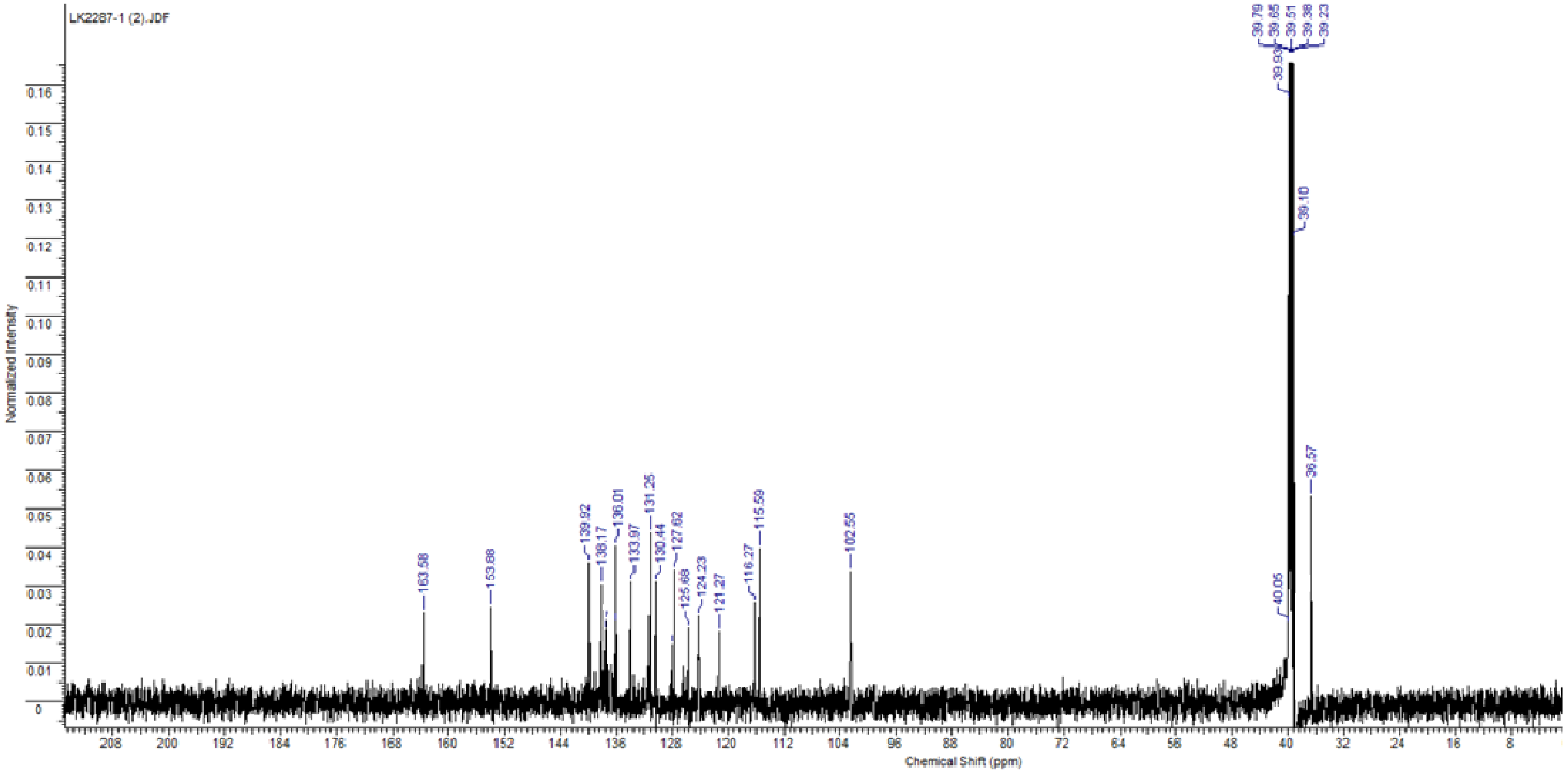

6a

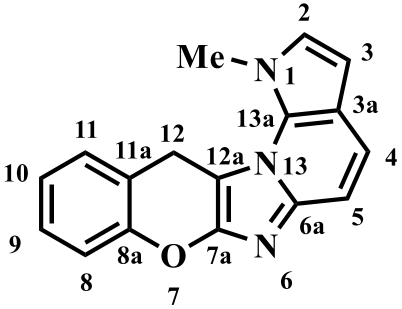
Me
N 1
2
3
3a
4
5
6a
N 13
13a
12a
12
11a
11
10
9
8
8a
O
7
7a
N
6

^{1}H

DMSO-d_6

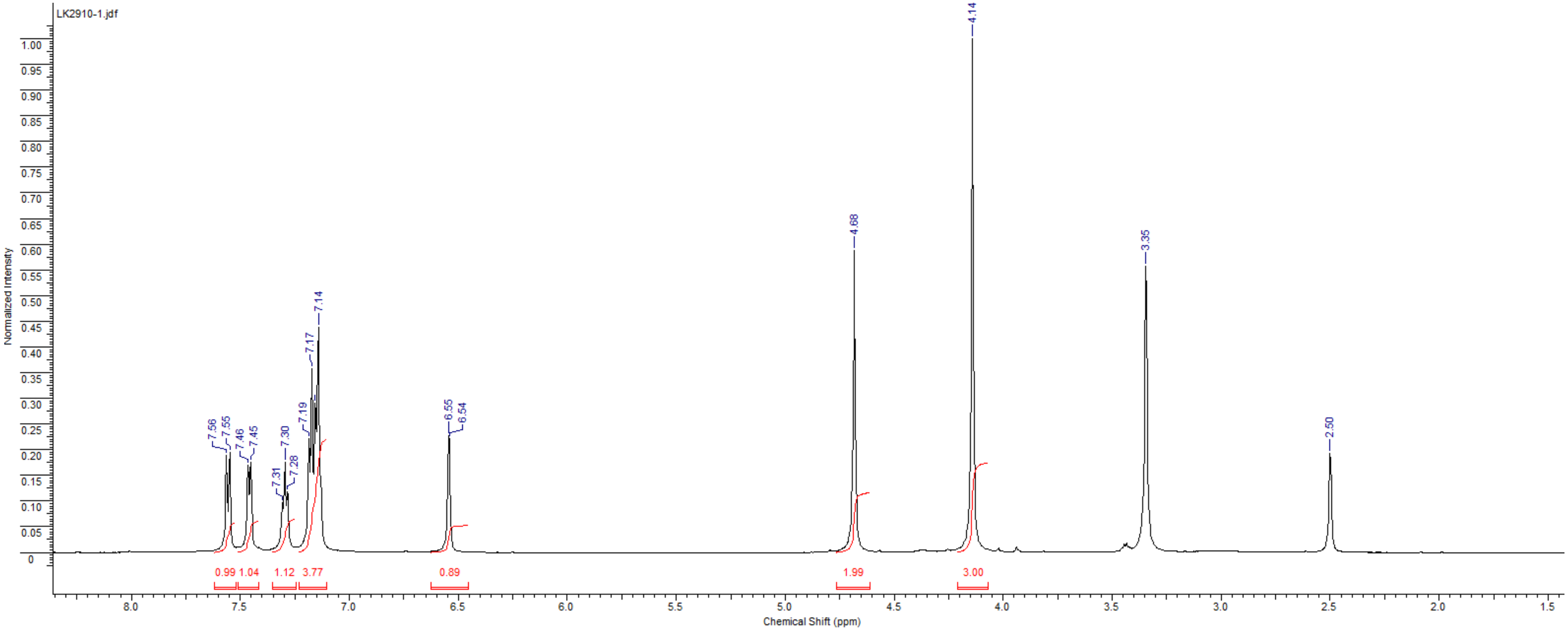
LK2910-1.jdf
Normalized Intensity
Chemical Shift (ppm)
7.56
7.55
7.46
7.45
7.31
7.30
7.28
7.19
7.17
7.14
6.55
6.54
4.68
4.14
3.35
2.50
0.99
1.04
1.12
3.77
0.89
1.99
3.00

6a

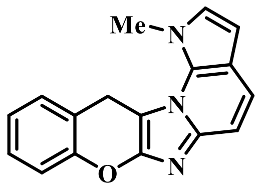

^{13}C

DMSO-d_6

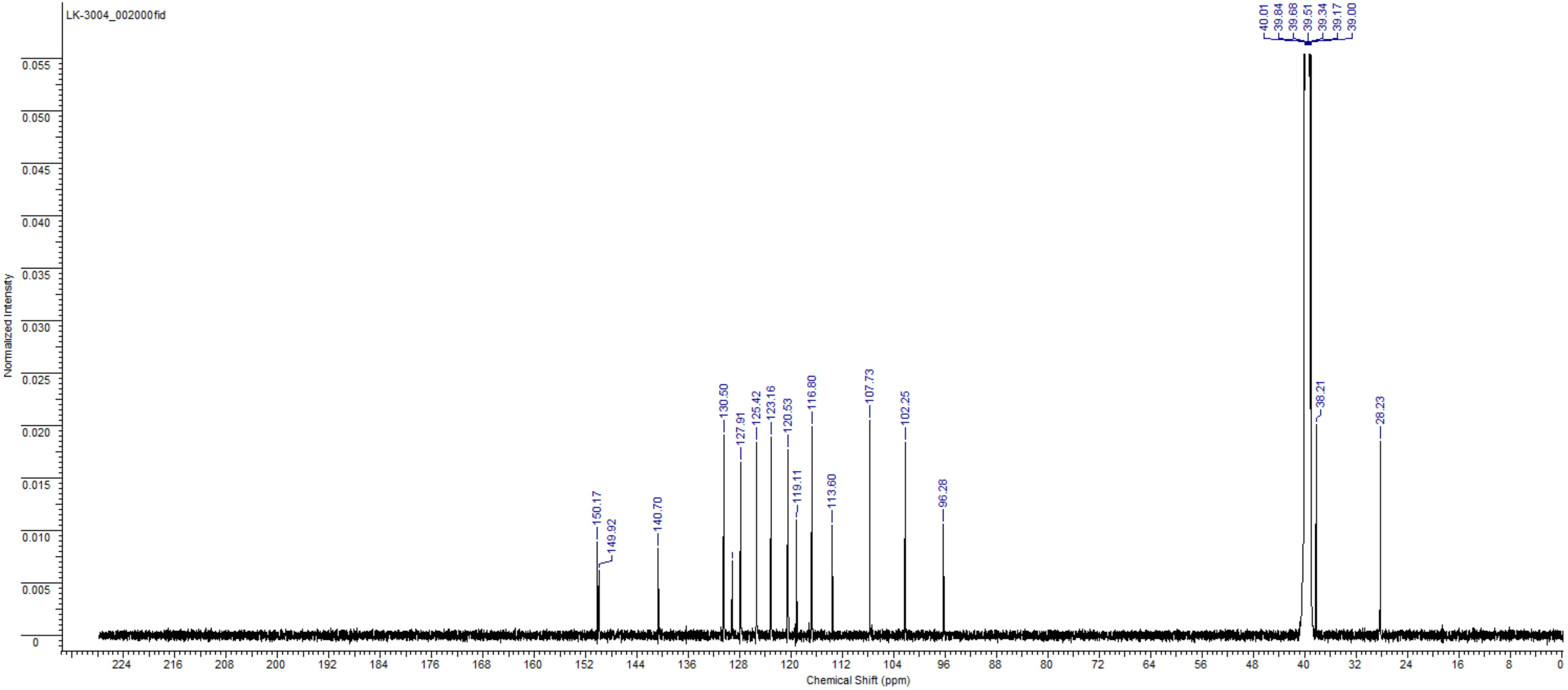

6b

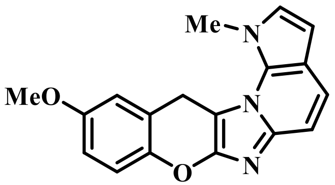
Me
N
MeO
N
O
N

^{1}H

DMSO-d_6

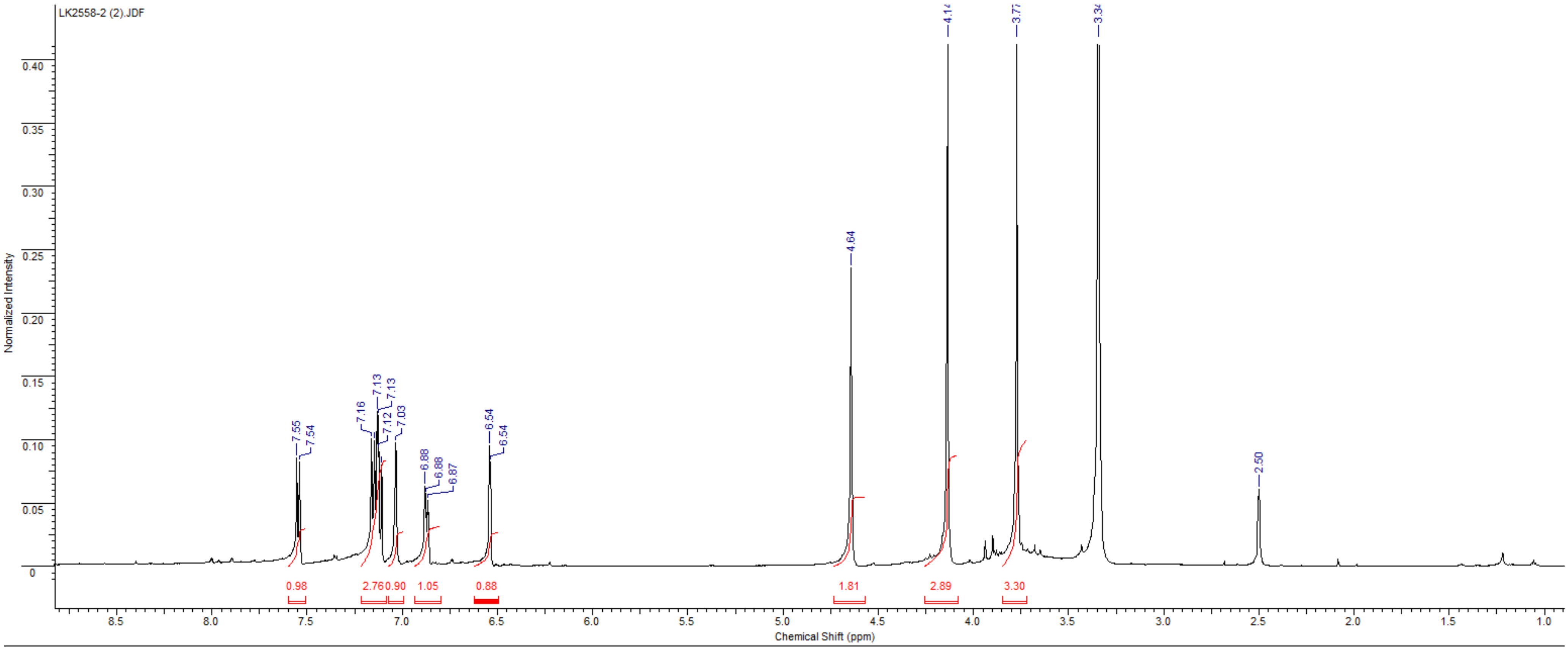
LK2558-2 (2).JDF
Normalized Intensity
Chemical Shift (ppm)
7.55
7.54
7.16
7.13
7.13
7.12
7.03
6.88
6.88
6.87
6.54
6.54
4.64
4.14
3.77
3.34
2.50
0.98
2.76
0.90
1.05
0.88
1.81
2.89
3.30

6b

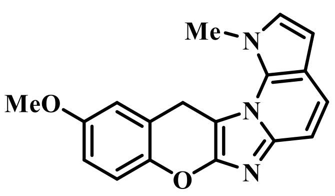

^{13}C

DMSO-d_6

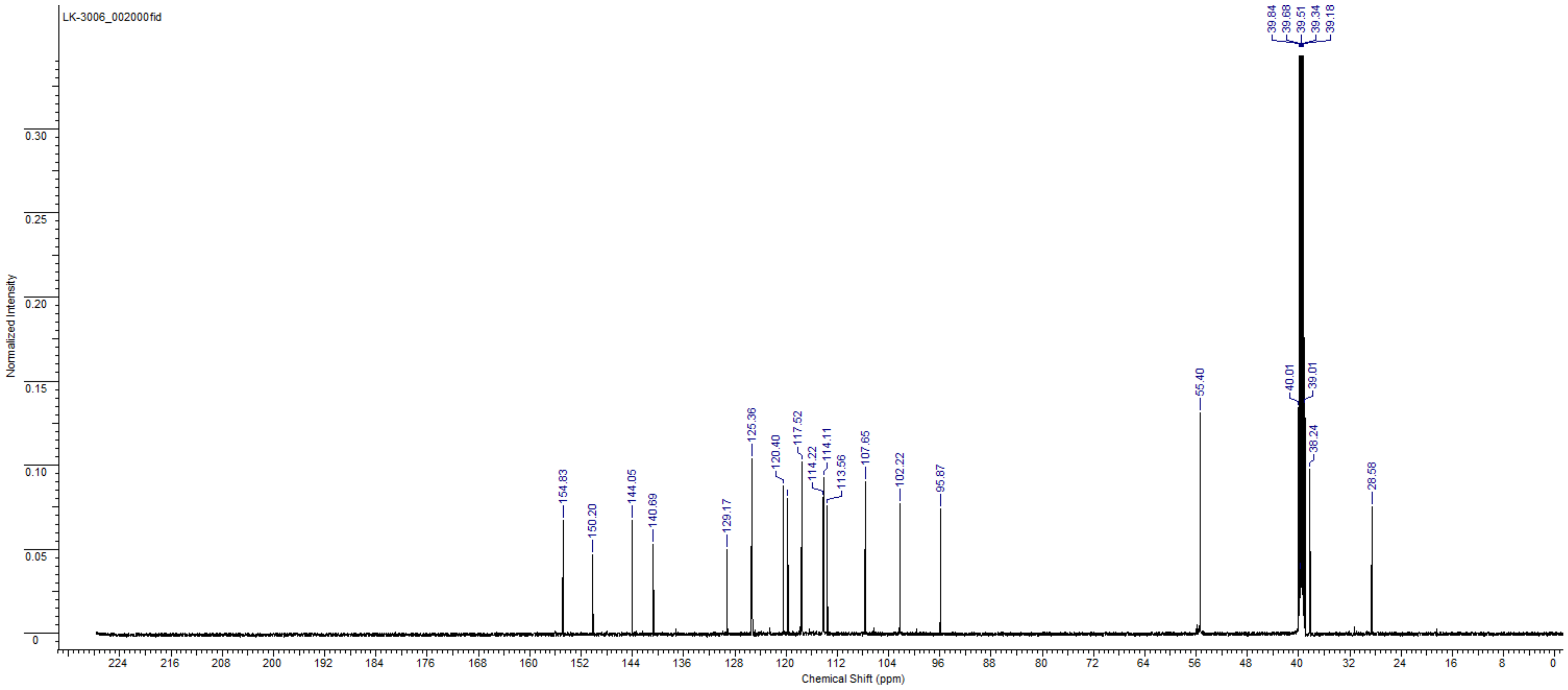

6c

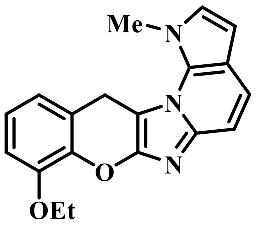

^{1}H

DMSO-d_6

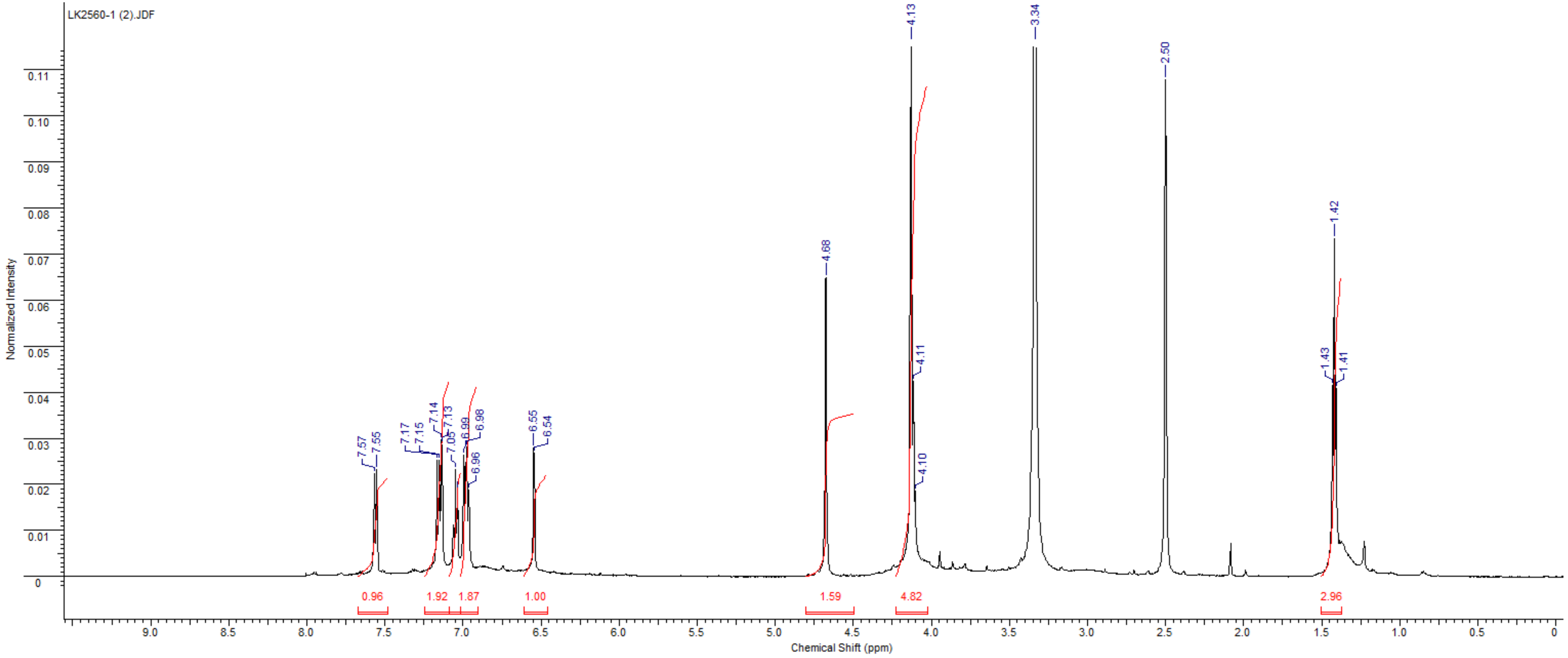
LK2560-1 (2).JDF
Normalized Intensity
Chemical Shift (ppm)
7.57
7.55
7.17
7.15
7.14
7.13
7.05
6.99
6.98
6.96
6.55
6.54
4.68
4.13
4.11
4.10
3.34
2.50
1.43
1.42
1.41
0.96
1.92
1.87
1.00
1.59
4.82
2.96

6c

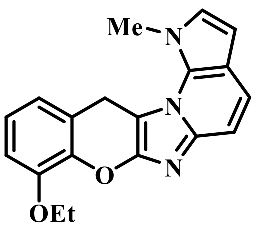

^{13}C

DMSO-d_6

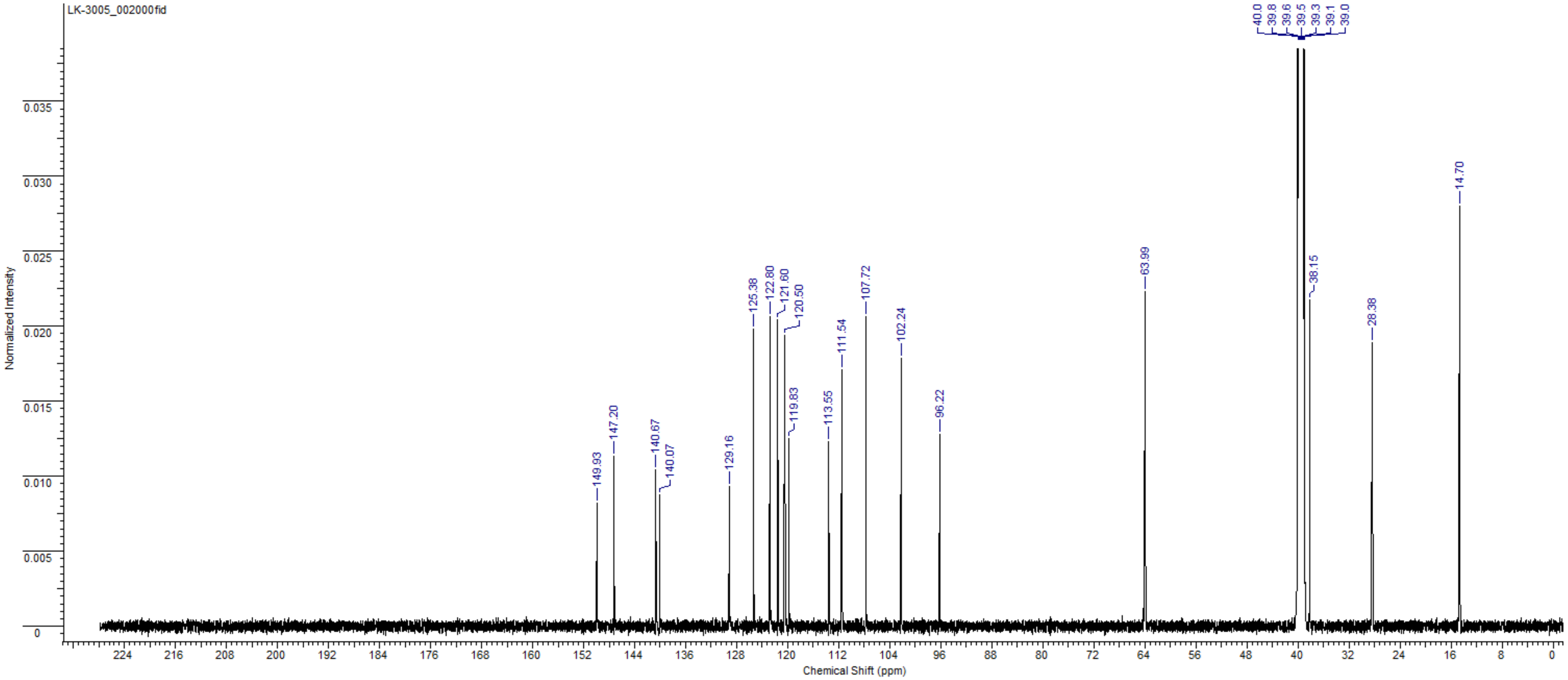

2

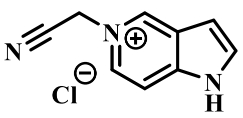
N
N
Cl
N
H

^{1}H

DMSO-d_6

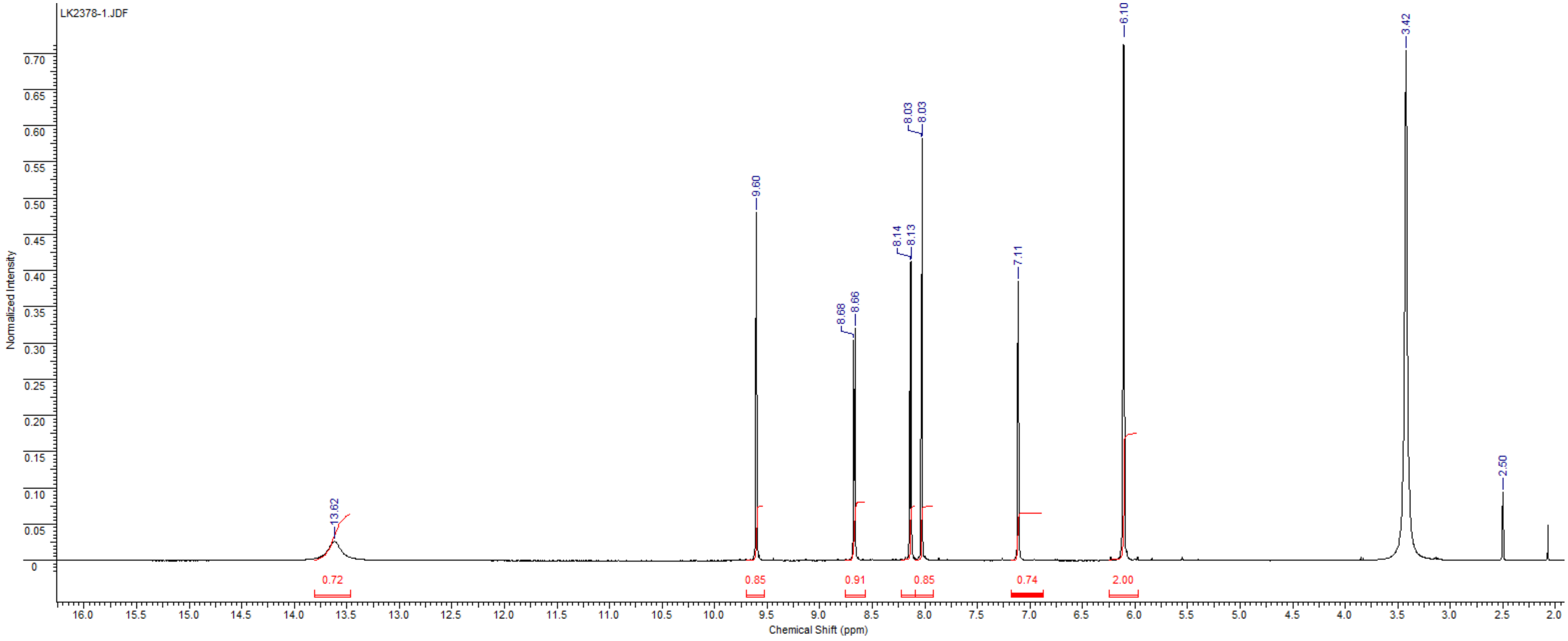
LK2378-1.JDF
Normalized Intensity
Chemical Shift (ppm)
13.62
9.60
8.68
8.66
8.14
8.13
8.03
8.03
7.11
6.10
3.42
2.50
0.72
0.85
0.91
0.85
0.74
2.00

2

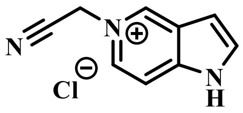

^{13}C

DMSO-d_6

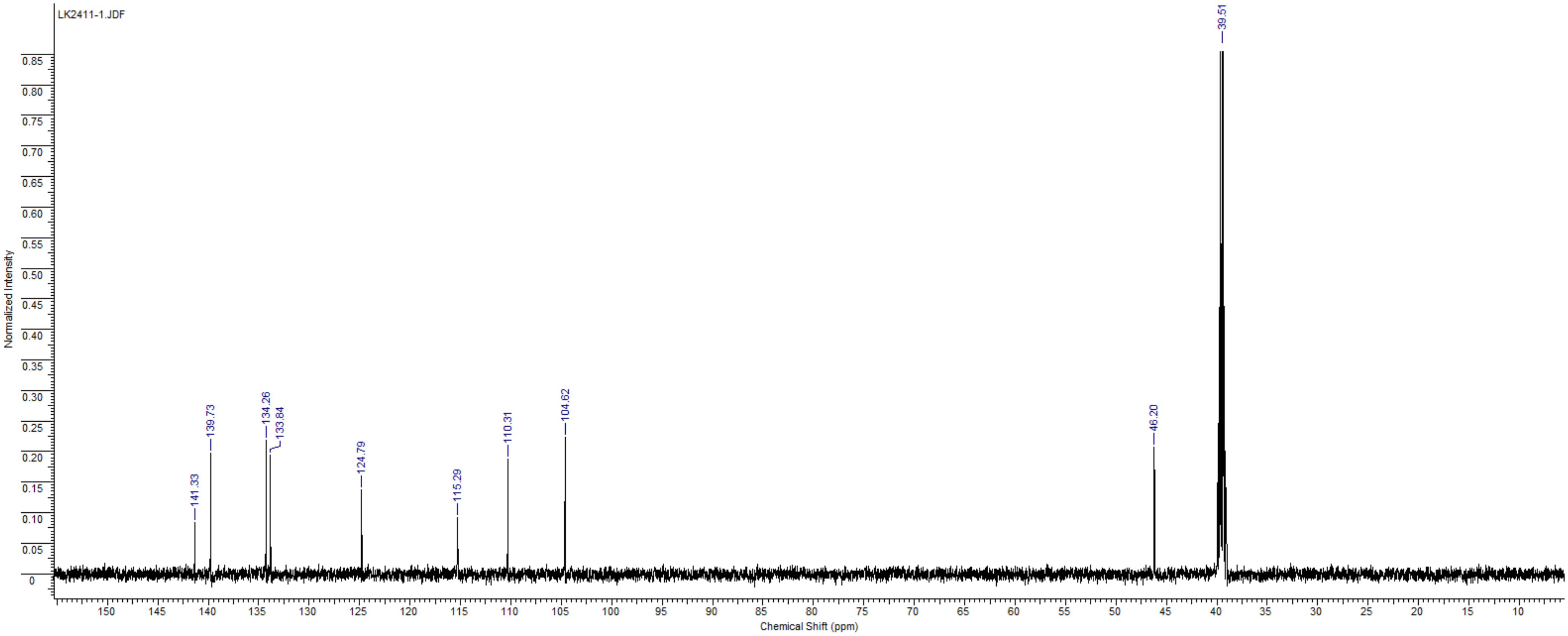
LK2411-1.JDF
141.33
139.73
134.26
133.84
124.79
115.29
110.31
104.62
46.20
39.51
Normalized Intensity
Chemical Shift (ppm)

7a

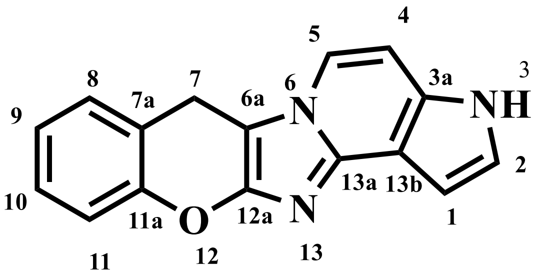

[1]H

DMSO-d_6

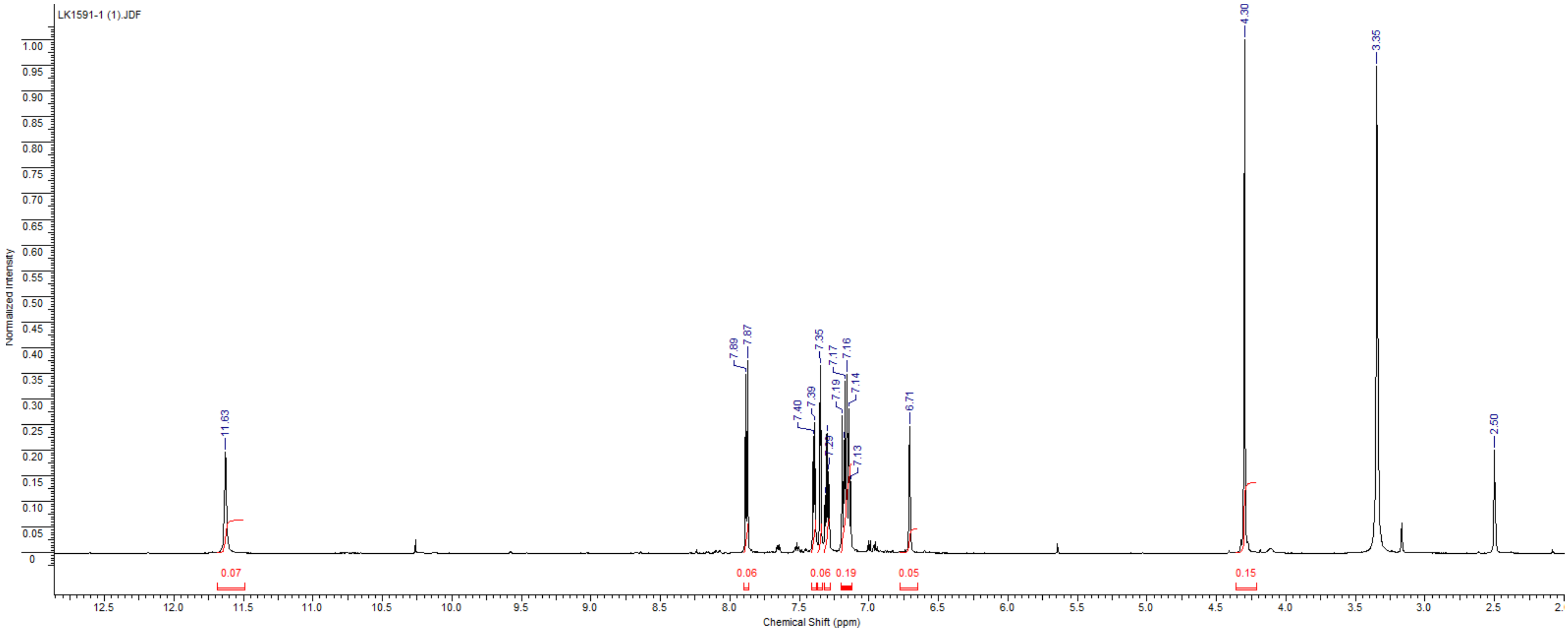
LK1591-1 (1).JDF
Normalized Intensity
Chemical Shift (ppm)
11.63
7.89
7.87
7.40
7.39
7.35
7.29
7.19
7.17
7.16
7.14
7.13
6.71
4.30
3.35
2.50
0.07
0.06
0.06
0.19
0.05
0.15

7a

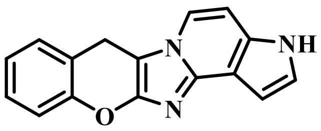

^{13}C

DMSO-d_6, 45°C

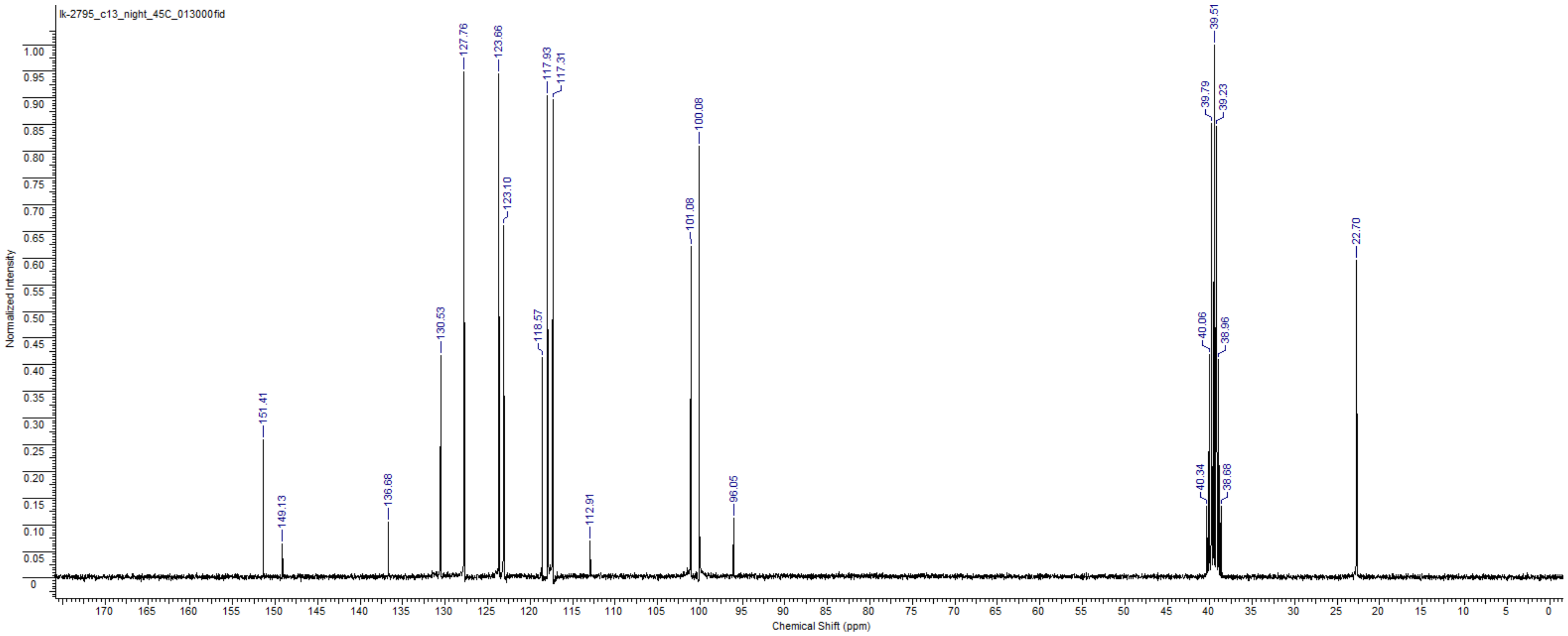

7b

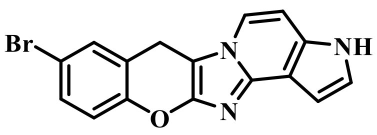
Br
NH
N
N
O

^{1}H

DMSO-d_6

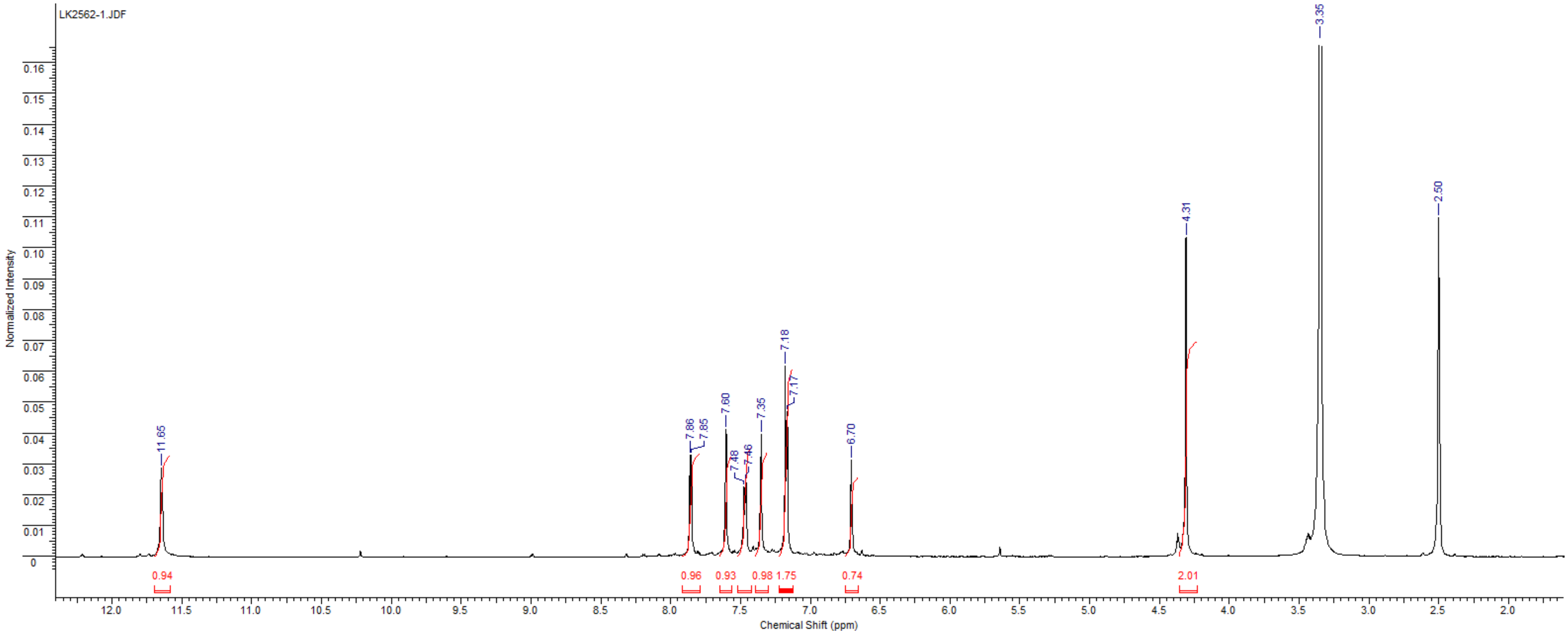
LK2562-1.JDF
Normalized Intensity
Chemical Shift (ppm)
11.65
7.86
7.85
7.60
7.48
7.46
7.35
7.18
7.17
6.70
4.31
3.35
2.50
0.94
0.96
0.93
0.98
1.75
0.74
2.01

7b

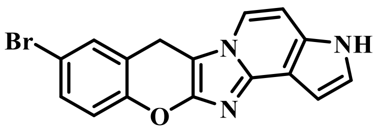

^{13}C

DMSO-d_6

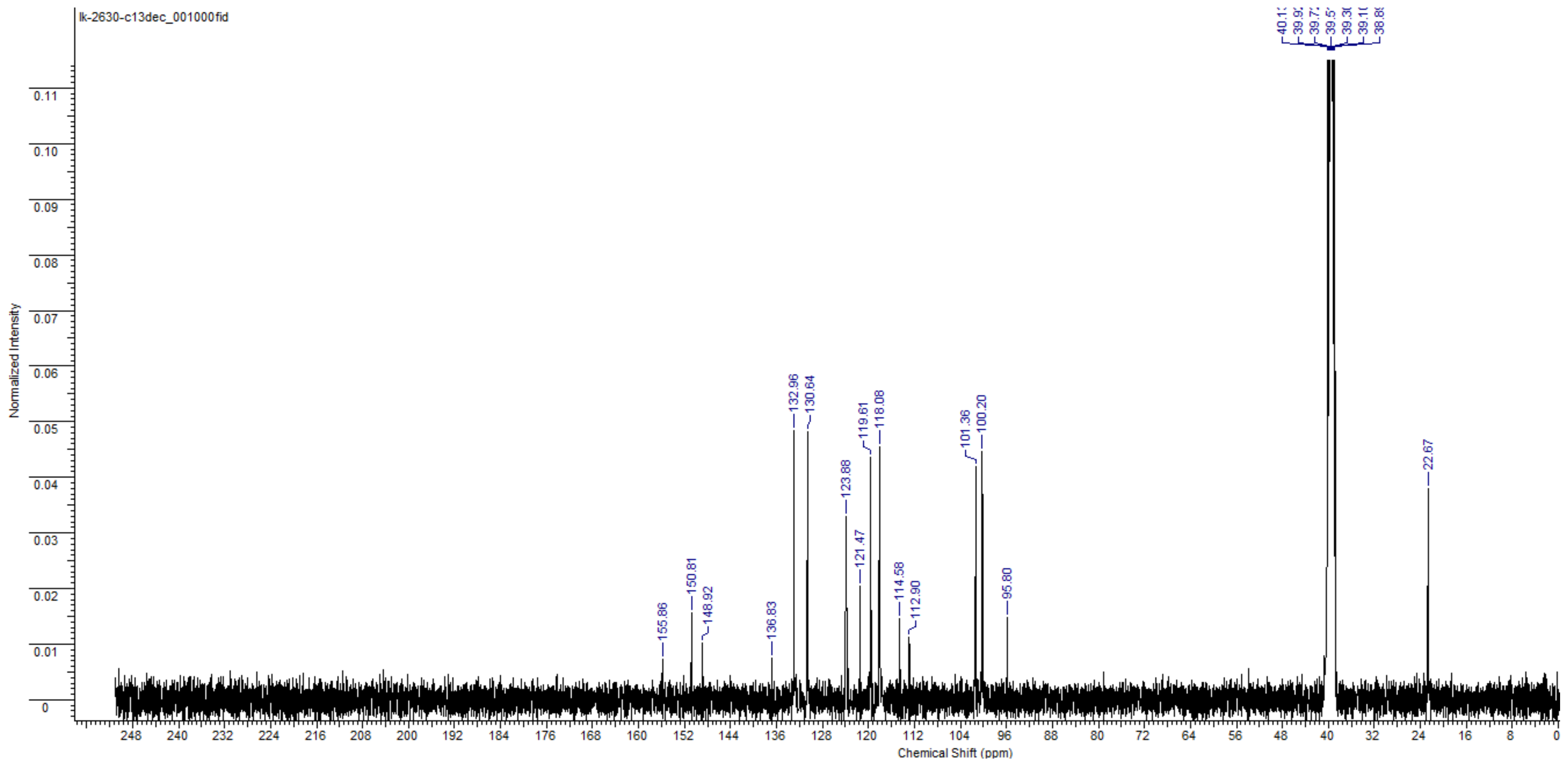

7c

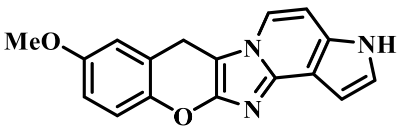
MeO
N
NH
O
N

^{1}H

DMSO-d_6

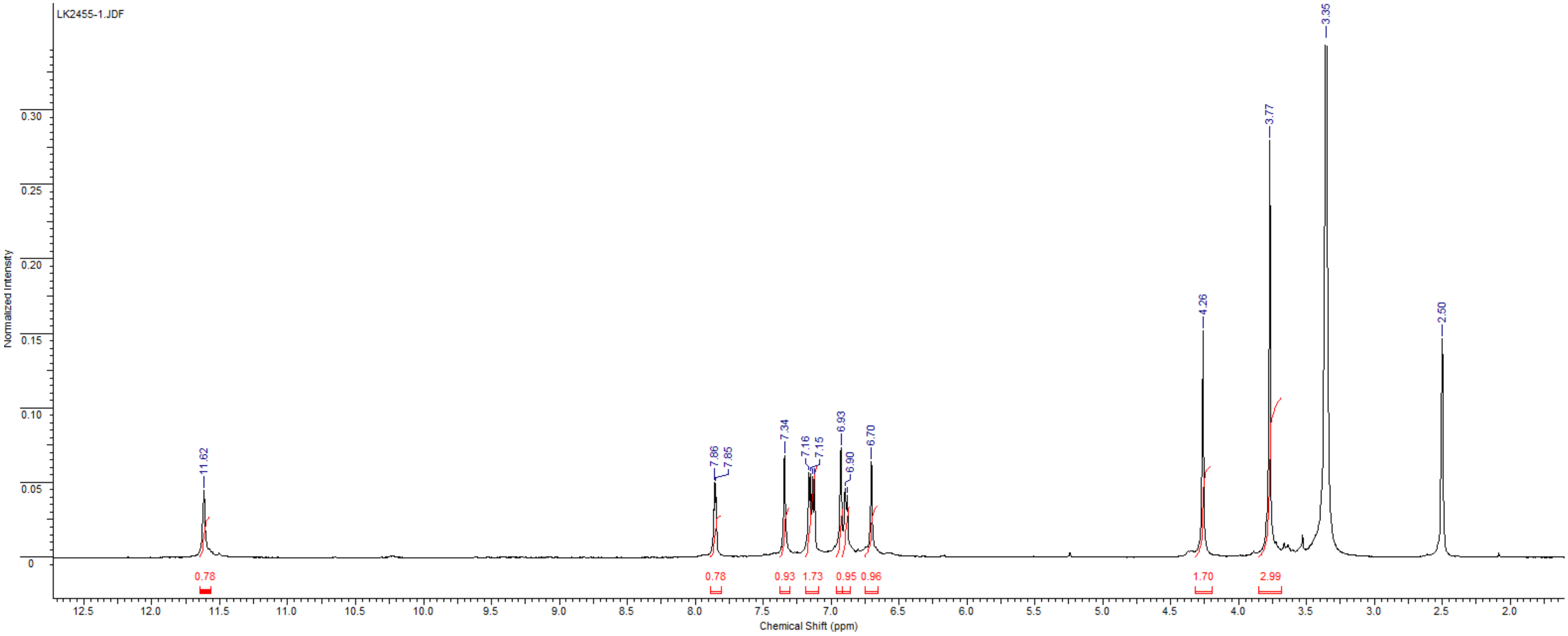
LK2455-1.JDF
Normalized Intensity
Chemical Shift (ppm)
11.62
7.86
7.85
7.34
7.16
7.15
6.93
6.90
6.70
4.26
3.77
3.35
2.50
0.78
0.78
0.93
1.73
0.95
0.96
1.70
2.99

7c

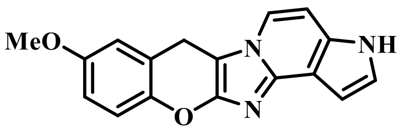

^{13}C

DMSO-d_6, 45^oC

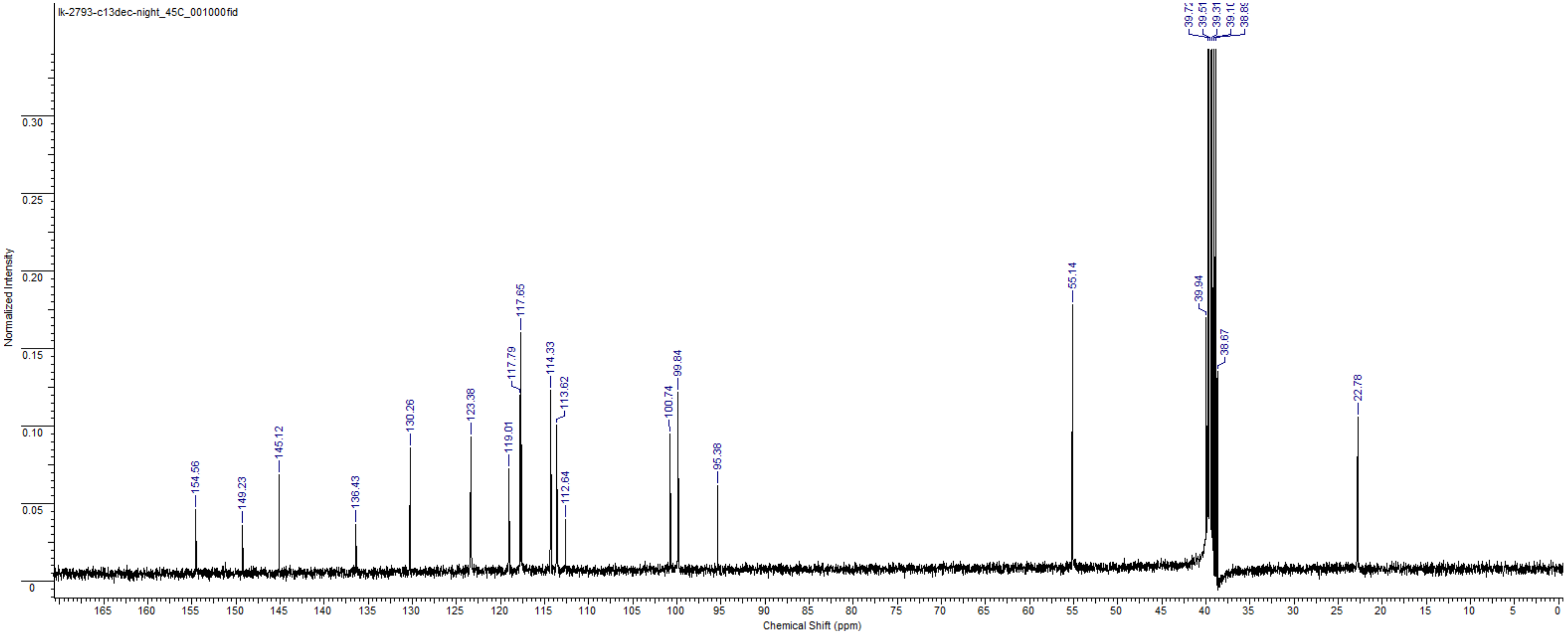

7d

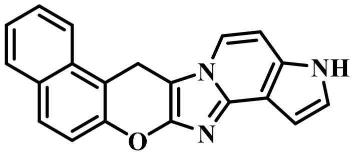

^{1}H

DMSO-d_6

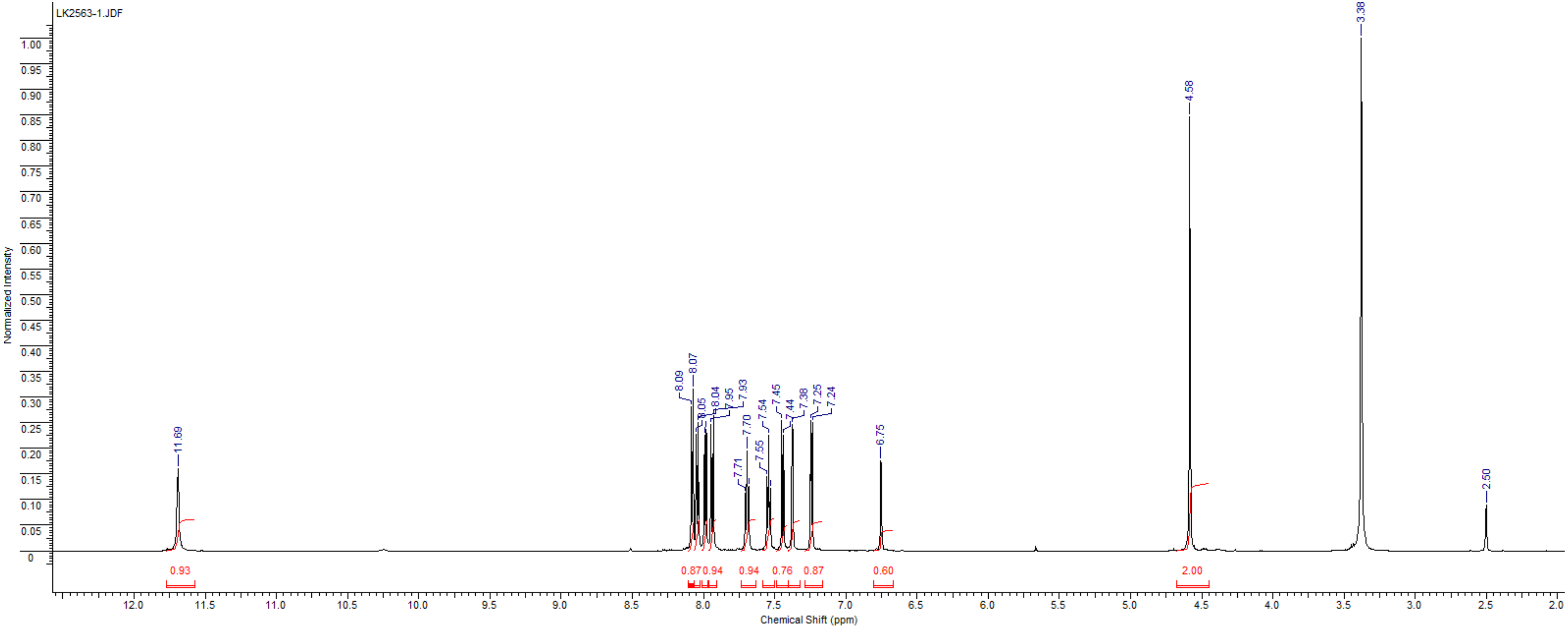
LK2563-1.JDF
Normalized Intensity
Chemical Shift (ppm)
11.69
8.09
8.07
8.05
8.04
7.95
7.93
7.71
7.70
7.55
7.54
7.45
7.44
7.38
7.25
7.24
6.75
4.58
3.38
2.50
0.93
0.87
0.94
0.94
0.76
0.87
0.60
2.00

7d

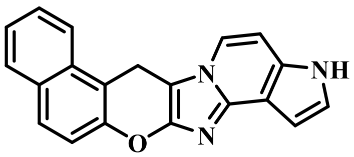

^{13}C

DMSO-d_6

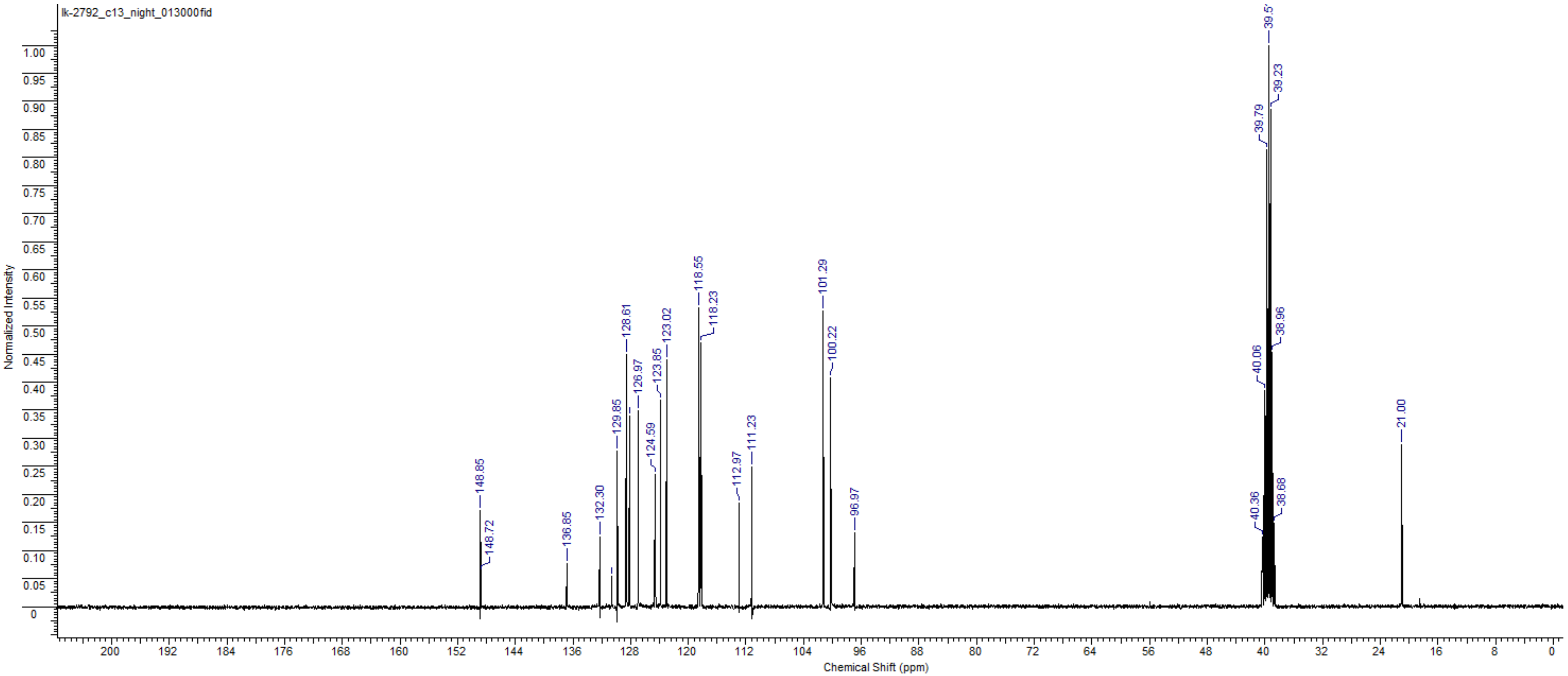

7e

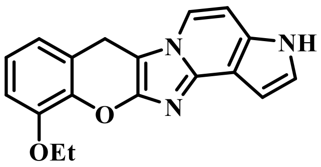
NH
N
N
O
OEt

^{1}H

DMSO-d_6

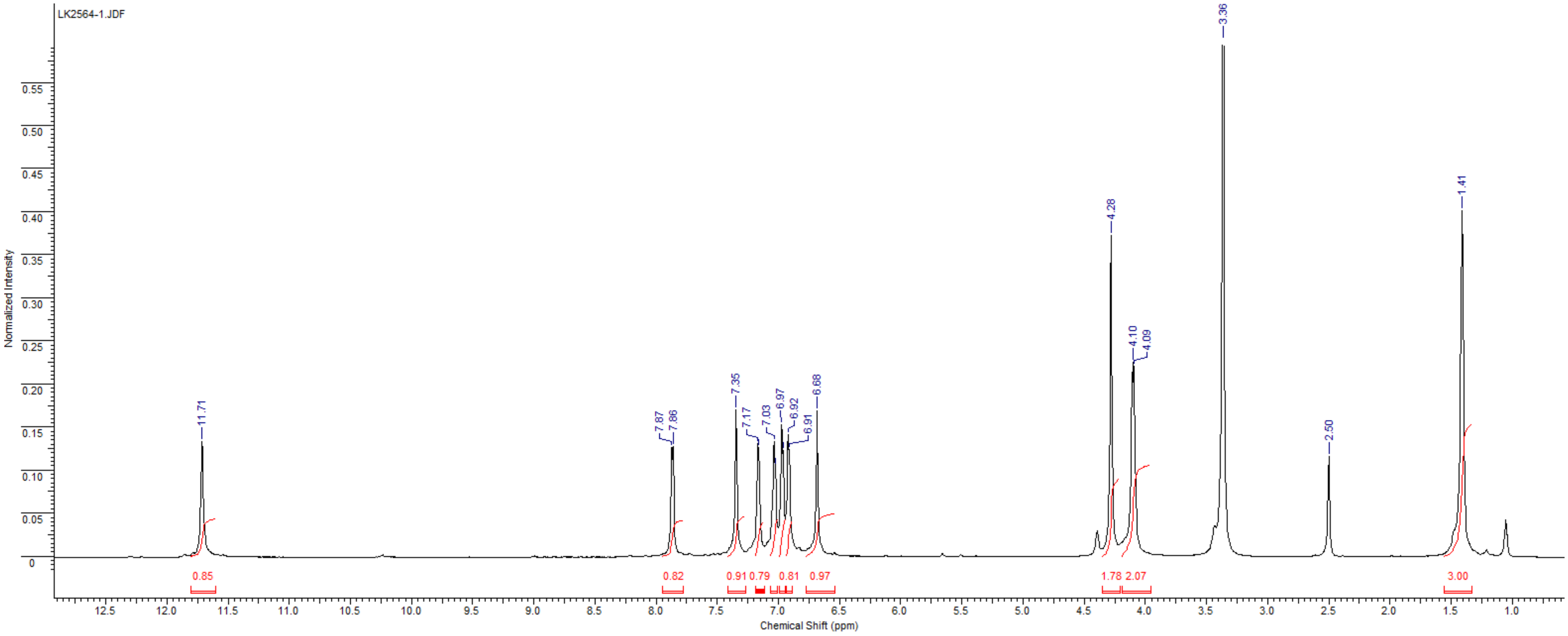
LK2564-1.JDF
Normalized Intensity
Chemical Shift (ppm)
11.71
7.87
7.86
7.35
7.17
7.03
6.97
6.92
6.91
6.68
4.28
4.10
4.09
3.36
2.50
1.41
0.85
0.82
0.91
0.79
0.81
0.97
1.78
2.07
3.00

3

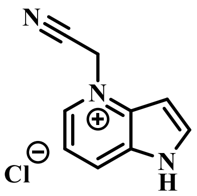

N
N
Cl
N
H

^{1}H

DMSO-d_6

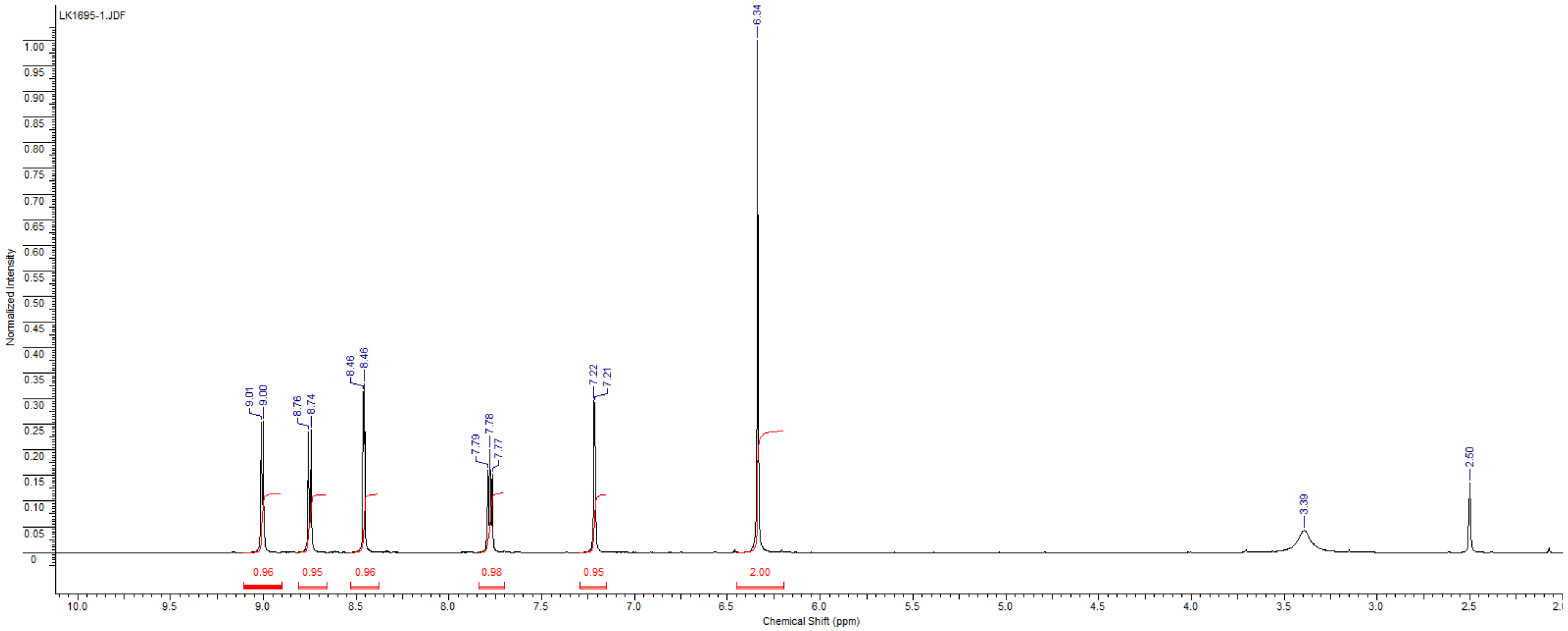

LK1695-1.JDF
Normalized Intensity
Chemical Shift (ppm)
9.01
9.00
8.76
8.74
8.46
8.46
7.79
7.78
7.77
7.22
7.21
6.34
3.39
2.50
0.96
0.95
0.96
0.98
0.95
2.00

3

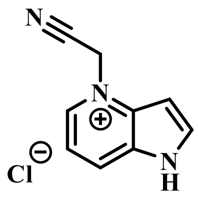
N
N
Cl
N
H

^{13}C

DMSO-d_6

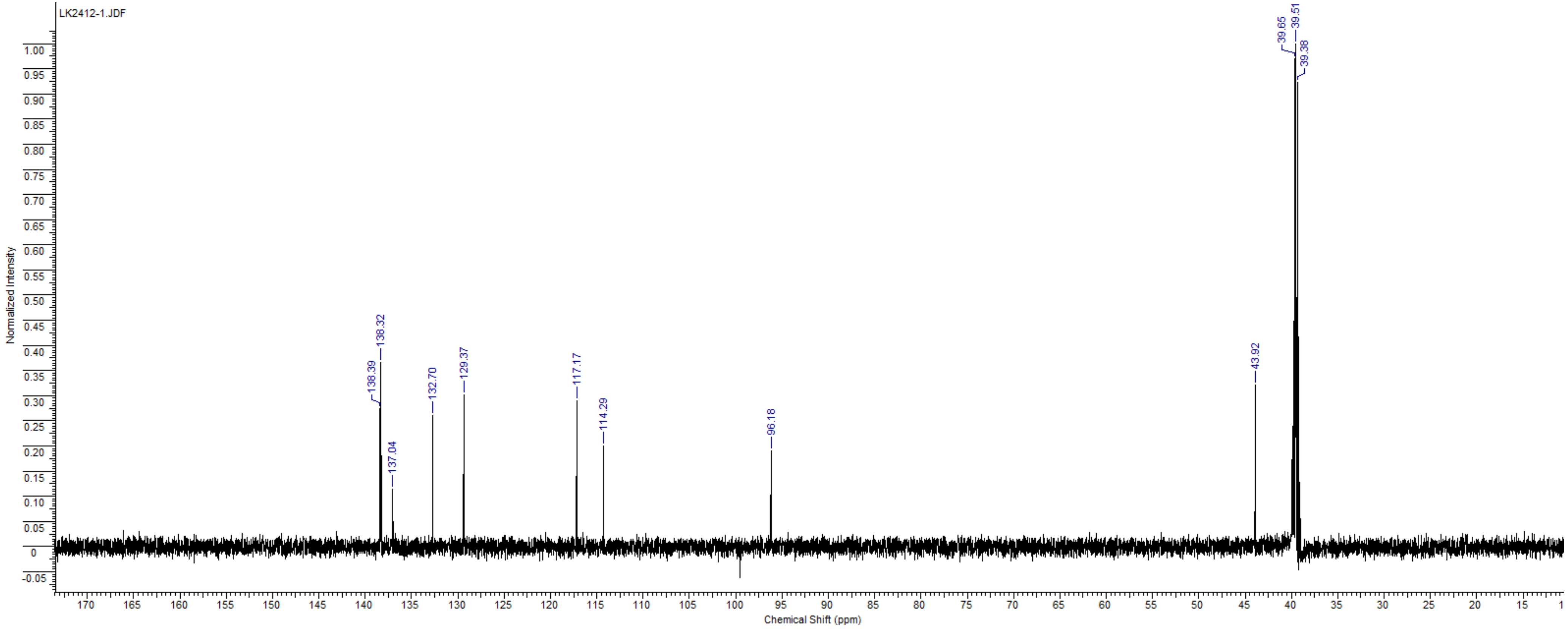
LK2412-1.JDF
39.65
39.51
39.38
138.39
138.32
137.04
132.70
129.37
117.17
114.29
96.18
43.92
Normalized Intensity
Chemical Shift (ppm)

8a

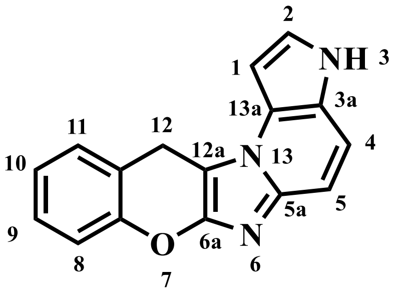

^{1}H

DMSO-d_6

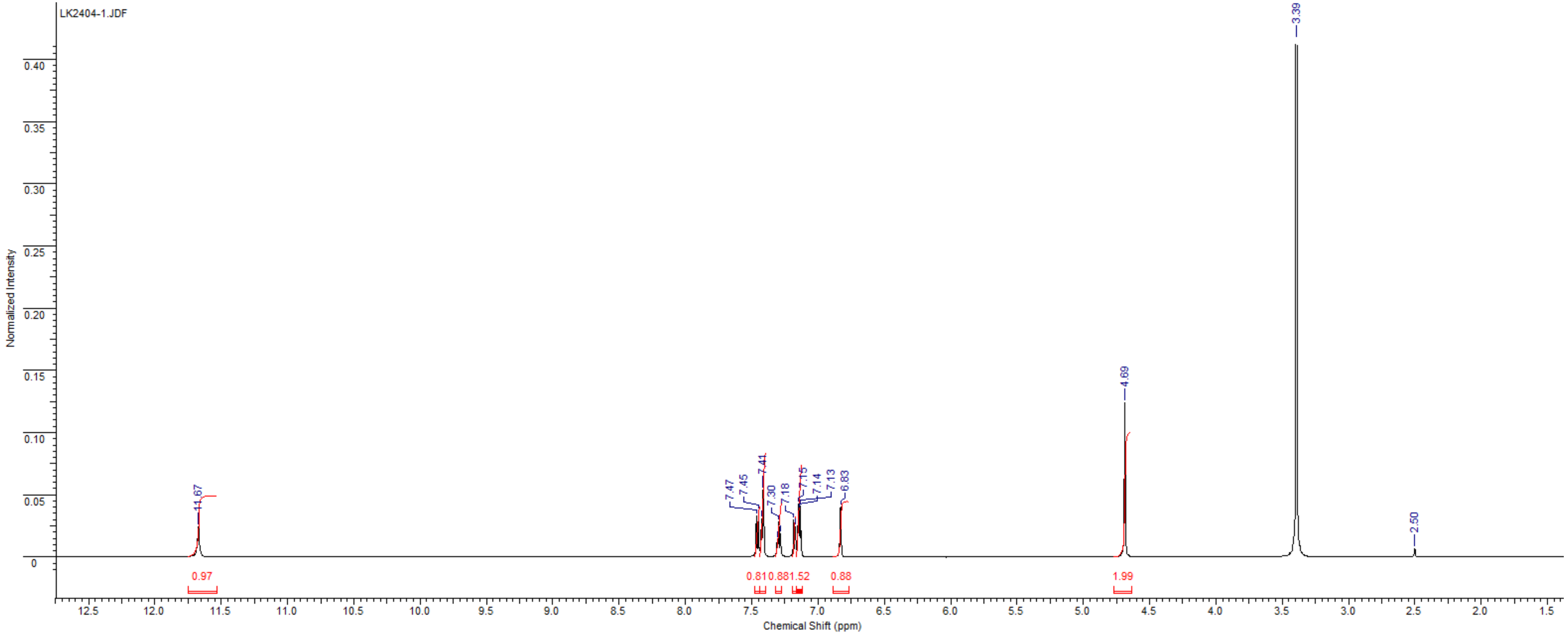
LK2404-1.JDF
Normalized Intensity
Chemical Shift (ppm)
11.67
7.47
7.45
7.41
7.30
7.18
7.15
7.14
7.13
6.83
4.69
3.39
2.50
0.97
0.81
0.88
1.52
0.88
1.99

8a

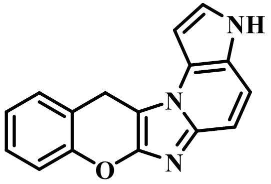

^{13}C

DMSO-d_6, 45°C

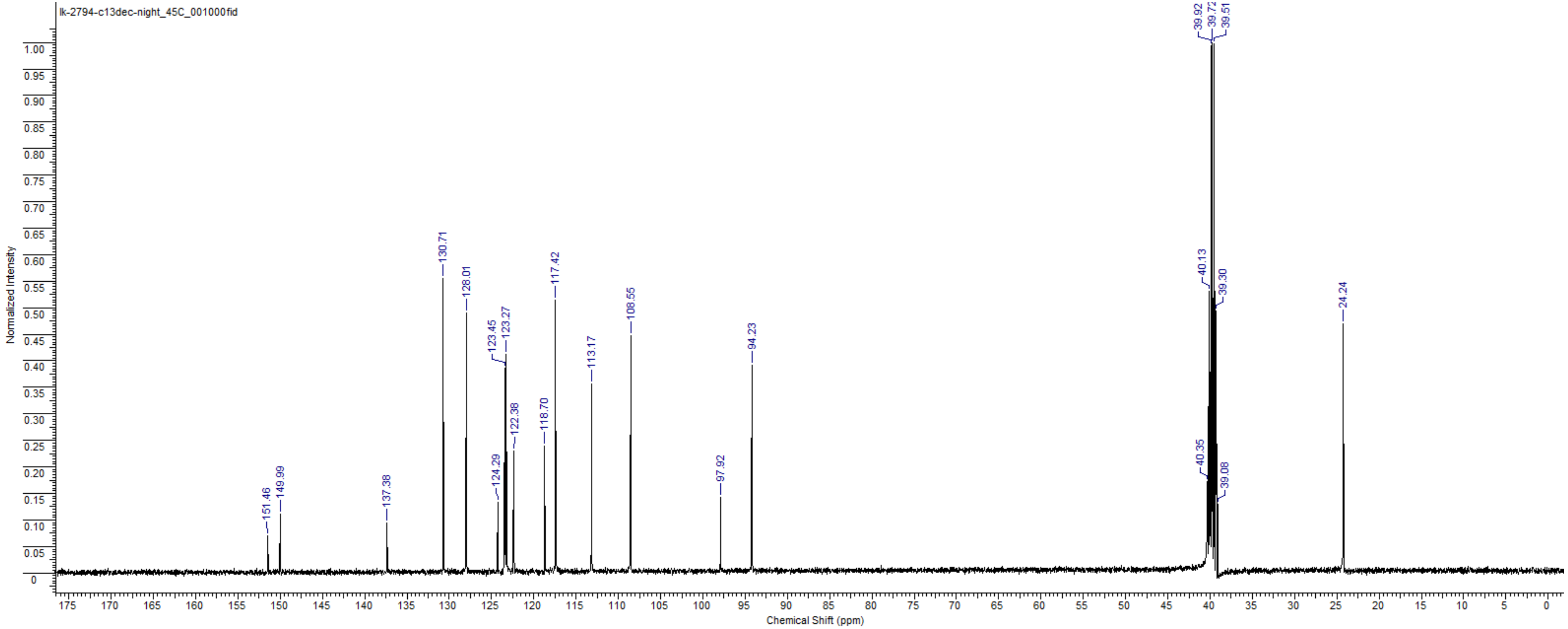

8b

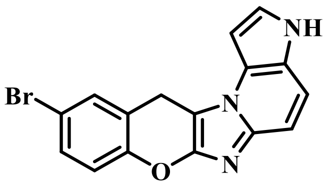
NH
Br
N
O
N

^{1}H

DMSO-d_6

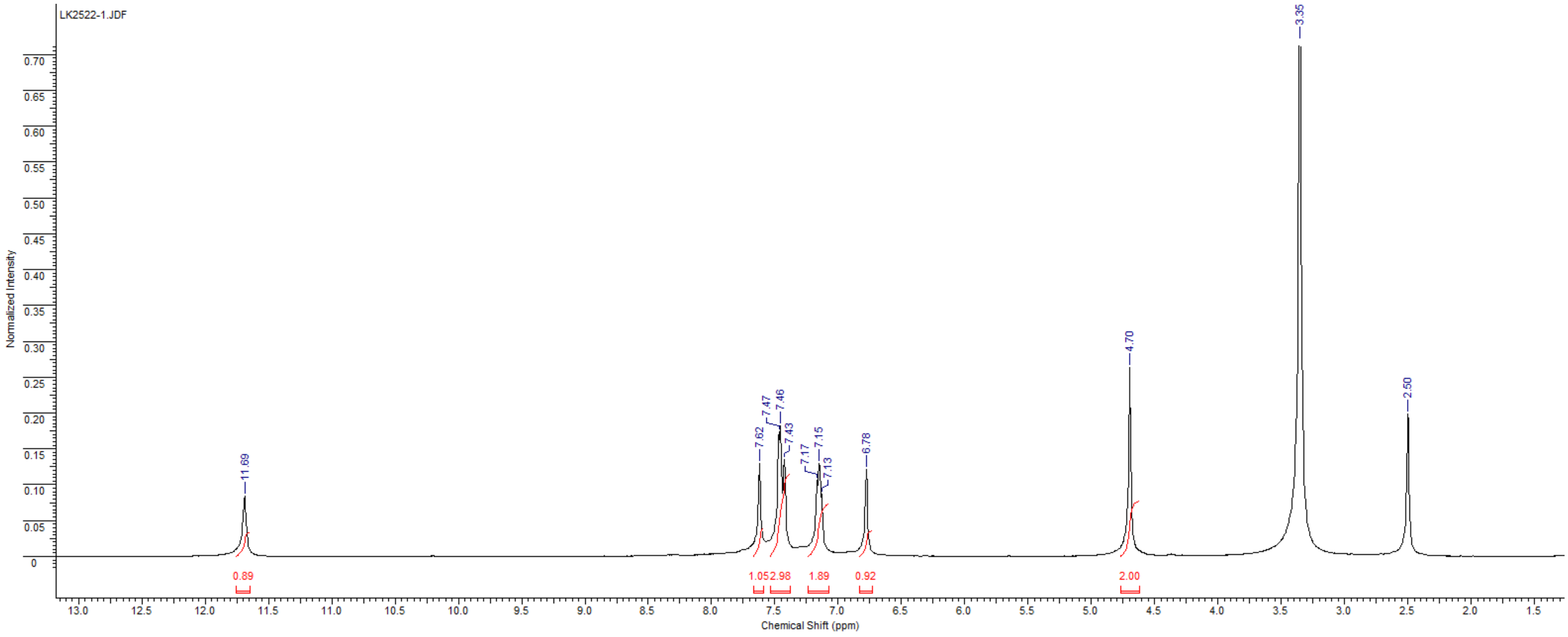
LK2522-1.JDF
Normalized Intensity
Chemical Shift (ppm)
11.69
7.62
7.47
7.46
7.43
7.17
7.15
7.13
6.78
4.70
3.35
2.50
0.89
1.05
2.98
1.89
0.92
2.00

8b

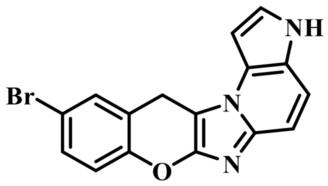

^{13}C

DMSO-d_6

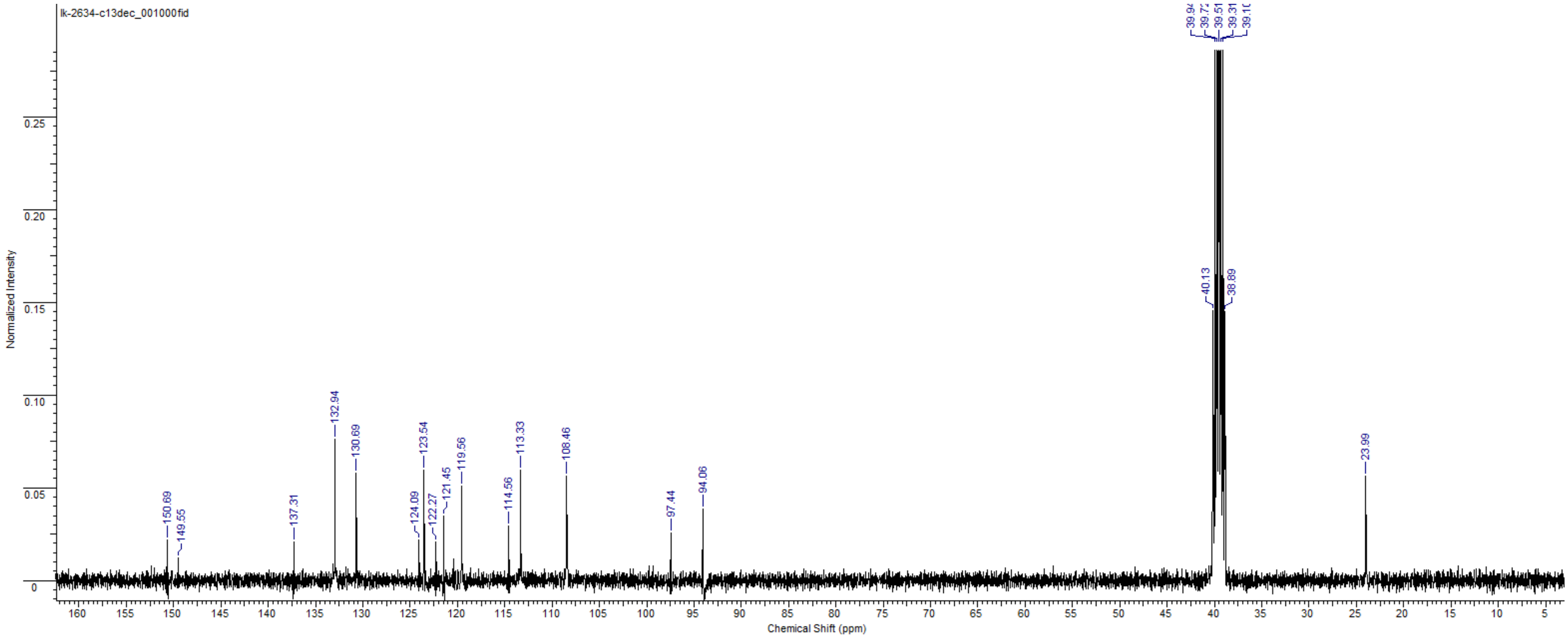

8c

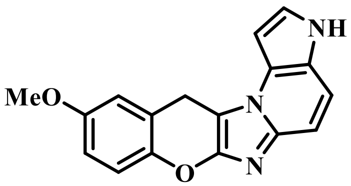
MeO
NH
N
N
O

^{1}H

DMSO-d_6

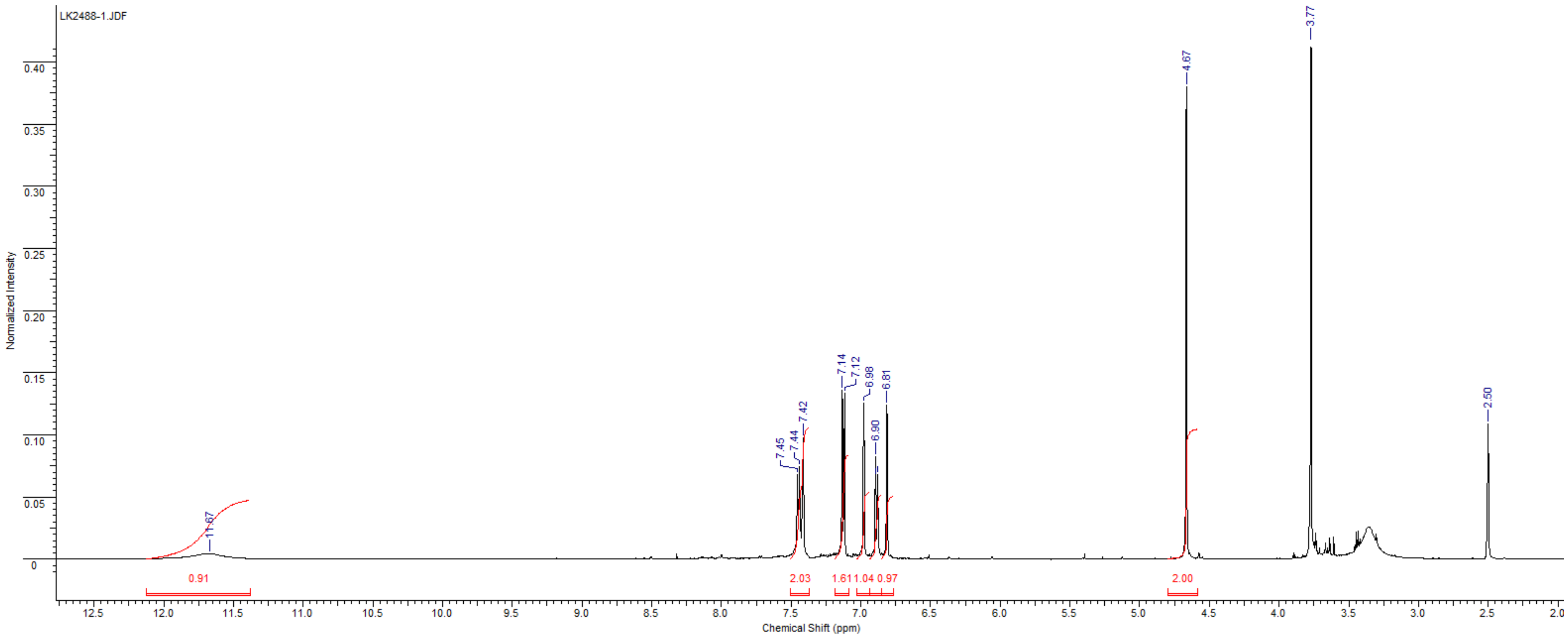
LK2488-1.JDF
Normalized Intensity
Chemical Shift (ppm)
11.67
7.45
7.44
7.42
7.14
7.12
6.98
6.90
6.81
4.67
3.77
2.50
0.91
2.03
1.61
1.04
0.97
2.00

8c

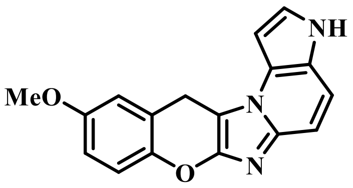

^{13}C

DMSO-d_6

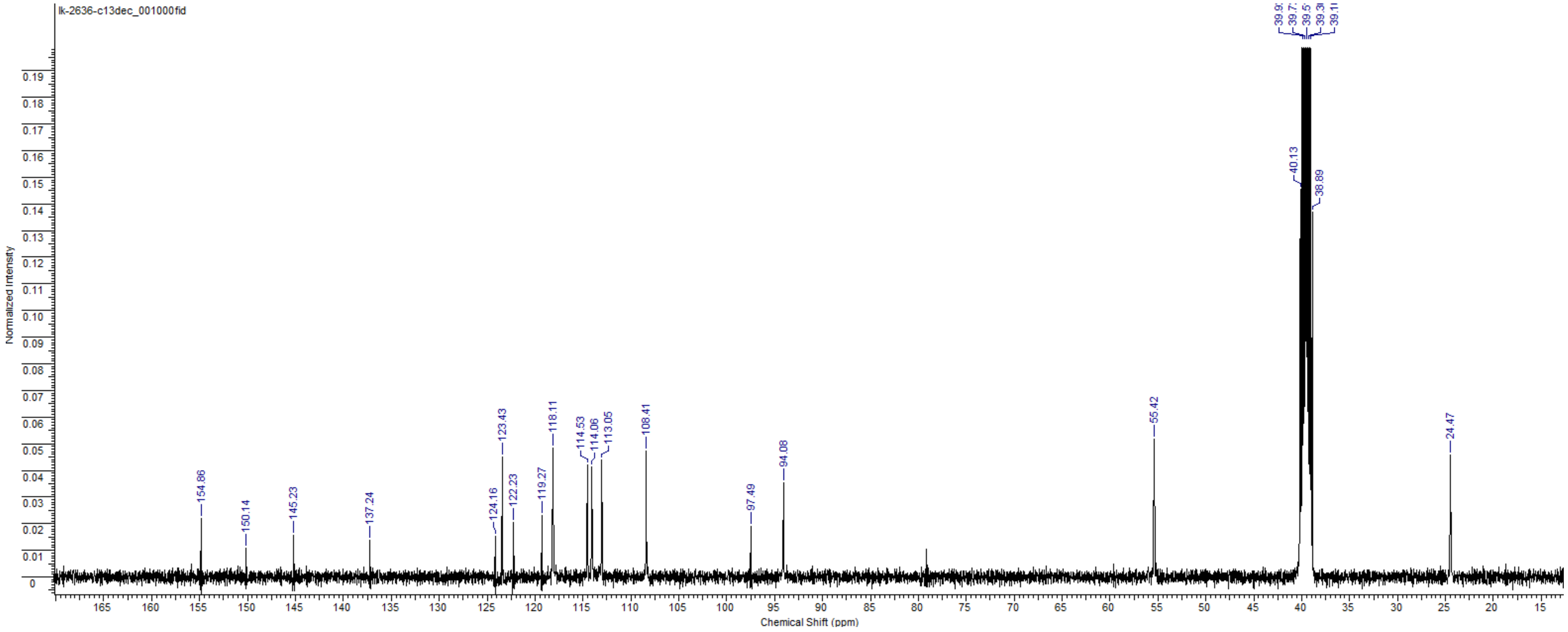

8d

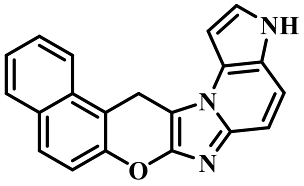

NH
N
N
O

^{1}H

DMSO-d_6

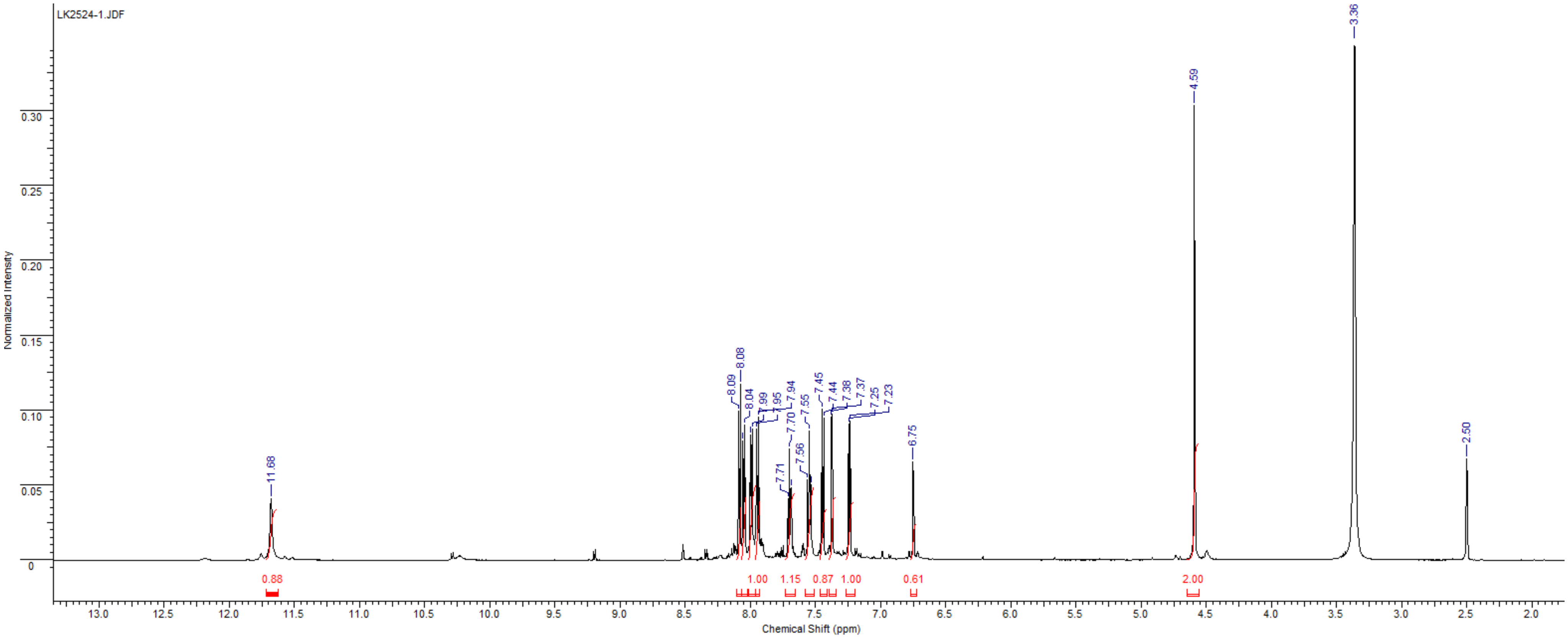

LK2524-1.JDF
Normalized Intensity
Chemical Shift (ppm)
11.68
8.09
8.08
8.04
7.99
7.95
7.94
7.71
7.70
7.56
7.55
7.45
7.44
7.38
7.37
7.25
7.23
6.75
4.59
3.36
2.50
0.88
1.00
1.15
0.87
1.00
0.61
2.00

8d

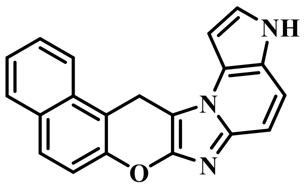

^{13}C

DMSO-d_6

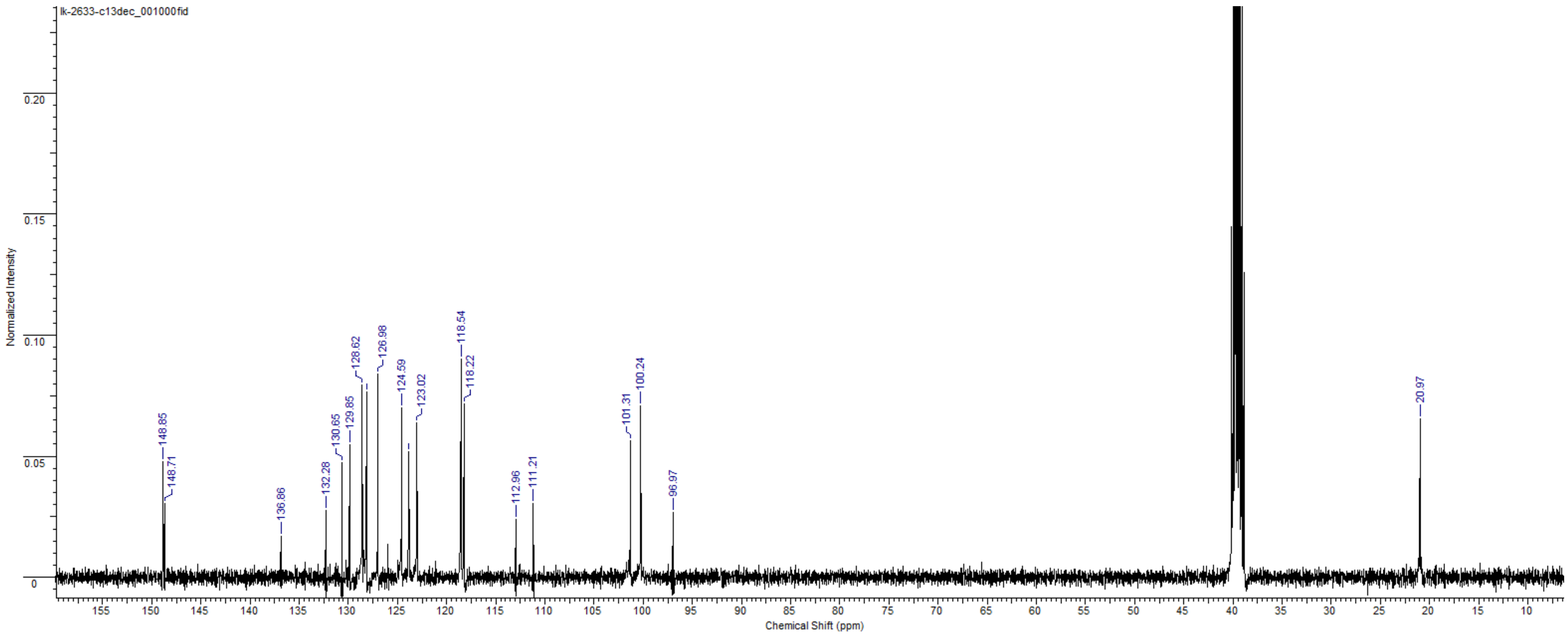

General experimental details.

Spectrometers models: 400 — «Bruker AMX-III 400», 300 — «Bruker AVANCE III HD 300».

^{1}H, ^{13}C and ^{15}N NMR spectra were recorded on a 400 MHz (400.1, 100.6 and 40.6 MHz, respectively) and 300 MHz (300.1, 75.5 and 30.4 MHz, respectively) spectrometers in $CDCl_3$ and DMSO-D_6 containing 0.05% Me_4Si as the internal standard. Determinations of structures and stereochemistry of obtained compounds and assignments of ^{1}H, ^{13}C and ^{15}N signals were made with the aid of 2D COSY, TOCSY, NOESY, HSQC, edited-HSQC, HMBC, long-range HMBC, and ^{15}N-HMBC. All ^{15}N NMR spectra were acquired on natural abundance os ^{15}N-isotope using 2D pulse sequences in inverse mode.

Fig. 1. Key cross-peaks and correlations in 2D NMR spectra for structure assignments indicated by arrows (blue – NOE; red – ^{1}H,^{13}C-HMBC optimized on long-range coupling; green – ^{1}H,^{15}N-HMBC).

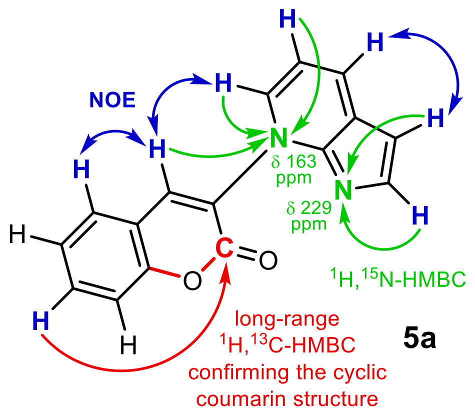

5a

The structure of the compounds obtained were determined by ^{1}H, ^{13}C and ^{15}N NMR spectroscopy using 2D COSY, TOCSY, NOESY, HSQC, edited-HSQC, HMBC, long-range HMBC, and ^{15}N-HMBC spectra (for details, see Supporting Information). Key cross-peaks and correlations in 2D NMR spectra are shown on Fig. 2 by color arrows for compound **5a** as an example. Compound **5a** contains in its structure the cyclic coumarine skeleton and 7*H*-pyrrolo[2,3-*b*]pyridin-7-yl fragment. In ^{1}H NMR spectra in DMSO signals of NH and OH are absent. ^{1}H,^{13}C-HMBC NMR spectra optimized on long-range coupling show the strong ^{1}H-^{13}C correlation from aromatic ring to COO carbon atom and confirm the cyclic coumarin structure. Inverse 2D ^{15}N correlation NMR spectra on natural abundance of ^{15}N-isotope allow to see the both nitrogen atoms and its environment. Structure of **5a** contains 7*H*-pyrrolo[2,3-*b*]pyridin-7-yl fragment with pyrrole-type nitrogen in six-member ring (163 ppm in ^{15}N chem. shift scale) and piridine-type nitrogen in five-member ring (229 ppm). Summarize, a set of 1D and 2D NMR data allows to uniquely determine the structures of obtained products.

LK-2162-COSY

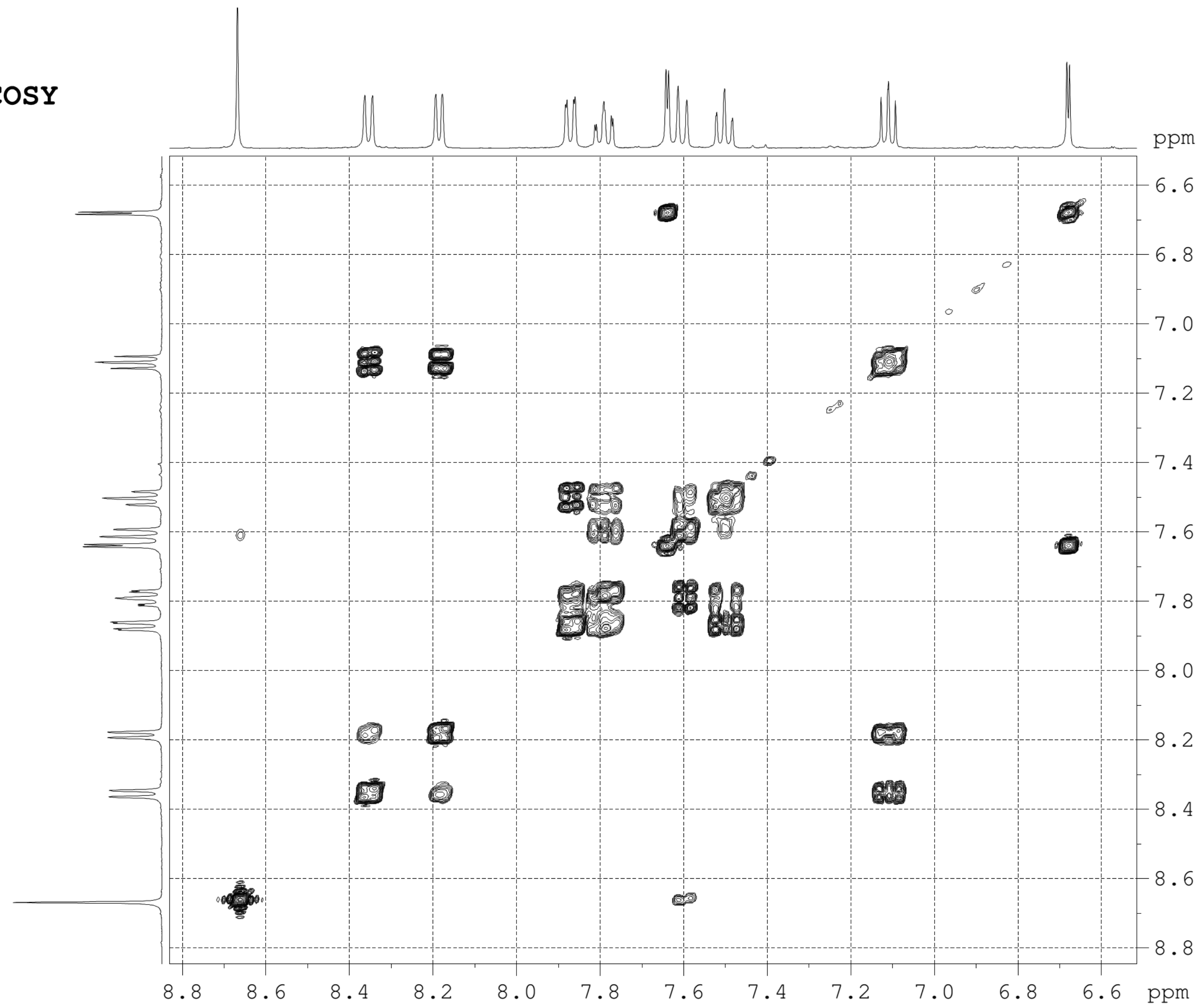

ppm
6.6
6.8
7.0
7.2
7.4
7.6
7.8
8.0
8.2
8.4
8.6
8.8
8.8 8.6 8.4 8.2 8.0 7.8 7.6 7.4 7.2 7.0 6.8 6.6 ppm

LK-2162-NOESY

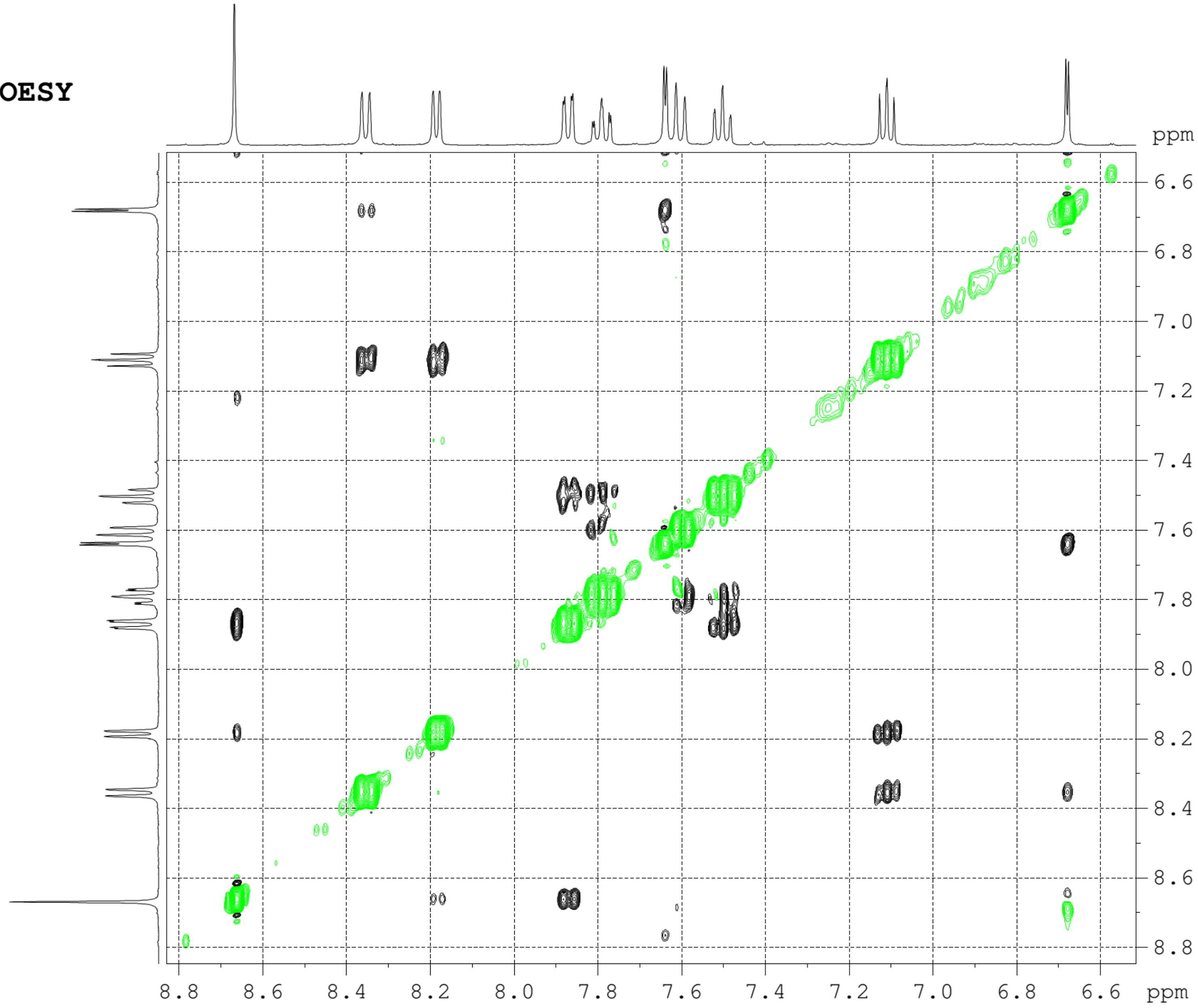

LK-2162-HSQC

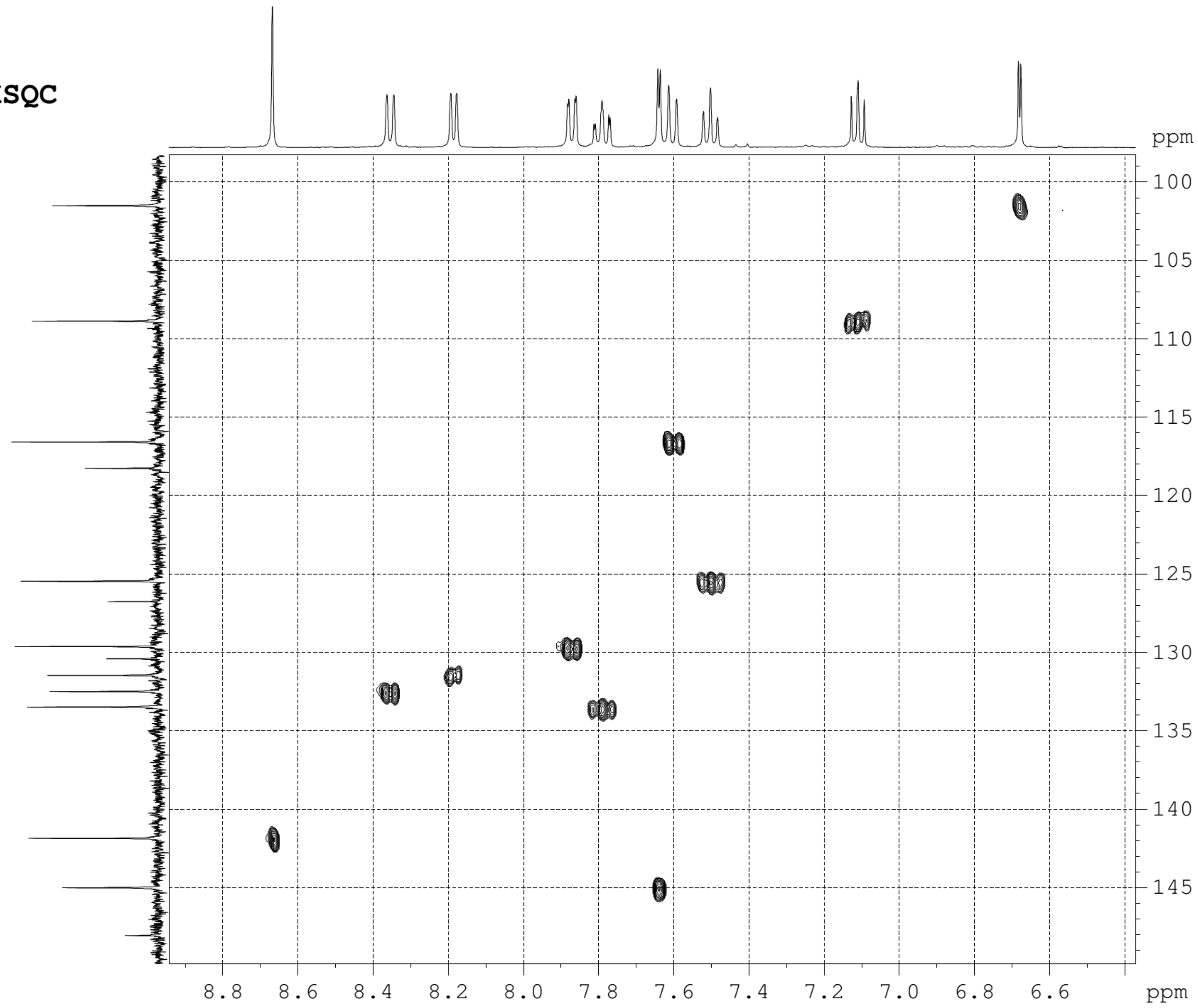
ppm
100
105
110
115
120
125
130
135
140
145
8.8
8.6
8.4
8.2
8.0
7.8
7.6
7.4
7.2
7.0
6.8
6.6
ppm

LK-2162-HMBC

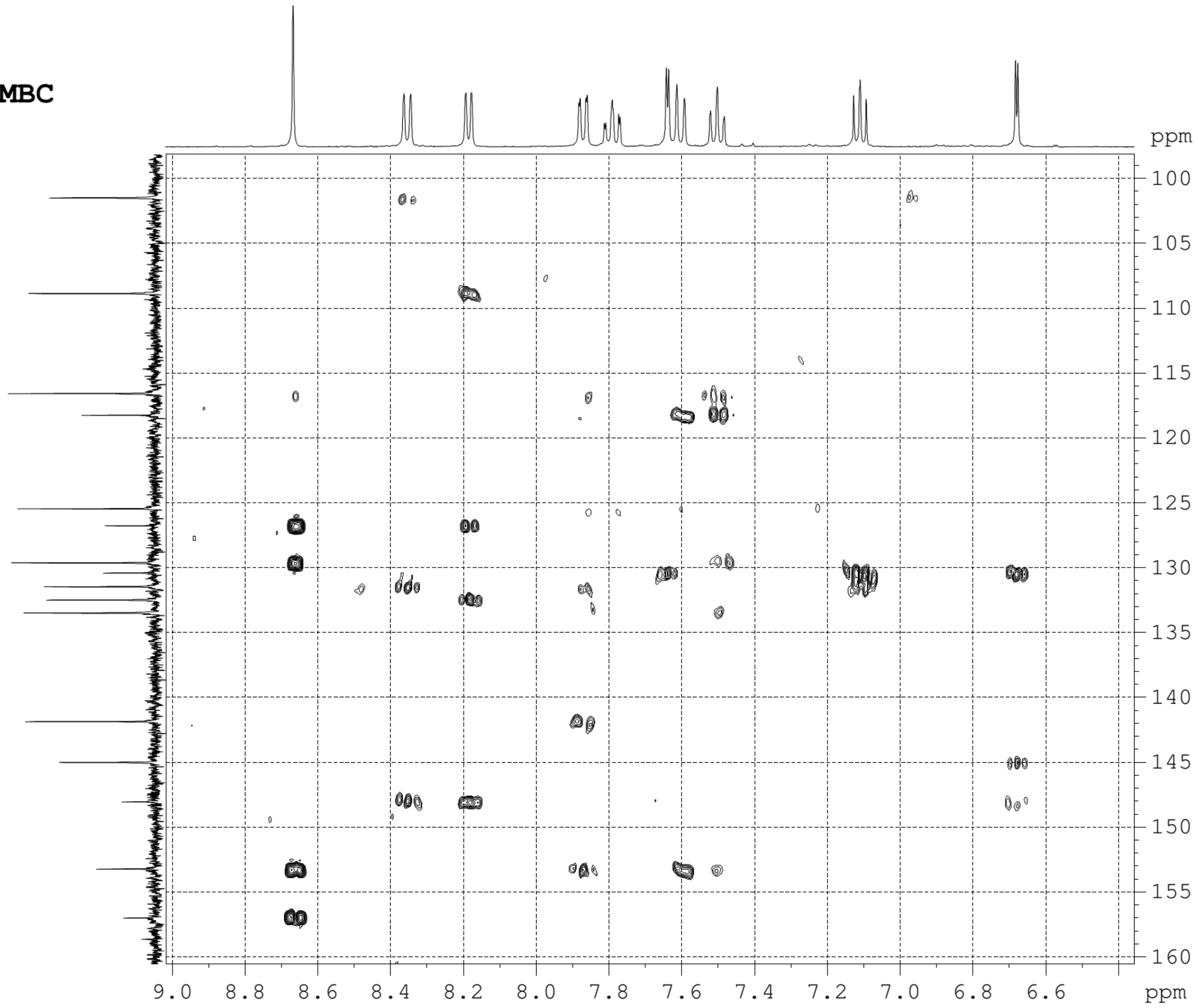
ppm
100
105
110
115
120
125
130
135
140
145
150
155
160
9.0 8.8 8.6 8.4 8.2 8.0 7.8 7.6 7.4 7.2 7.0 6.8 6.6
ppm

LK-2162-LR-HMBC

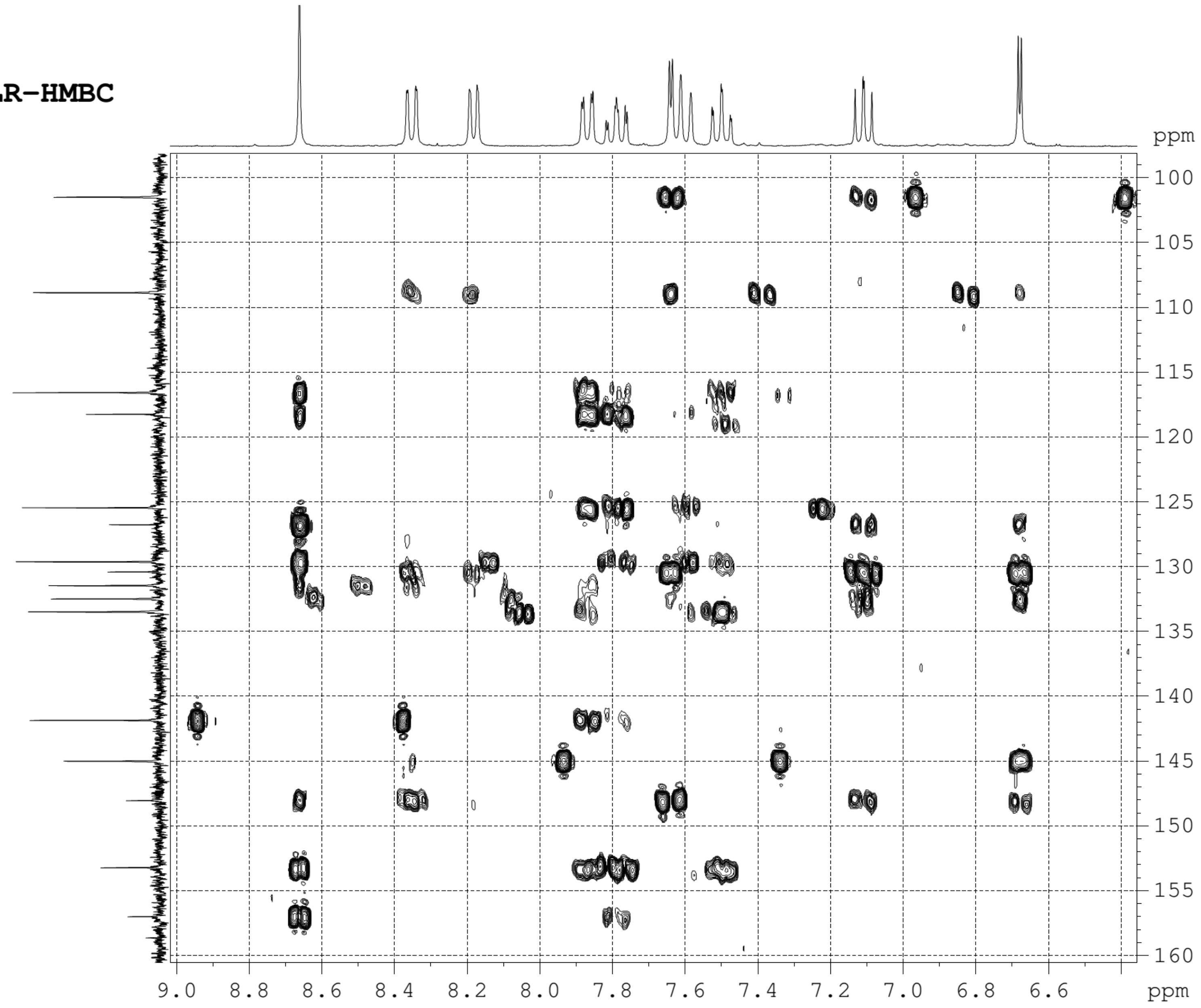

LK-2162-N15-HMBC

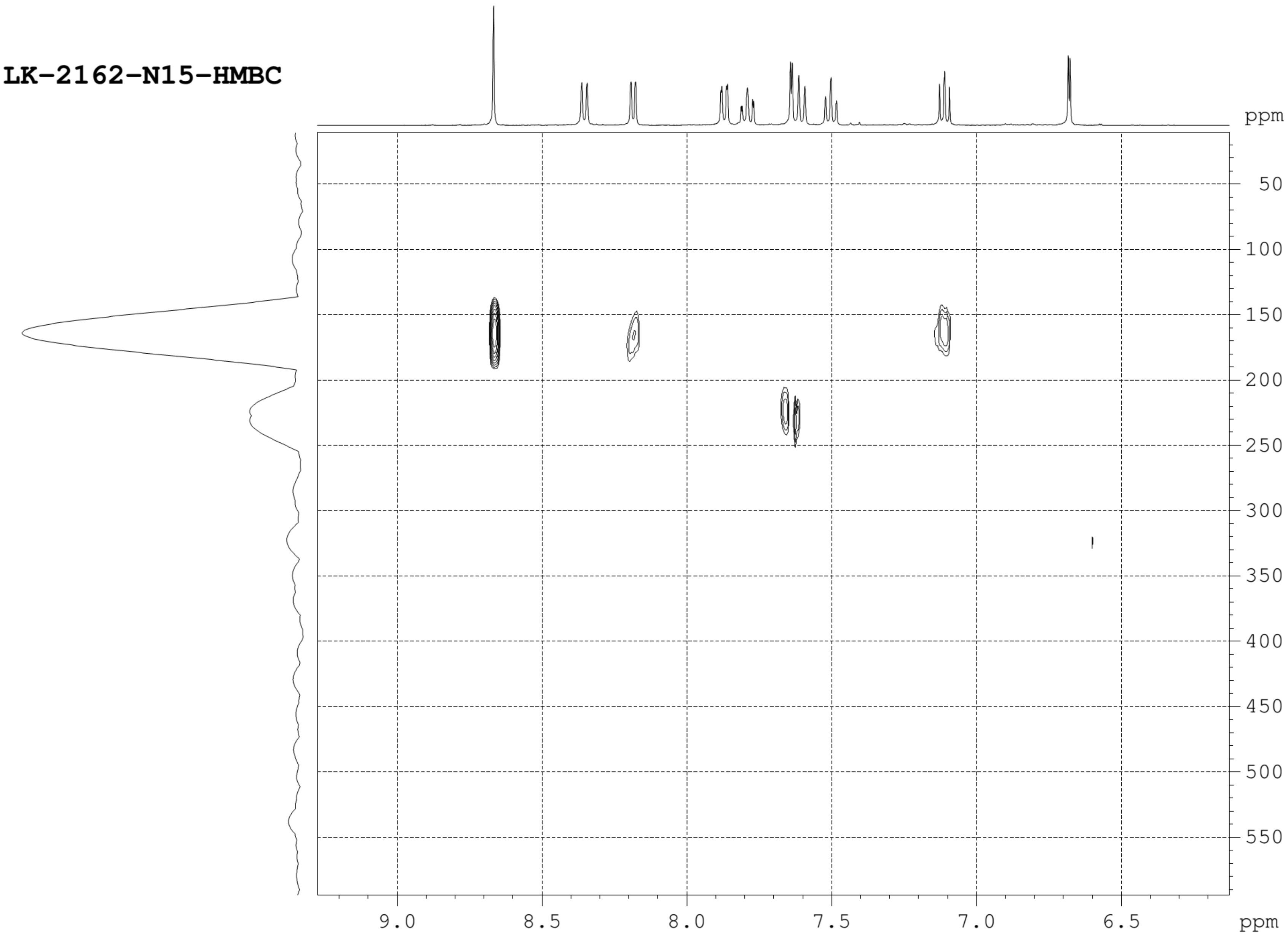

LK-2162-N15-HMBC

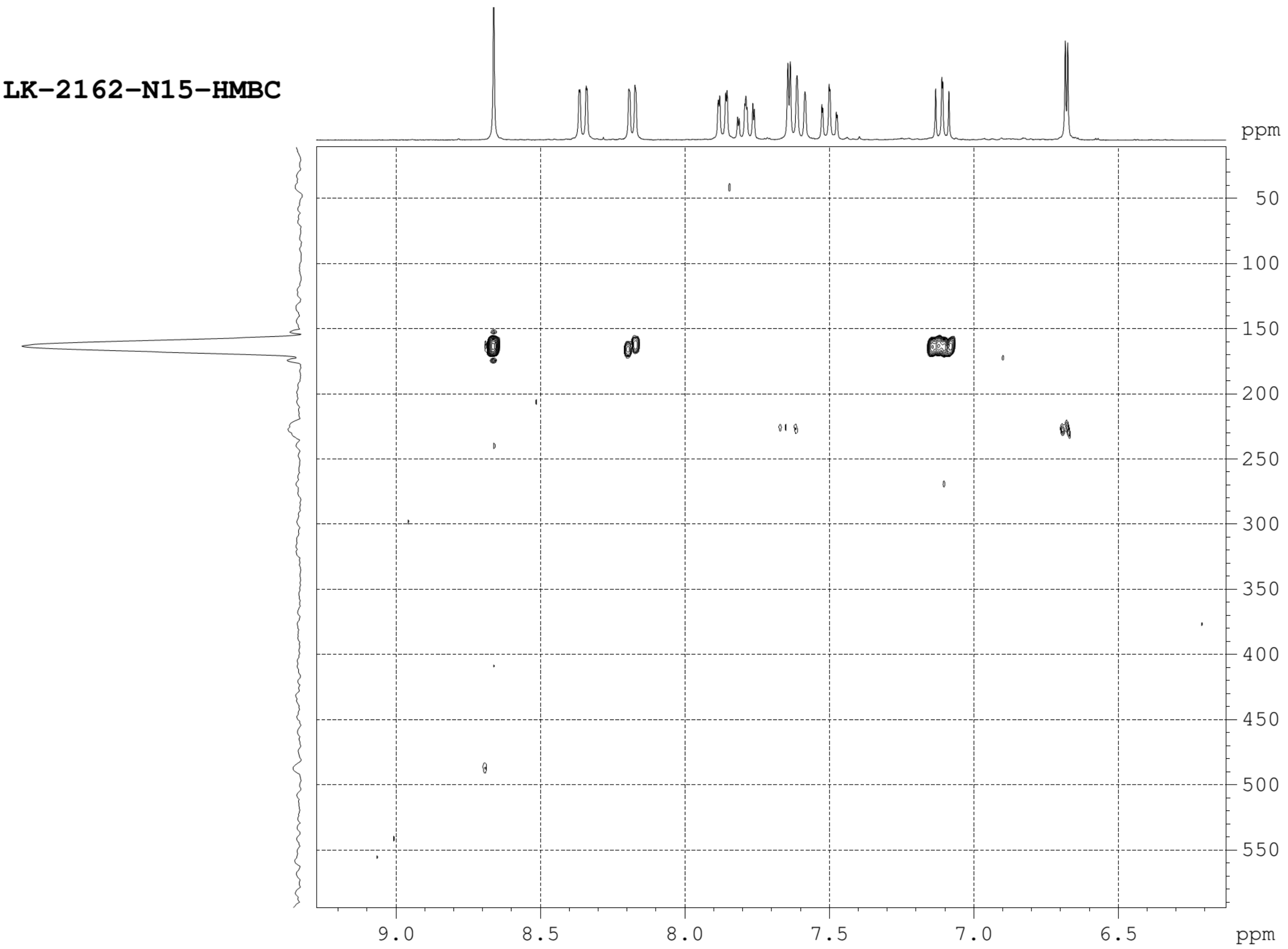

5a

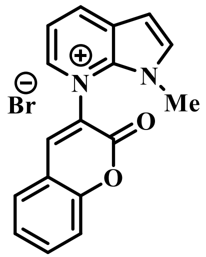
Br
N
N
Me
O
O

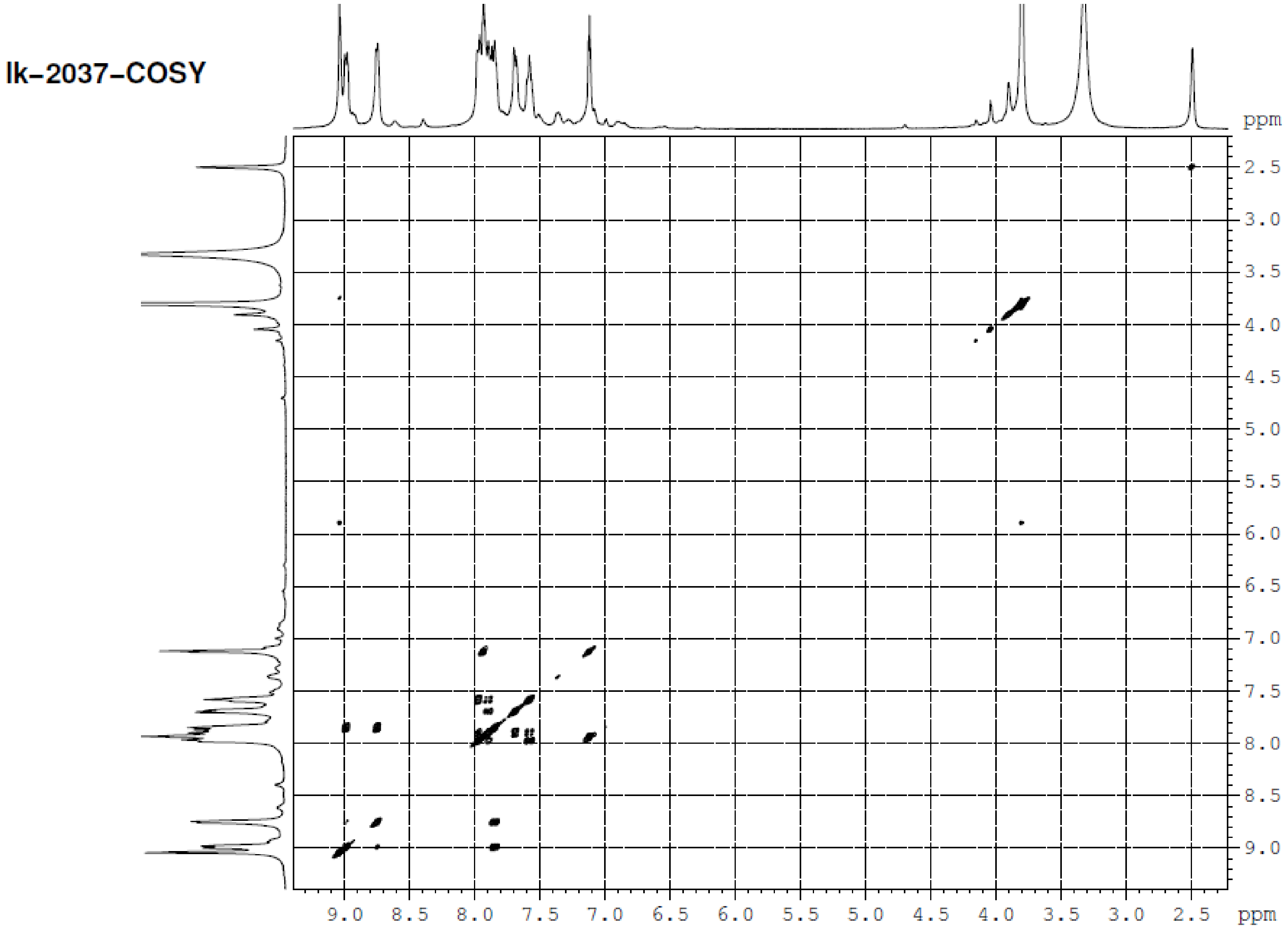
lk-2037-COSY
ppm
2.5
3.0
3.5
4.0
4.5
5.0
5.5
6.0
6.5
7.0
7.5
8.0
8.5
9.0
9.0 8.5 8.0 7.5 7.0 6.5 6.0 5.5 5.0 4.5 4.0 3.5 3.0 2.5 ppm

lk−2037−HSQC

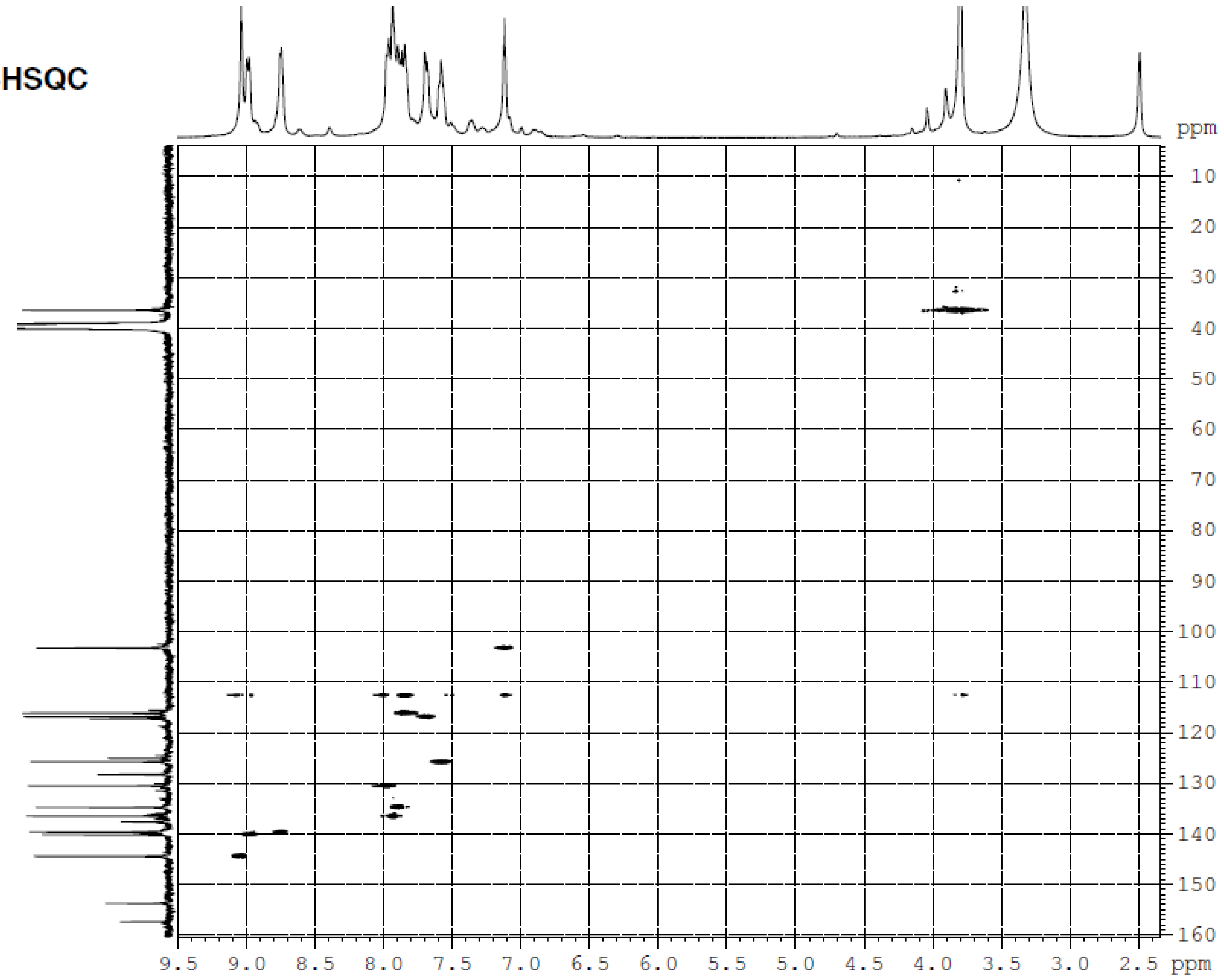
ppm
10
20
30
40
50
60
70
80
90
100
110
120
130
140
150
160
9.5 9.0 8.5 8.0 7.5 7.0 6.5 6.0 5.5 5.0 4.5 4.0 3.5 3.0 2.5 ppm

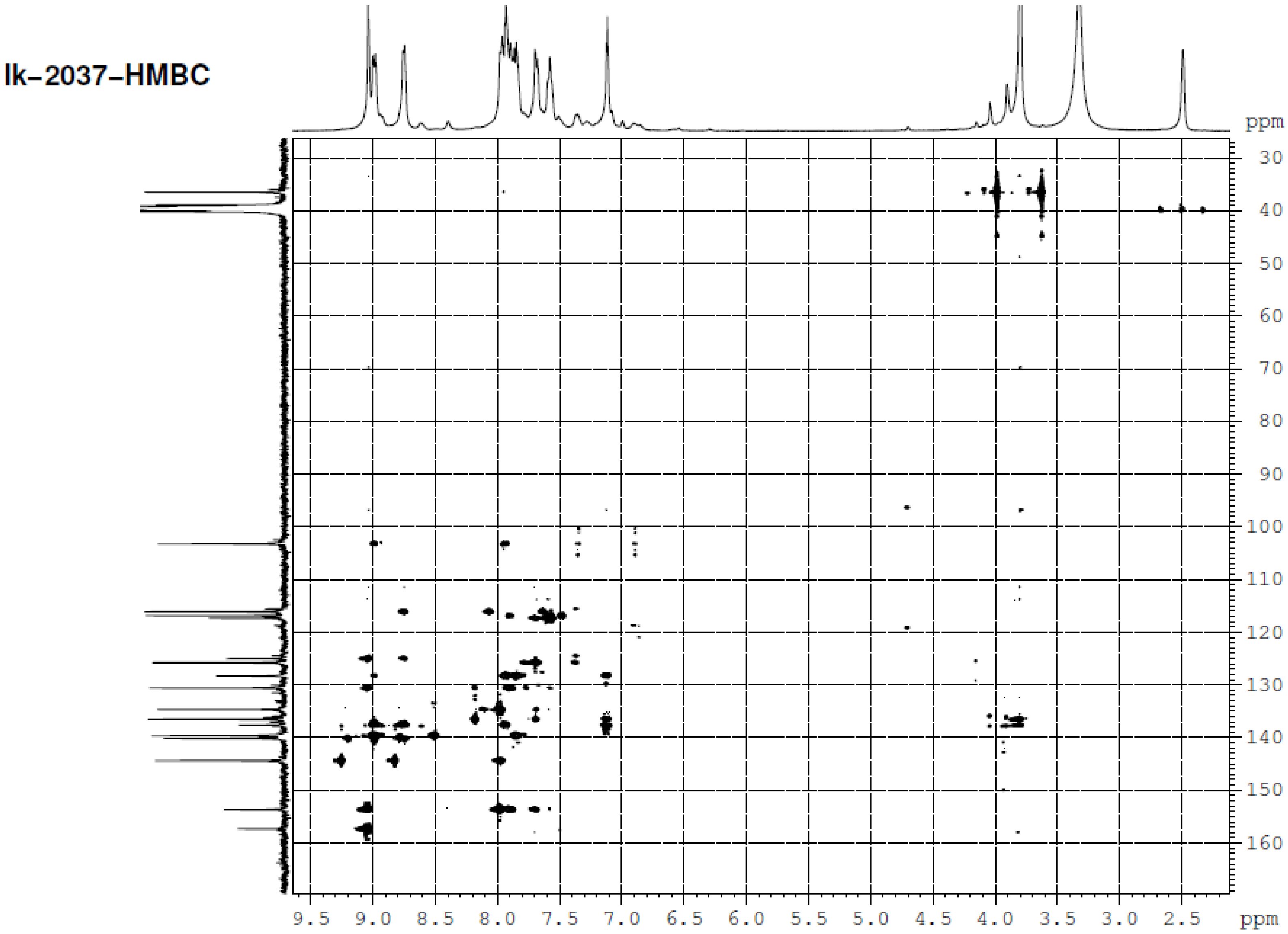
lk-2037-HMBC
ppm
30
40
50
60
70
80
90
100
110
120
130
140
150
160
9.5 9.0 8.5 8.0 7.5 7.0 6.5 6.0 5.5 5.0 4.5 4.0 3.5 3.0 2.5
ppm

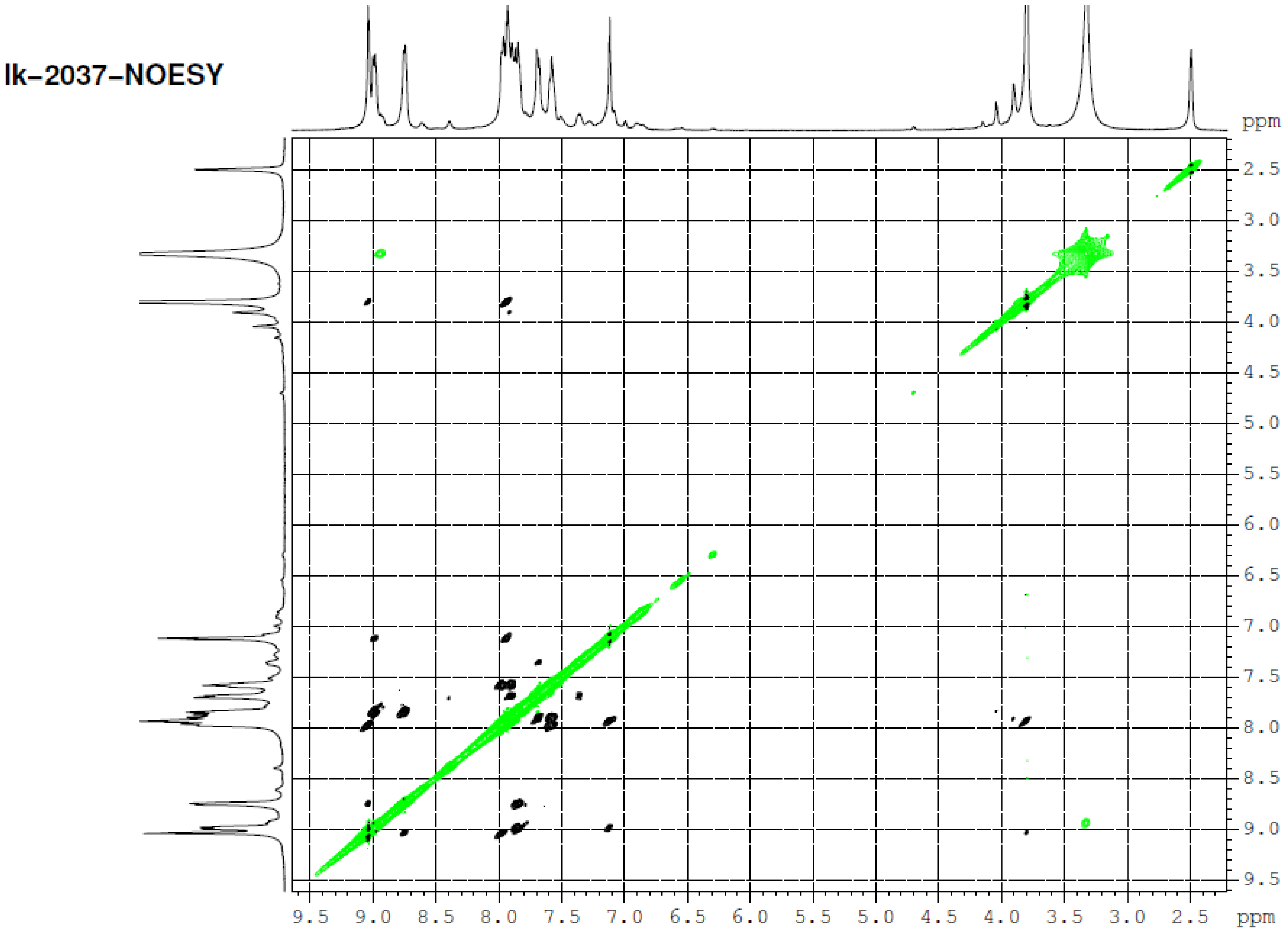
lk–2037–NOESY
ppm
2.5
3.0
3.5
4.0
4.5
5.0
5.5
6.0
6.5
7.0
7.5
8.0
8.5
9.0
9.5
9.5 9.0 8.5 8.0 7.5 7.0 6.5 6.0 5.5 5.0 4.5 4.0 3.5 3.0 2.5 ppm

6c

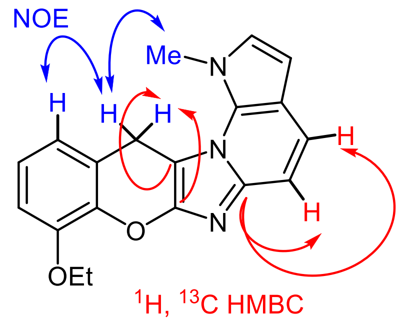

6c

Key cross-peaks and correlations in 2D NMR spectra for structure assignments indicated by arrows (blue – NOE; red – $^{1}H,^{13}C$-HMBC).

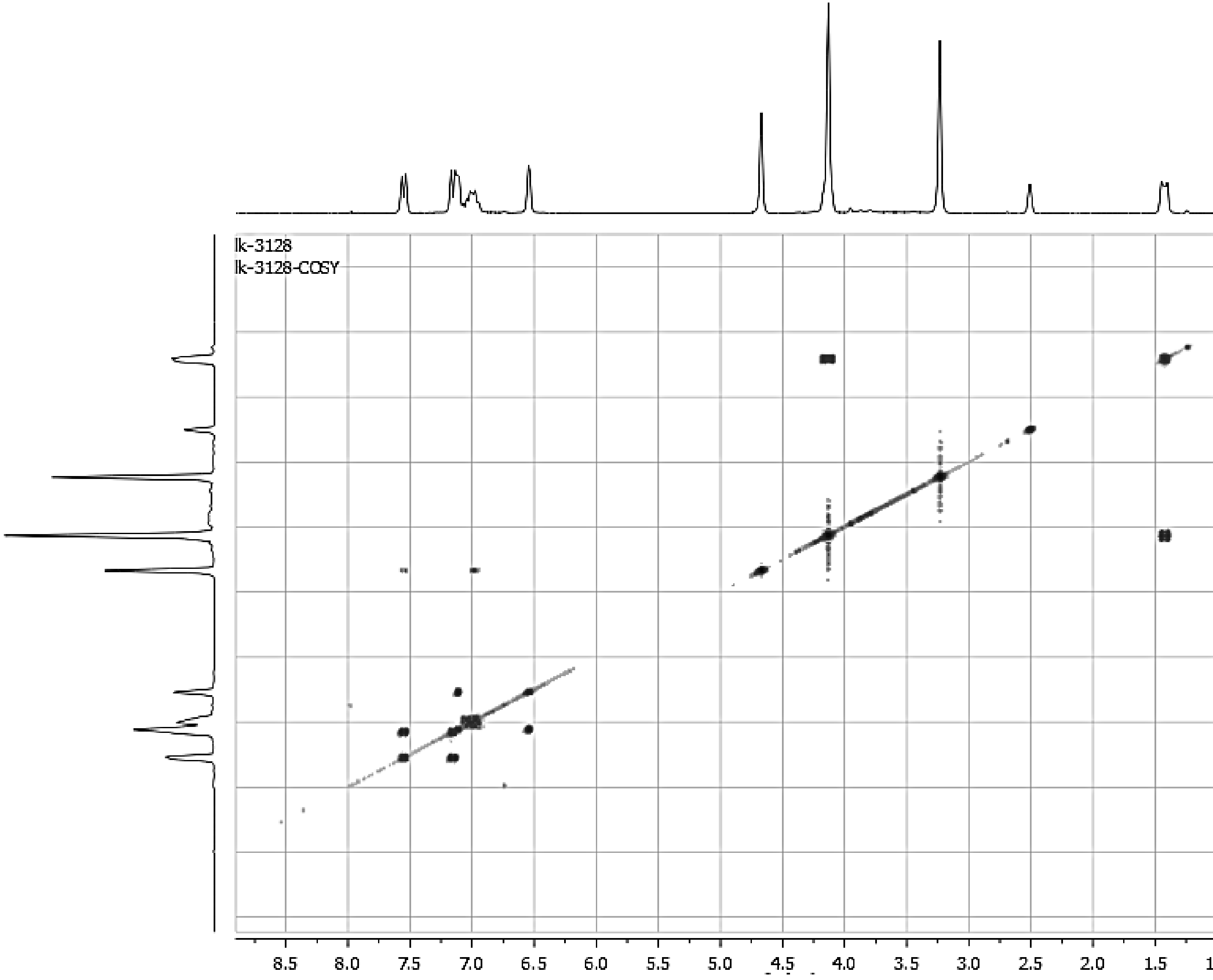

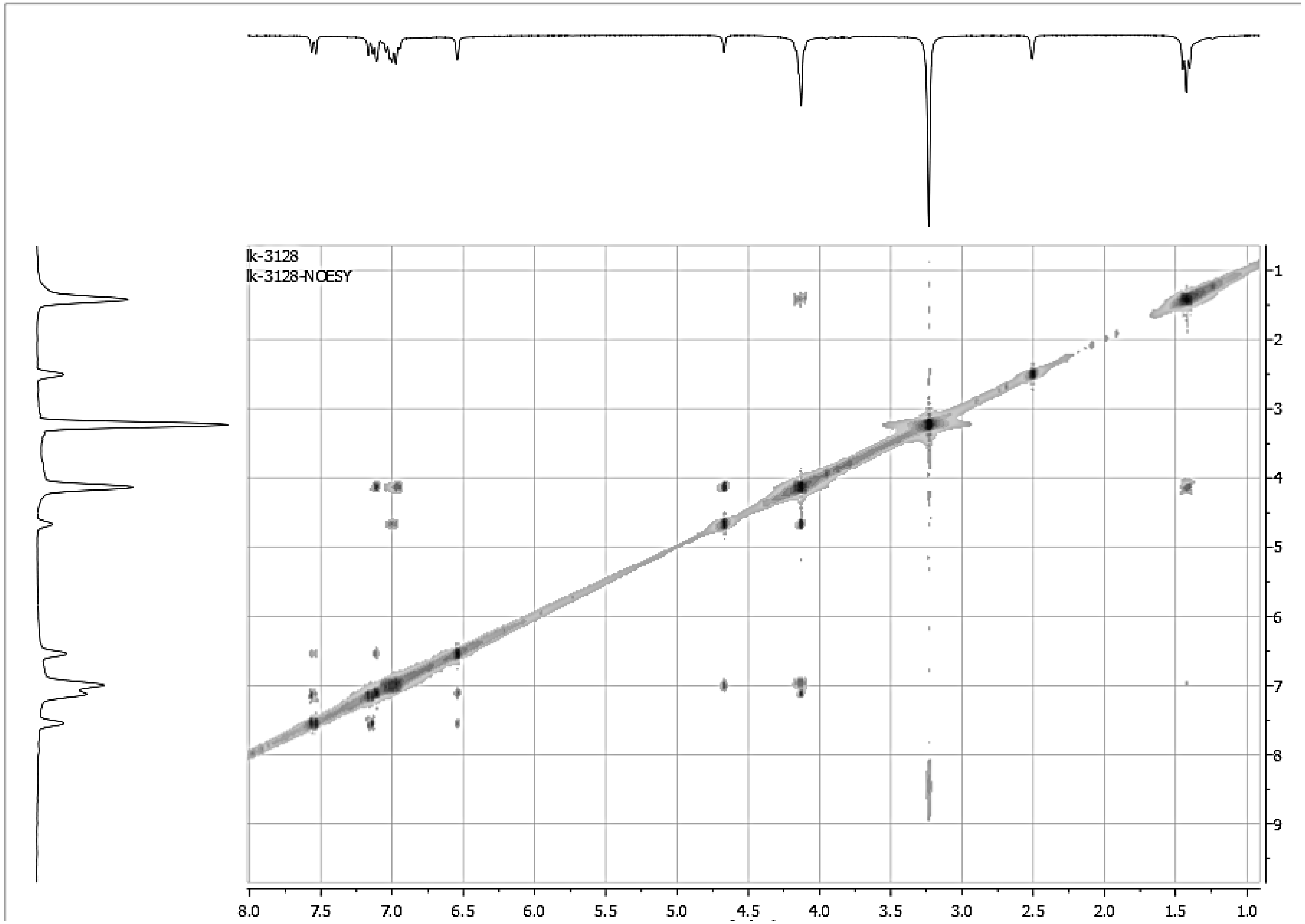
lk-3128
lk-3128-NOESY
8.0 7.5 7.0 6.5 6.0 5.5 5.0 4.5 4.0 3.5 3.0 2.5 2.0 1.5 1.0
1 2 3 4 5 6 7 8 9

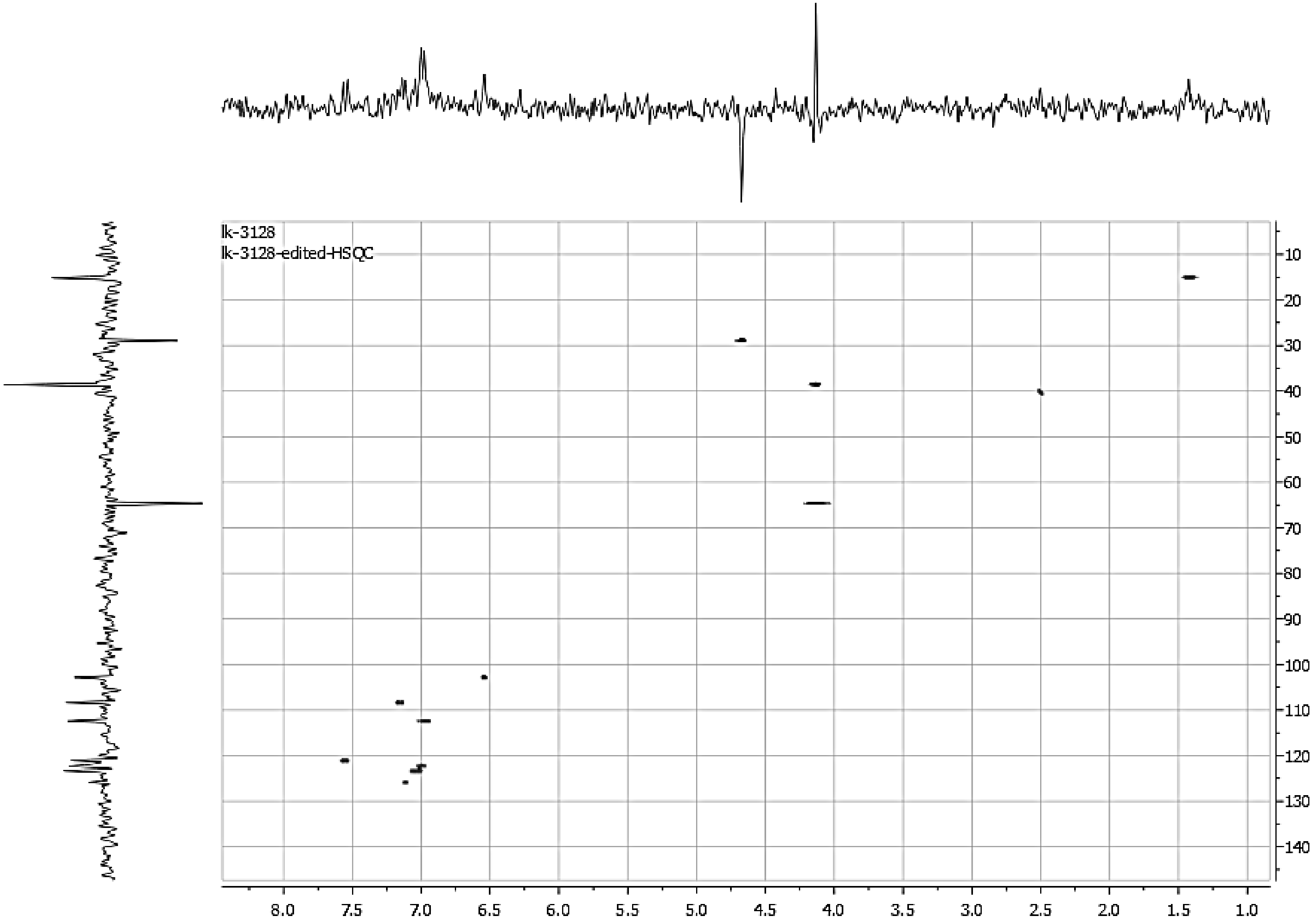
lk-3128
lk-3128-edited-HSQC
8.0 7.5 7.0 6.5 6.0 5.5 5.0 4.5 4.0 3.5 3.0 2.5 2.0 1.5 1.0
10 20 30 40 50 60 70 80 90 100 110 120 130 140

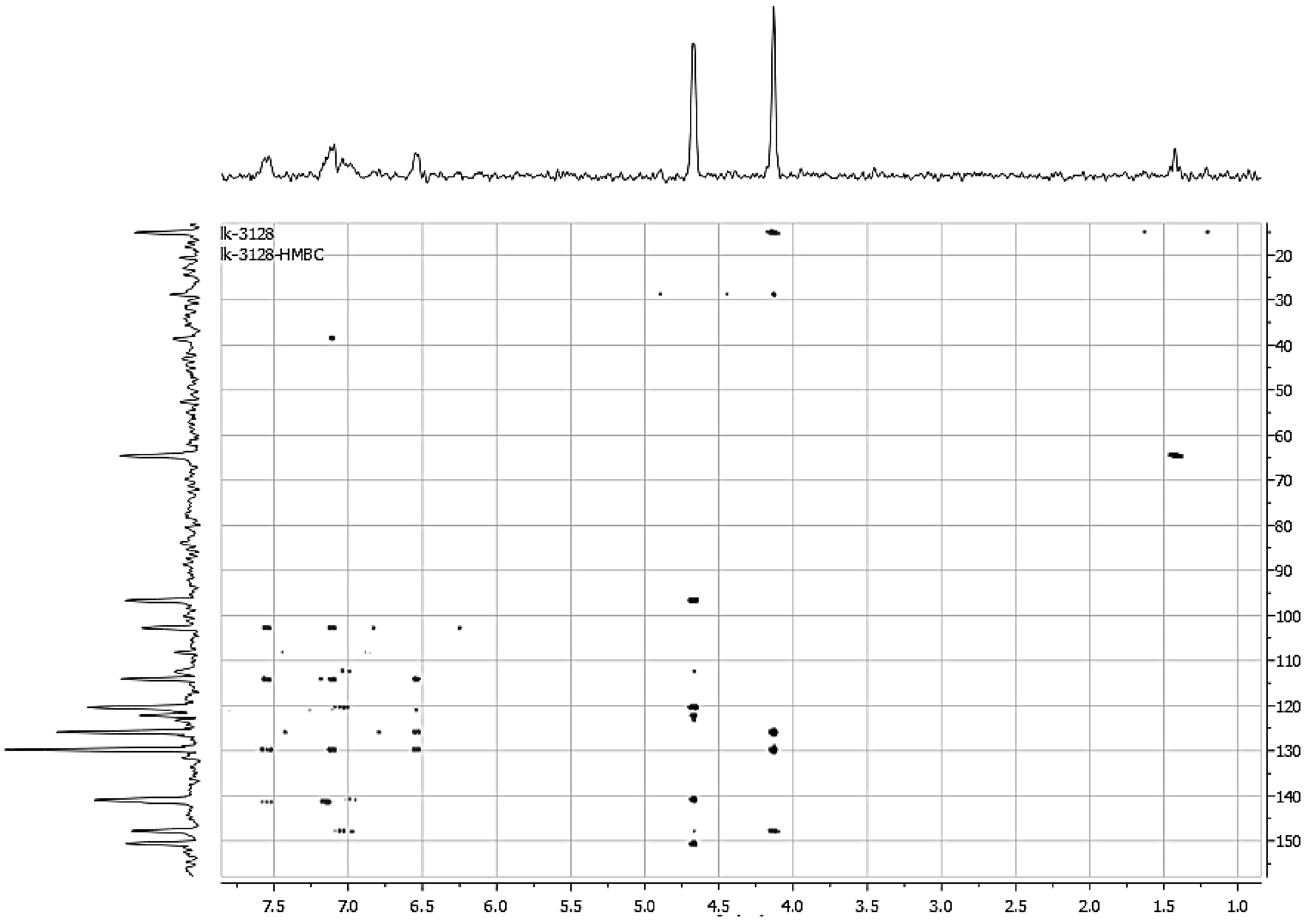
lk-3128
lk-3128-HMBC
7.5 7.0 6.5 6.0 5.5 5.0 4.5 4.0 3.5 3.0 2.5 2.0 1.5 1.0
20 30 40 50 60 70 80 90 100 110 120 130 140 150

7a

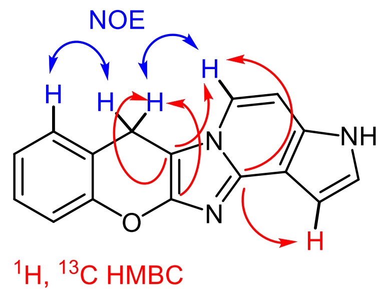

7a

Key cross-peaks and correlations in 2D NMR spectra for structure assignments indicated by arrows (blue – NOE; red – ^{1}H,^{13}C-HMBC)

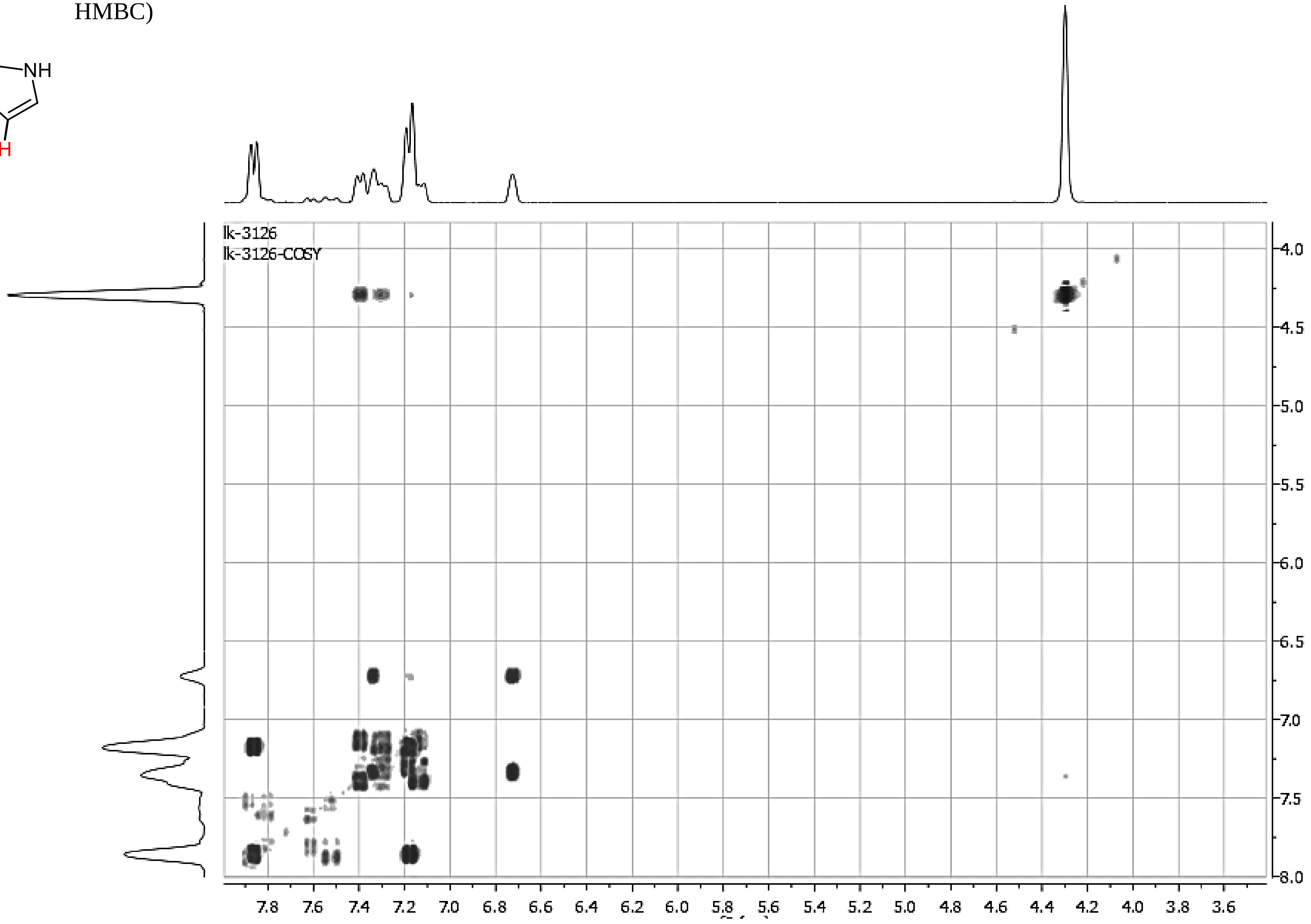

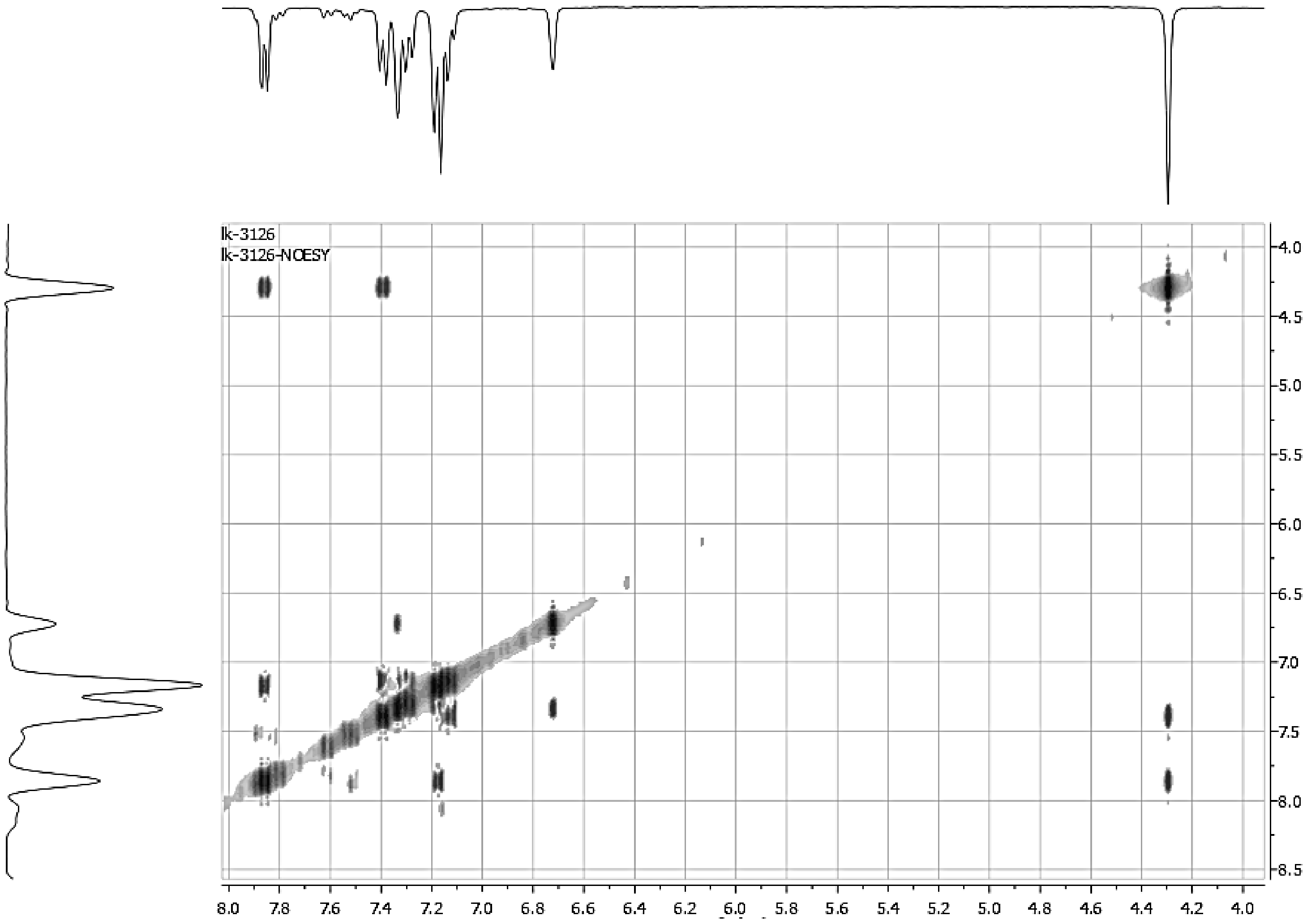
lk-3126
lk-3126-NOESY
4.0
4.5
5.0
5.5
6.0
6.5
7.0
7.5
8.0
8.5
8.0 7.8 7.6 7.4 7.2 7.0 6.8 6.6 6.4 6.2 6.0 5.8 5.6 5.4 5.2 5.0 4.8 4.6 4.4 4.2 4.0

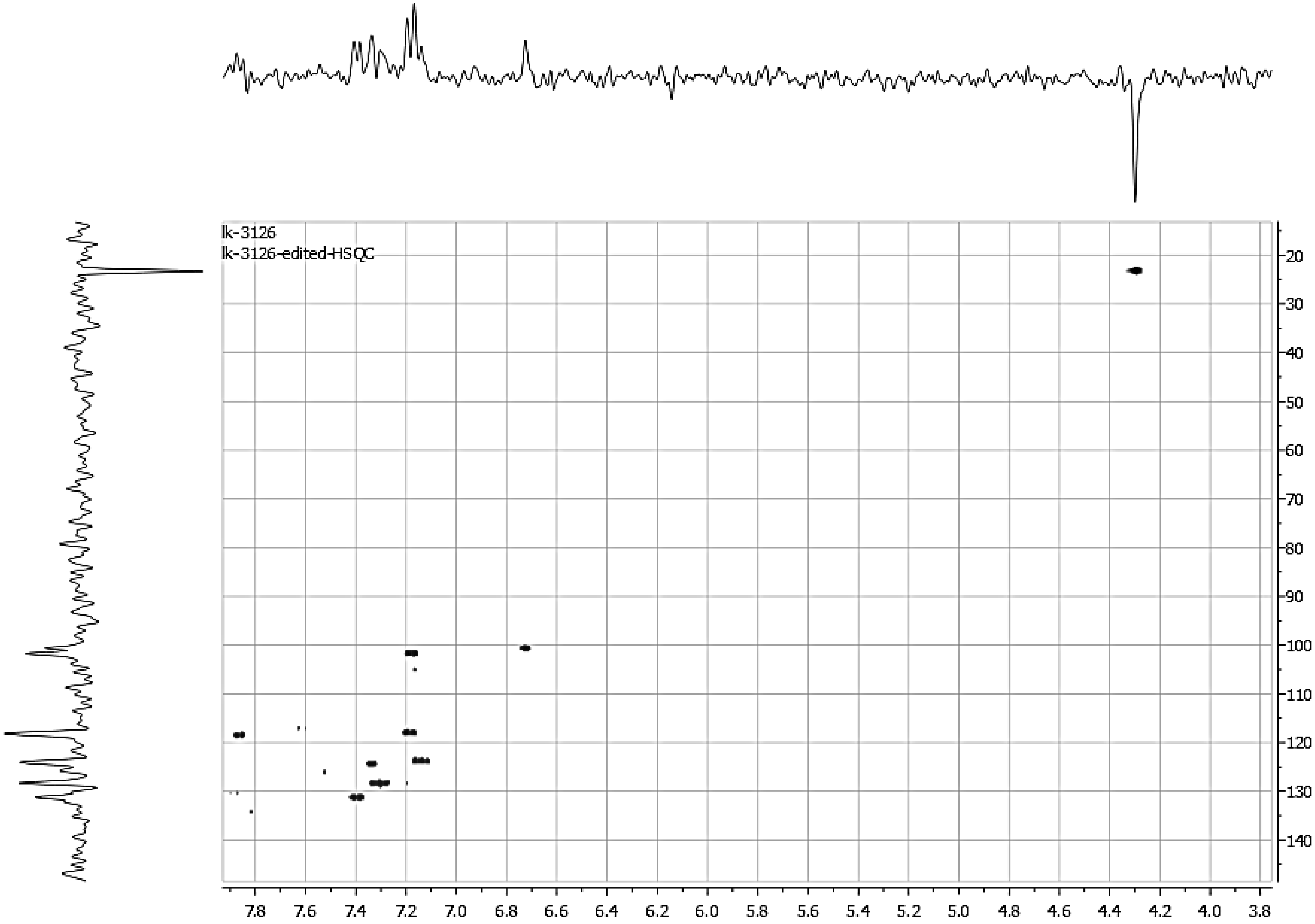
lk-3126
lk-3126-edited-HSQC
7.8 7.6 7.4 7.2 7.0 6.8 6.6 6.4 6.2 6.0 5.8 5.6 5.4 5.2 5.0 4.8 4.6 4.4 4.2 4.0 3.8
20 30 40 50 60 70 80 90 100 110 120 130 140

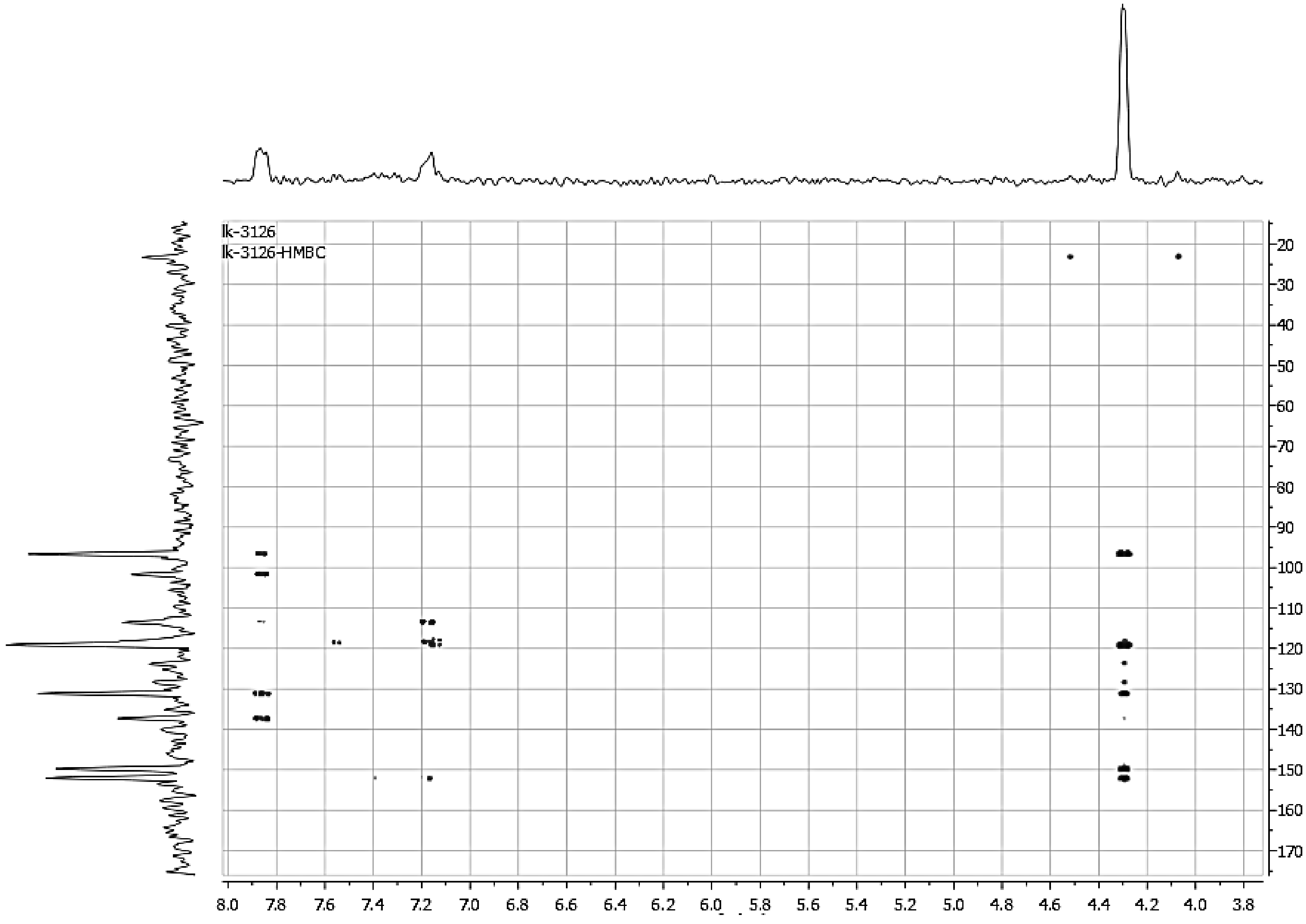
lk-3126
lk-3126-HMBC

8a

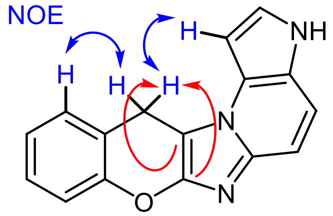

^{1}H, ^{13}C HMBC **8a**

Key cross-peaks and correlations in 2D NMR spectra for structure assignments indicated by arrows (blue – NOE; red – ^{1}H,^{13}C-HMBC)

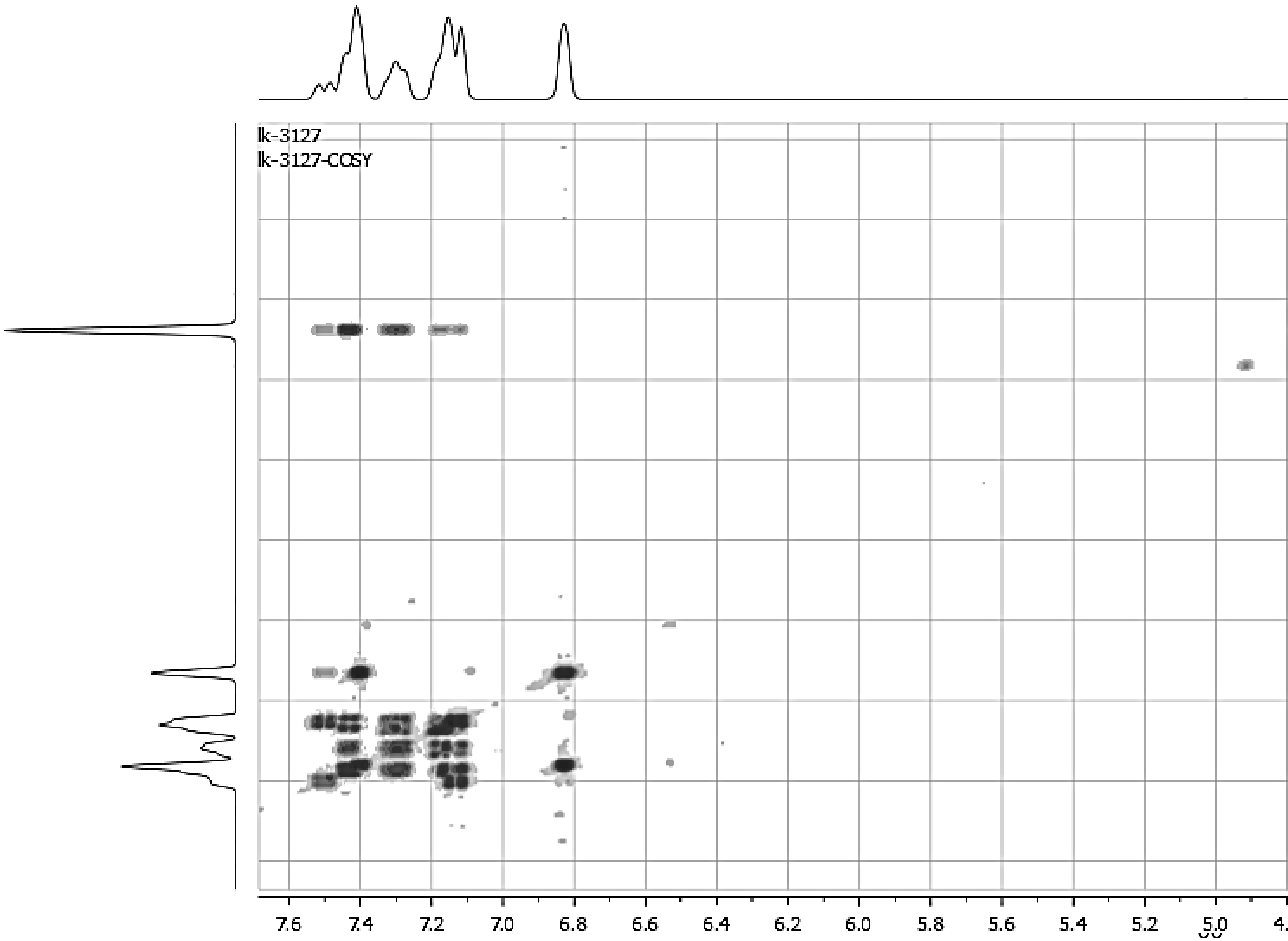

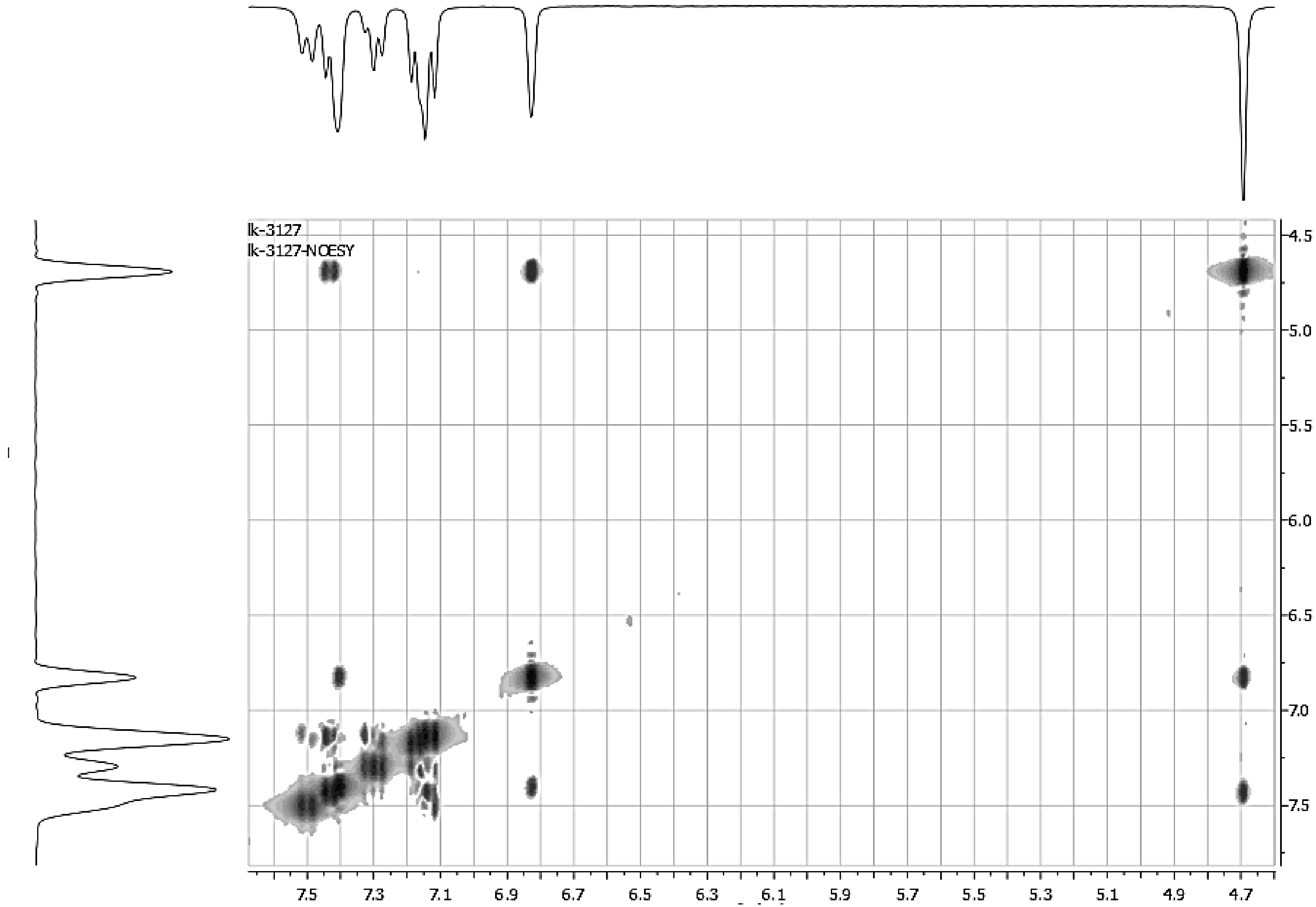
lk-3127
lk-3127-NOESY
4.5
5.0
5.5
6.0
6.5
7.0
7.5
7.5
7.3
7.1
6.9
6.7
6.5
6.3
6.1
5.9
5.7
5.5
5.3
5.1
4.9
4.7

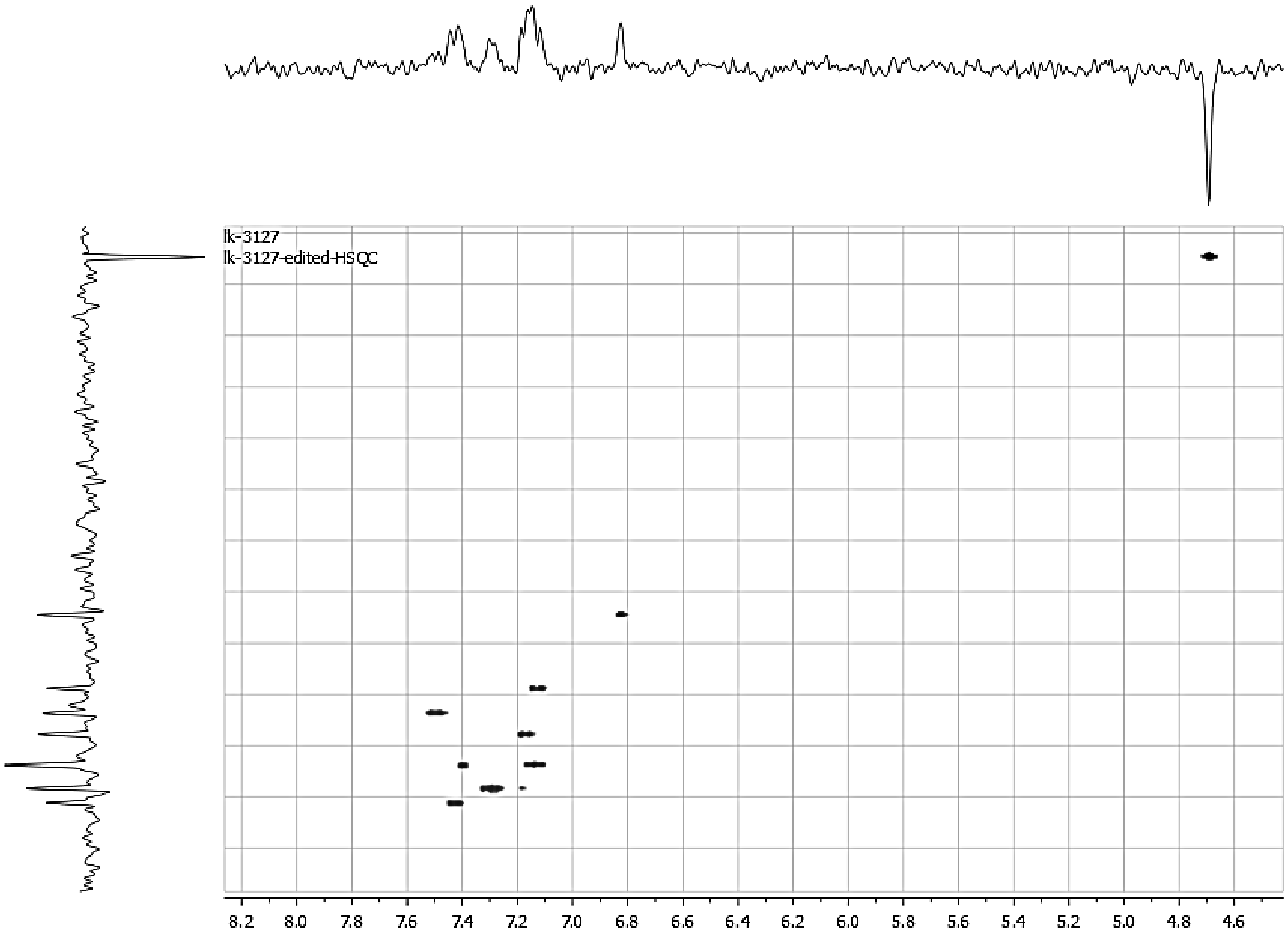
lk-3127
lk-3127-edited-HSQC
20
30
40
50
60
70
80
90
100
110
120
130
140
8.2
8.0
7.8
7.6
7.4
7.2
7.0
6.8
6.6
6.4
6.2
6.0
5.8
5.6
5.4
5.2
5.0
4.8
4.6

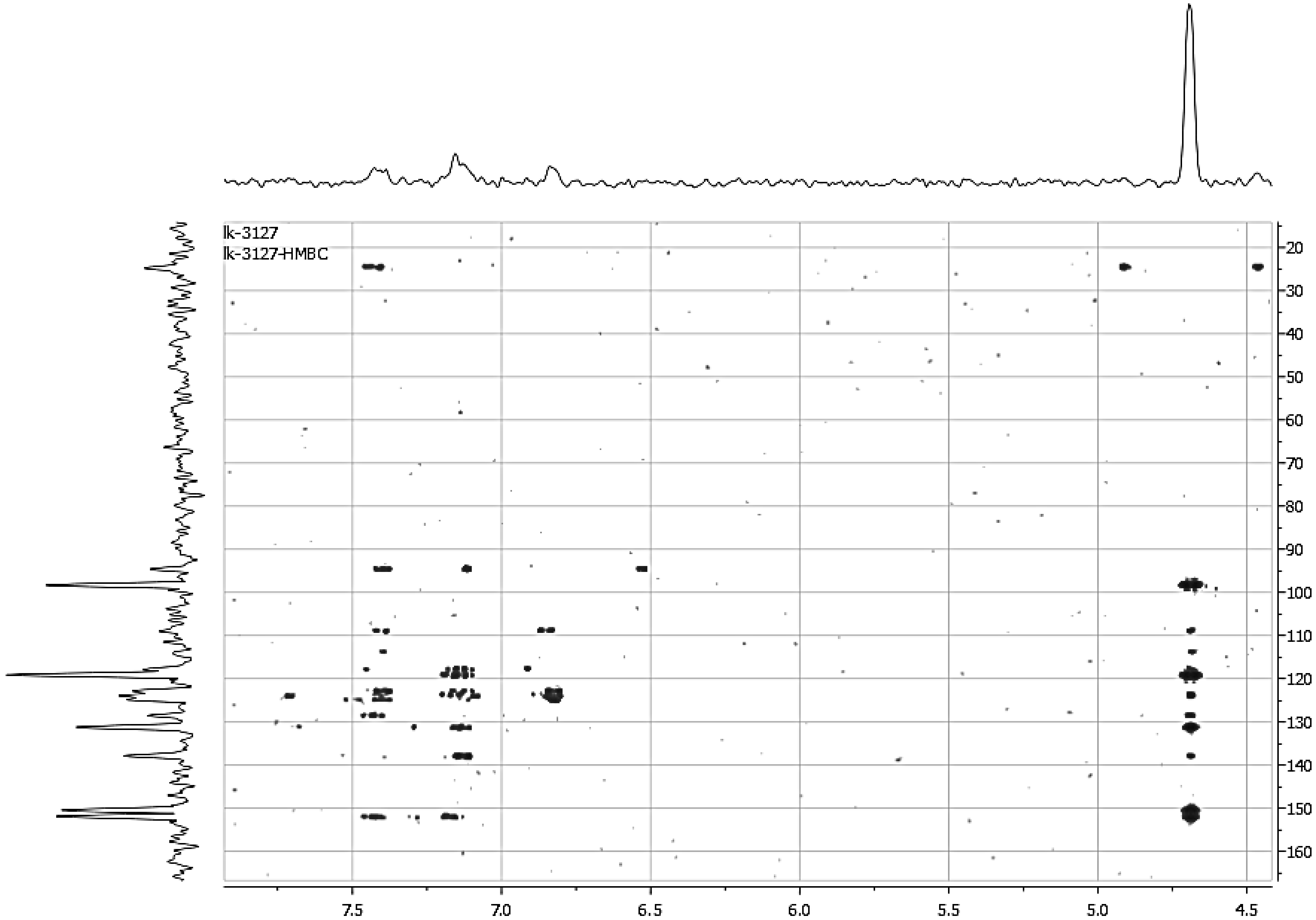
lk-3127
lk-3127-HMBC
20
30
40
50
60
70
80
90
100
110
120
130
140
150
160
7.5
7.0
6.5
6.0
5.5
5.0
4.5
